# Heterocyclic Suzuki–Miyaura coupling reaction of metalla-aromatics and mechanistic analysis of site selectivity†

**DOI:** 10.1039/d2sc05455h

**Published:** 2023-01-02

**Authors:** Zuzhang Lin, Yapeng Cai, Yaowei Zhang, Hong Zhang, Haiping Xia

**Affiliations:** a State Key Laboratory of Physical Chemistry of Solid Surfaces, Collaborative Innovation Center of Chemistry for Energy Materials (iChEM), College of Chemistry and Chemical Engineering, Xiamen University Xiamen 361005 China zh@xmu.edu.cn xiahp@sustech.edu.cn; b Shenzhen Grubbs Institute, Department of Chemistry, Southern University of Science and Technology Shenzhen 518055 China

## Abstract

Pd-catalyzed Suzuki–Miyaura cross-coupling is one of the most straightforward and versatile methods for the construction of functionalized arenes and heteroarenes but site-selective cross-coupling of polyhalogenated (hetero)arenes containing identical halogen substituents remains a challenging problem. Herein, we report a new candidate for heterocyclic Suzuki–Miyaura coupling reaction. This candidate has been applied in organometallic systems by combining classical aryl boronic acid reagents with non-classical heteroarenes. Experimental and computational studies of the mechanism of the reactions were performed, with an emphasis on the identity of the reactive species in the oxidative addition step and the nature of the precise site selectivity. The influence of both the aromaticity of the metalla-aromatic substrates and the steric and electronic properties of the halogenated sites are studied in detail.

## Abstract 2

We describe unique polyhalogenated heteroarene candidates for site-selective cross-coupling, which shows high catalytic performances in the functionalization of polycyclic metalla-aromatics with excellent photophysical properties.

## Graphical abstract

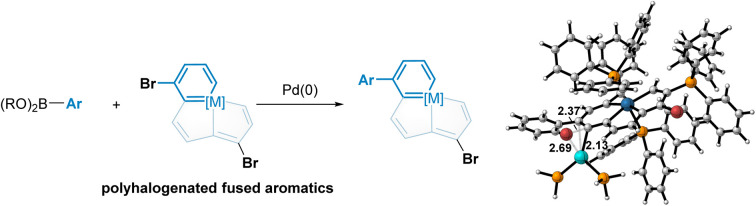

## Introduction

The ubiquitous nature of heteroarenes in molecules with diverse structures and properties has provoked considerable experimental and theoretical research.^1^ Metalla-aromatics are heteroarenes in which a metal fragment replaces a CH group of the aromatic ring. A significant number of isolable metalla-aromatics are currently known, and due to their unique optical and electronic properties they have rapidly evolved from laboratory curiosities to promising materials.^2^ With our ongoing interest in the chemistry of metalla-aromatics, the polycyclic metalla-aromatics, especially those centered by one bridgehead transition metal atom, have been examined as powerful catalysts,^3^ excellent electron transport layer materials in organic solar cells,^4^ and promising building blocks for molecular electronics.^5^ They have also been considered as chemotherapeutic drugs for photoactive therapy of hypoxic tumors,^6^ and as building blocks for metallopolymers with distinct properties.^7^ Due to their photophysical properties,^8^ these fused metalla-aromatic complexes are prominent structural motifs and thus their selective functionalization is of particular interest. Yet the complexes with extended conjugation, for example, aryl substituted metalla-aromatic complexes are rarely accessed by direct functionalization. A number of substituted metalla-aromatics, which offer a unique opportunity to tune the properties in metallacycle systems, were only synthesized by reactions of arylated organic substrates with metal complexes.^4,8*b*,9^

**Scheme 1:**
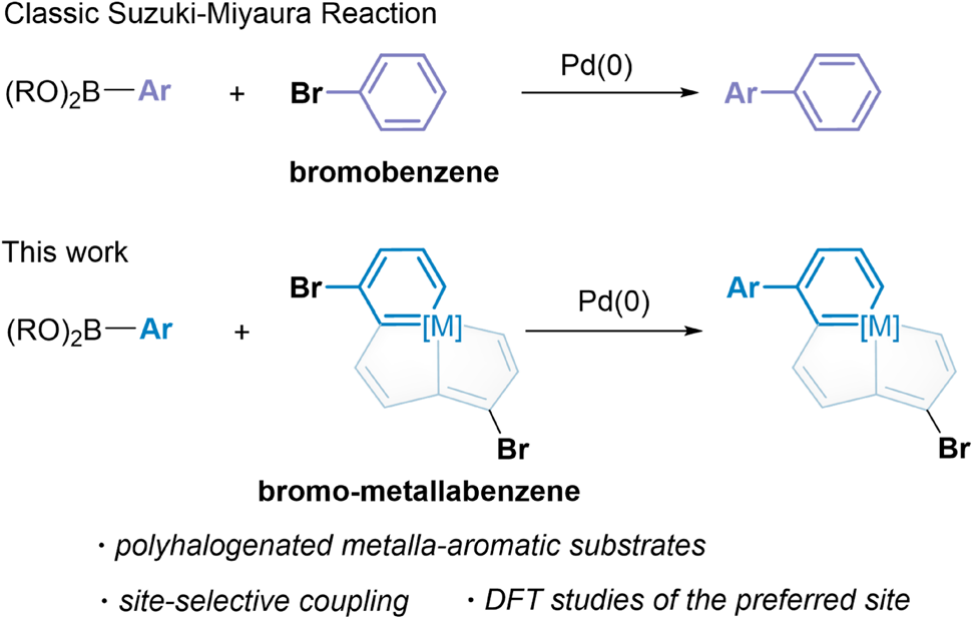
Suzuki–Miyaura reactions of metalla-aromatics.

Palladium-catalyzed cross-coupling reactions are a very powerful method to functionalize halogenated arenes by selectively replacing a halogen with a desired substituent.^10^ In particular, the Suzuki–Miyaura reaction which was awarded a Nobel Prize in 2010 has been extensively studied in a wide number of catalytic systems and applied to diversely functionalized arenes and heteroarenes.^11^ However, non-classical aromatics have never been introduced in transition metal-catalyzed cross-coupling reactions, to the best of our knowledge. As one of the most noticeable non-classical aromatics, metalla-aromatics are ideal substrates, because their bromination is easily manipulated.^8*i*,12^ We thus sought to extend the typical Suzuki–Miyaura reaction in the metalla-aromatic family, especially the polyhalogenated species, to discover an efficient method for the construction of highly substituted or functionalized metalla-aromatics. Herein, we report the successful application of polyhalogenated metalla-aromatic substrates for palladium-catalyzed Suzuki–Miyaura coupling and the first example of the behavior of a multiple fused-ring aromatic system in catalytic site-selective cross-coupling reactions (Scheme 1). The factors that enable the differentiation between carbon positions bearing identical halogen atoms have been examined by using theoretical calculations.

## Results and discussion

**Scheme 2:**
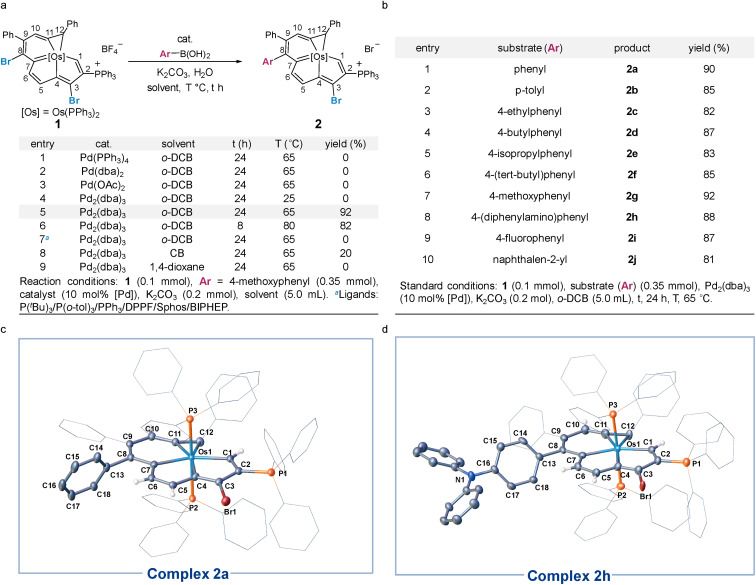
(a) Development of optimal condition. (b) Cross-coupling of 1 with aryl boronic acid. (c) X-ray molecular structure for the cation of complex 2a drawn with 50% probability level. (d) X-ray molecular structure for the cation of complex 2h drawn with 50% probability level. The hydrogen atoms on the phenyl group are omitted for clarity.

Our studies began with the catalytic cross-coupling reaction of the dihalogenated metalla-aromatic (1)^8*i*^ (Scheme 2). This compound was selected due to the two substituted bromine atoms located in different aromatic rings, *i.e.* metallabenzene and metallapentalene. We first examined the reaction of 1 with excess arylboronic acids under standard coupling reaction conditions (Scheme 2a). Initial success was achieved using Pd_2_(dba)_3_ (tris(dibenzylideneacetone)dipalladium) as the catalyst, potassium carbonate as the base, 4-methoxyphenyl boronic acid as the boron reagent and *o*-dichlorobenzene (*o*-DCB) as the solvent. In optimization studies, summarized in Scheme 2a, most of the conventional Pd catalysts such as Pd(PPh_3_)_4_, Pd(dba)_2_, and Pd(OAc)_2_ were found to be essentially inactive (entries 1–3). Lowering the reaction temperature from 65 to 25 °C led to almost no reaction with Pd_2_(dba)_3_ (entry 4). When the reaction temperature is raised to 65 °C, 92% yield is obtained (entry 5), but increasing the temperature to 80 °C (entry 6) only reduced the yield, suggesting that higher temperatures increase the rate of decomposition relative to the productive turnover. The effect of additional phosphine ligands on the reaction has been probed systematically.^13^ We found that the reactions have been restrained by adding additional phosphines, even PPh_3_ ligands, under the reaction condition in entry 5 (entry 7, see Section S29 in the ESI† for further details). We speculate that the trace decomposition of the metalla-aromatic substrates would provide sufficient PPh_3_ ligands to form catalytically active Pd species. The importance of the Pd/PPh_3_ ratios has been demonstrated in the literature,^14^ which fully analyzed the reason for the comparatively lower activity of the catalyst system in the presence of an excess amount of phosphines. When reaction was attempted in chlorobenzene (CB) or 1,4-dioxane, the yield decreased to 20% or zero, respectively (entry 8 and 9).

The scope and limitations of Pd-catalyzed cross-coupling reaction of 1 were investigated with respect to aryl boronic acid with a methoxy group located in different positions (see Section S31 in the ESI† for further details). Consistent with the classical Suzuki–Miyaura reaction,^15^ the reaction worked best with a minimized steric environment around the aryl group of boronic acid. High yields of 2 were obtained when an electron-donating methoxy was in the *para*-position. In addition, we found that the ratio of the dehalogenated byproduct increased sharply in the reaction with meta or *ortho*-substituents, albeit in a close reaction (see Section S31 in the ESI† for further details). Isotope-labeling experiments suggest that the hydrogen atom at the C8 position in the byproduct is derived from water (see Section S31 in the ESI† for further details). This result reinforces that the reaction is sensitive to steric effects.

We investigated the scope of this transformation with respect to the aryl boronic acids (Scheme 2b). The effect of *para*-substitution in the phenyl moiety was examined, and the reactions provided the corresponding complexes in excellent yields if the substituent was an alkyl, methoxyl, dimethylamino, or fluoro group. The reaction worked best with electron-rich boronic acids such as 4-methoxyphenyl boronic acid (2g). Electron-deficient boronic acids, with the exception of 4-fluorophenyl boronic acid (2i), produced only a trace of the cross-coupling product. As monitored *in situ* by NMR, the metalla-aromatic substrate (1) reacts sluggishly and decomposes slowly when treated with an aryl boronic acid bearing an electron withdrawing substituent, such as trifluoromethyl. This might also diminish the catalytic borylation yield (see Section S32 in the ESI† for further details). While *o*-naphthylboronic acid was found to be a good partner, affording 2j in 81% yield, neither pyrene nor heterocyclic boronic acids are tolerated as substrates under these reaction conditions. The solid-state structures of 2a and 2h were authenticated by X-ray diffraction (Scheme 2c and d). In both cases, the six-membered metallabenzene ring and the phenyl ring are linked together at the C8 atom and are nearly perpendicular to each other (the torsional angles between the phenyl group and metallabenzene ring are 72° and 65°). The C–C bond and Os–C bond lengths of the metalla-aromatic rings lie within the range of typical bonds in previously reported metalla-aromatics.^8*i*^ The structural parameters of 2a and 2h compare well with those of substrate 1 ^8*i*^ (see Section S33 in the ESI† for further details).

**Scheme 3:**
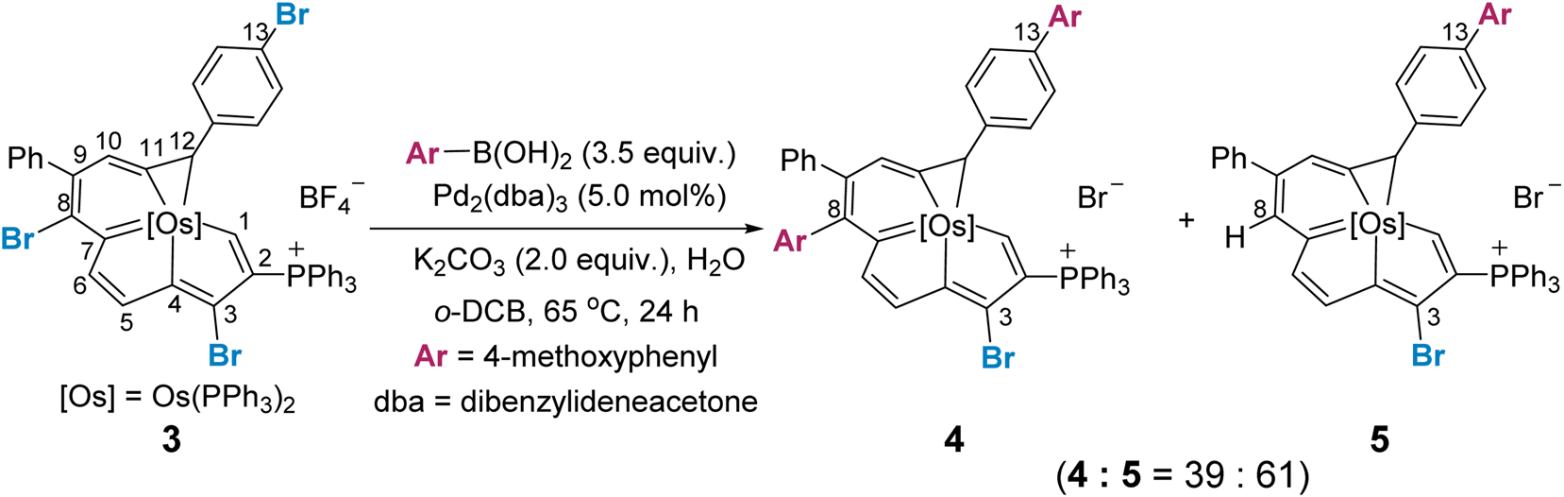
Use of a trihalogenated metalla-aromatic to extend the Suzuki–Miyaura coupling reaction for mechanistic consideration.

We reasoned that the selectivity-determining step of the cross coupling reaction would be influenced by the steric and electronic properties of the fused aromatic rings, and a site selective bias might appear when one metallacycle is inherently more reactive. We thus examined the reaction of a trihalogenated metalla-aromatic (3) under the optimal coupling reaction conditions (Scheme 3). The reaction provided the cross-coupling product (4), in which both the C8 of the metalla-benzene ring and C13 of the benzene ring are substituted, together with the dehalogenated byproduct (5) by protonation (see Section S36 in the ESI† for further details). A similar dehalogenation has been shown in the reaction system without or lacking boronic acid.^16^ Also pertinent to mention is that the degradation of the substrate, leading to the some complicated P-containing species (detected by ^31^P NMR), was observed (see Section S37 in the ESI† for further details), when the ratio of 3 : ArB(OH)_2_ was 1 : 1. It has been well established that the dehalogenation process could be increased when the *in situ* formed tetracoordinate boronate is lacking.^16^ Since the metalla-aromatic substrate 1 or 3 decomposed slowly under reaction conditions, *i.e.* heating with a base, decomposition rather than mono-arylation is thus seen after *ca.* 24 h at 65 °C in the presence of one equivalent of aryl boronic acid.

**Fig. 1:**
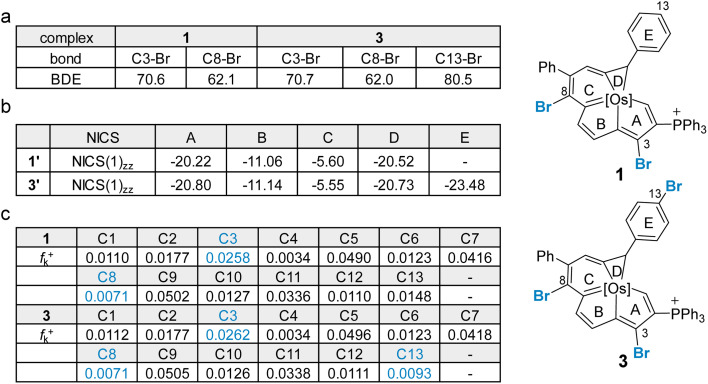
(a) BDEs calculated at the B3LYP/def2-TZVP//B3LYP/6-31G(d) level (SDD for Os, Br, and P), are given in kcal mol^−1^. (b) NICS value of complexes 1′ and 3′ (the PPh_3_ is simplified as PH_3_ in 1′ and 3′), based on the B3LYP/def2-TZVP level. (c) Condensed Fukui function at different sites in complexes 1 and 3, based on the M06L/def2-TZVP level.

In general, Suzuki–Miyaura cross-coupling reactions exhibit three key steps, oxidative addition,^17^ transmetalation and reductive elimination. The oxidative addition is the rate- and product-determining step in most cases.^17,18^ Thus, site selectivity in cross-coupling reactions is largely determined by the relative electrophilicities of the carbons bearing the halogen substituents. The electronic structures of 1 and 3 were first investigated by density functional theory (DFT) calculations. Geometry optimization and frequency calculations were performed at the B3LYP/6-31G(d) level of theory, and were in satisfactory agreement with the reported crystallographic data of 1 and 3 (see Section S39 in the ESI† for further details).^8*i*^ The C–Br bond dissociation energy (BDE)^19^ in 1 and 3 was computed at the UB3LYP/6-31G(d) level (Fig. 1a). Consistent with the experimental findings, the C8–Br bond in each of two systems has the lowest calculated BDE of 62.1 kcal mol^−1^ for the dihalogenated metalla-aromatic (1) and 62.0 kcal mol^−1^ for the trihalogenated metalla-aromatic (3). The lowest BDE associated with the C8 position might facilitate the selective installation of a substituent at the C8 position. Nucleus-independent chemical shift (NICS) calculations^20^ were performed to assess the aromatic character of the fused rings (Fig. 1b). NICS(1)_*zz*_ indices were used because the value considers shielding at 1 Å above the ring where π-contributions are dominant. The computed values indicate that the metallabenzene ring of metalla-aromatic 1 or 3 possesses a relatively weaker aromaticity.

Since electronic effects usually control the reaction selectivity, the condensed Fukui function^21^ was also employed to predict the most electrophilic sites, the atoms with the largest *f*_k_^+^ value, in 1 and 3 (Fig. 1c). C3 has the largest *f*_k_^+^ value in both 1 and 3, but strong steric influences might override the electronic effect and perturb the innate reactivity because C3 is sterically encumbered by the adjacent bulky triphenylphosphonium substituent. The computed *f*_k_^+^ values indicate that the second most favorable site for electrophilic attack in compound 3 is C13, consistent with the structure of the isolated product (4). Along these lines, we speculate that the steric effect is an important factor affecting the competition for the cross-coupling site in 1 or 3.

**Fig. 2:**
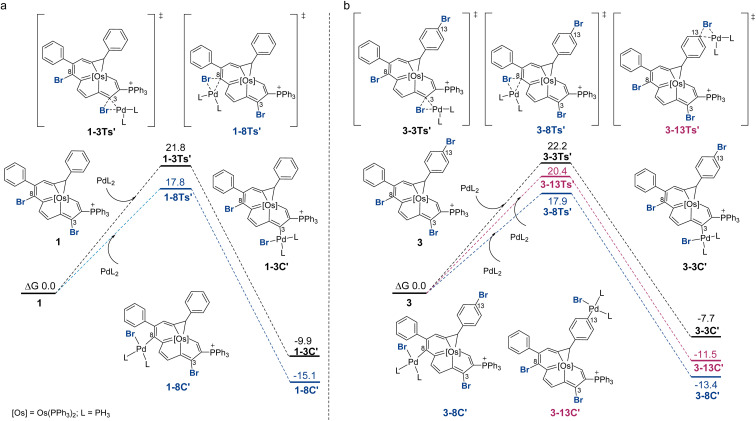
DFT-computed Gibbs free energy profile for the oxidative addition steps. (a) Reaction between 1 and PdL_2_ at C3 and C8. (b) Reaction between 3 and PdL_2_ at C3 or C8 or C13, *L* = PH_3_. All energies were computed at the level B3LYP-D3/def2-TZVP/PCM(*o*-DCB)//B3LYP/6-31G(d)(SDD for Os, Br, P), and are given in kcal mol^−1^.

Fig. 2 displays the reaction profiles for the oxidative addition processes in 1 and 3. A bisphosphine model complex Pd(PH_3_)_2_ was chosen, and the labels of the model compounds are followed by the prime (′) symbol to distinguish them from the corresponding experimental compounds. For both 1 and 3, the corresponding free energy barrier for C8 is reasonably smaller than that for C3, which is consistent with the experimental results, favoring the oxidative addition at C8 by 4.0 and 4.3 kcal mol^−1^, respectively. The C3 product is less stable due to an unfavorable catalyst–substrate arrangement in which the bulky phosphonium group is adjacent to the more crowded C3 site. For comparison, when trisubstituted complex 3 was employed, the oxidative addition step for C13 is calculated to have a barrier of 20.4 kcal mol^−1^. The C8 product is more thermodynamically favored than the C13 product. DFT predicts that the reaction at C13 starting with C8 product 3-8C′ could be feasible with a barrier of 17.0 kcal mol^−1^ (see Section S42 in the ESI† for further details). This prediction is consistent with experimental observations shown in Scheme 3 where the diarylation product 4 can be obtained. A competition experiment was carried out by mixing 1.0 eq. compound 1 and 1.0 eq. bromobenzene with 1.0 eq. 4-methoxyphenyl boronic acid. Only 2g was present in the reaction mixture, as indicated by HRMS (see Section S43 in the ESI† for further details), which further established that the reaction at C8 is easier.

The 3-centered mechanism with monoligated Pd models for oxidative addition has been demonstrated very recently.^22^ Additional calculation using monoligation model Pd(PH_3_) also proved to be in good agreement with the experimental results, and one of the energy profiles was calculated with Pd(PPh_3_) for comparison (see Section S44 in the ESI† for further details). We also calculated the oxidative addition processes with the model chloro-substituted compounds. From the DFT calculation results, we can see the same trend where the C3 product has a higher reaction barrier than the C8 product (see Section S46 in the ESI† for further details).

**Fig. 3:**
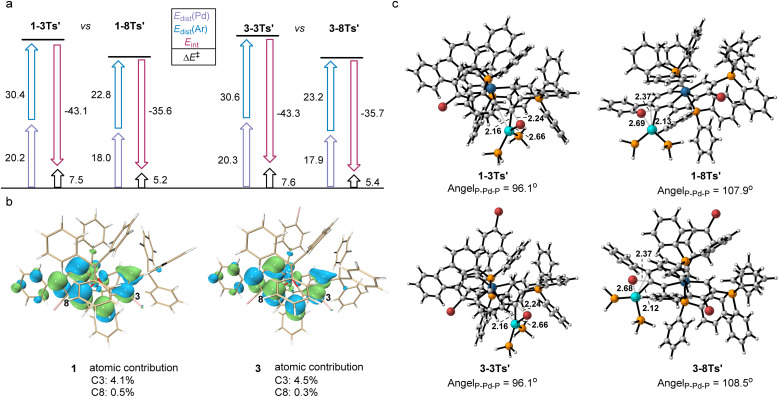
(a) Distortion/interaction analysis. All energies were computed at the level B3LYP-D3/def2-TZVP/PCM(*o*-DCB), and are given in kcal mol^−1^. (b) LUMO of 1 and 3. (c) TSs for oxidative insertion into C3–Br and C8–Br.

A distortion/interaction (D/I) analysis^23^ was employed to further analyze the factors that control the oxidative addition of 1 and 3. The activation energy (Δ*E*^‡^) has been divided into the distortion energy (Δ*E*_dist_, required to distort substrate 1/3 and the Pd catalyst into the geometries in the transition state (TS)) and the energy of interaction (Δ*E*_int_, determined from the interaction strength between the distorted 1/3 and the Pd catalyst as they reach the TS). As shown in Fig. 3a, the relative stabilities of oxidative addition TSs (Δ*E*^‡^) at C3 or C8 in 1 and 3, which lead to the preferred site of oxidative addition, are determined by using both Δ*E*_dist_ and Δ*E*_int_. Substrate 1 was chosen as the representative in the following discussion since the energies in both substrates differ by less than 0.5 kcal mol^−1^. The energy required to distort the metalla-aromatic substrate at the C3 position is significantly higher than that for the C8 position (ΔΔ*E*_dist_(Ar) = 7.5 kcal mol^−1^), and follows the order of BDEs (70.6 kcal mol^−1^ for C3 and 62.1 kcal mol^−1^ for C8). There is a relatively small difference between the Δ*E*_dist_ of the Pd catalyst for C3 or C8 (ΔΔ*E*_dist_(Pd) = 2.3 kcal mol^−1^). The interaction energy for the C3 position (−43.1 kcal mol^−1^) is much greater than that for the C8 position (−35.6 kcal mol^−1^), although the cross-coupling has been shown experimentally to occur exclusively at the 8-position. The interaction energies have previously been attributed to the basis frontier molecular orbital (FMO) interactions between the aromatic substrate and the catalyst, *i.e.* the overlap between the aromatics π* lowest unoccupied molecular orbitals (LUMOs) and the Pd d_*xy*_ highest occupied molecular orbitals (HOMOs).^24^ We thus examined the LUMO diagrams of metal-aromatic species 1 and 3 (Fig. 3b). Both LUMOs of metal-aromatic species distribution at the C3 position are much more significant than that at the C8 (4.1% > 0.5% at C8 for 1; 4.5% > 0.3% at C8 for 3), suggesting the stronger FMO interactions. According to Fig. 3a, the barrier differences originate primarily from the much greater distortion energies of the C3 position, although interaction energies are biased to stabilize in the oxidative addition transition state (TS) for the C3 position. As shown in Fig. 3c, the breaking C–Br bond length in the reaction of 1 is longer in the TS for attack at the 8-position (2.37 Å) relative to that at the 3-position (2.24 Å). The same trend is also observed in substrate 3 in which activation at the 8-position occurs later than that at the 3-position. In addition, both the L–Pd–L and the C–Br at the 8-position are more easily distorted than those at the 3-position.

The transition structures shown in Fig. 3c indicate that this is probably due to steric repulsions of the phosphonium group on the C2. Therefore, the steric impediment overcomes the stronger FMO interactions for the C3 position, rendering the C8 cross-coupling product more favorable.

## Conclusion

We have described the Suzuki–Miyaura coupling reaction of fused aromatics bearing multiple identical halogens, which delivers the aryl substituted metalla-aromatics site-selectively. The efficiency and site selectivity of the reactions described here are striking in light of the challenges associated with polyhalogenated fused-aromatic substrates. Mechanistic studies have elucidated the factors contributing to site selectivity in the reactions of the complex fused-ring system with a metalla-benzene ring and a metalla-pentalene ring. We show that the relatively higher electrophilicity of the halogenated carbon in the metalla-pentalene ring is subject to a steric control of regiochemistry, and therefore favors the coupling reaction in the metalla-benzene ring. Given the promising performance of fused metalla-aromatics, this coupling protocol is expected to present broad synthetic possibilities for selective functionalization of these nonclassical heteroarenes, thus expanding the functional materials using metallacycles as building blocks. These observations have also revealed a new facet of the heterocyclic Suzuki–Miyaura coupling reaction and may facilitate applications in future synthesis design involving fused aromatic substrates.

## Data availability

The data that support the findings of this study are available in the ESI† or on request from the corresponding author.

## Author contributions

H. Z. and H. X. designed and conceived the project. Z. L. and Y. Z. conducted all the synthetic reactions. Z. L. recorded all NMR and HRMS data. Z. L. solved all X-ray structures. Z. L., H. Z. and H. X. analyzed and interpreted the experimental data. Y. C. designed and performed the theoretical calculations. All of the authors discussed the results and contributed to the preparation of the manuscript.

## Conflicts of interest

There are no conflicts to declare.

## Supplementary Material

SC-014-D2SC05455H-s001

SC-014-D2SC05455H-s002

SC-014-D2SC05455H-s003
