# Supplementary material for: Heterocyclic Suzuki–Miyaura coupling reaction of metalla-aromatics and mechanistic analysis of site selectivity
Source: Chem Sci. 2023 Jan 2;14(5):1227–33. doi: 10.1039/d2sc05455h (PMC9891379; doi:10.1039/d2sc05455h)
Supplement: SC-014-D2SC05455H-s002 [file SC-014-D2SC05455H-s002.pdf]

Electronic Supplementary Material (ESI) for Chemical Science.
This journal is © The Royal Society of Chemistry 2022

Supporting Information

Zuzhang Lin,[†,⊥] Yapeng Cai,[†,⊥] Yaowei Zhang[†], Hong Zhang[†]* and Haiping Xia[†,‡]*

[†]State Key Laboratory of Physical Chemistry of Solid Surfaces, College of Chemistry and Chemical Engineering, Xiamen University, Xiamen, Fujian 361005, China

[‡]Shenzhen Grubbs Institute, Department of Chemistry, Southern University of Science and Technology, Shenzhen 518055, China

[⊥]These authors contributed equally to this work

*Corresponding Author: zh@xmu.edu.cn (Hong Zhang); hpxia@xmu.edu.cn (Haiping Xia)

## Contents

**1. Experimental Procedures and NMR spectra**

**2. Ligand Screen**

**3. Aryl Boronic Acid with Methoxy Group Located in Different Positions**

**4. Isotopic-labeling Experiments**

**5. Reaction of 1 with (4-(trifluoromethyl)phenyl) boronic acid.**

**6. X-ray Crystallographic Analysis**

**7. Reaction of 3 with 4-methoxyphenyl boronic acid.**

**8. Reaction of 3 with 1 equiv. 4-methoxyphenyl boronic acid**

**9. Reaction of 3 with 2.5 equiv. 4-methoxyphenyl boronic acid**

**10. Computational Calculations**

**11. Oxidative addition of 3-8C' and $PdL_2$ at C13.**

**12. Mix 1.0 equiv. compound 1 and 1.0 equiv. bromobenzene with 1.0 equiv. p-methoxyphenylboronic acid.**

**13. Oxidative addition of 3 and PdL (L = $PH_3$)**

**14. Oxidative addition of 3 and PdL (L = $PPh_3$)**

**15. The oxidative addition processes with the model chloro-substituted compounds (3'')**

**16. Proposed Mechanism**

**17. References**

## 1. Experimental Procedures and NMR spectra

### General information

All air-sensitive or water-sensitive reactions were carried out using the Schlenk technique under inert atmosphere. Solvents were distilled under nitrogen from sodium/benzophenone (hexane and diethyl ether) or calcium hydride (dichloromethane) prior to use. The chemical reagents were obtained from Acros, Innochem, or other commercial resources and used without further purification. Column chromatography was performed on neutral alumina (200–300 mesh) in air. NMR spectra was collected on a Bruker Advance II 500 spectrometer, a Bruker Advance III 600 spectrometer at RT. $^{1}H$ and $^{13}C\{^{1}H\}$ NMR chemical shifts (δ) are relative to tetramethylsilane, and $^{31}P\{^{1}H\}$ NMR chemical shifts are relative to 85% $H_3PO_4$. Elemental analysis (EA) data were collected on a Vario EL III elemental analyzer. The high-resolution mass spectra (HRMS) experiments were performed on a Bruker En Apex Ultra 7.0 T and Agilent 1290-6545XT.

## Preparation of 2a

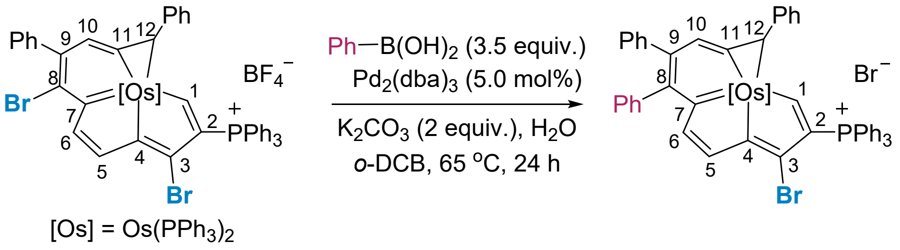


**1** **2a**

To a Schlenk tube were added **1** (91.6 mg, 0.06 mmol), phenylboronic acid (25.6 mg 0.21 mmol), $Pd_2(dba)_3$ (5 mol%, 2.75 mg) and $K_2CO_3$ (16.6 mg, 0.12 mmol) and 3 mL of 1,2-dichlorobenzene in nitrogen atmosphere. After the tube was charged with argon through a freeze-pump-thaw cycle for three times, the reaction mixture was stirred for 24 h at 65 °C under argon atmosphere. The reaction system was dried by anhydrous sodium sulfate, and then purified by column chromatography on neutral alumina with dichloromethane/ methanol = 20:1 as eluent to give the desired as yellow-green solid. (**2a**, 77.6 mg, 90%).

$^1$H NMR plus $^1$H-$^{13}$C HSQC (500.2 MHz, $CD_2Cl_2$): $\delta$= 13.3 (d, $J_{P\text{-}H}$ = 21.8 Hz 1H, H1), 7.2 (s,1H, H5), 7.7 (s, 1H, H10), 7.3 ppm (s,1H, H6). $^{31}$P NMR (202.5 MHz, $CD_2Cl_2$): $\delta$= 14.1 (t, $J_{P\text{-}P}$ = 6.2 Hz C*P*Ph$_3$), –9.2 (dd, $J_{P\text{-}P}$ = 250.5 Hz, $J_{P\text{-}P}$ = 4.7 Hz, Os*P*Ph$_3$), –17.5 ppm (dd, $J_{P\text{-}P}$ = 250.5 Hz, $J_{P\text{-}P}$ = 7.5 Hz, Os*P*Ph$_3$). $^{13}$C NMR plus DEPT-135, $^1$H-$^{13}$C HSQC and $^1$H-$^{13}$C HMBC (125.8 MHz, $CD_2Cl_2$): $\delta$= 233.7 (t, $J_{P\text{-}C}$ = 7.5 Hz, C7), 218.5 (t, $J_{P\text{-}C}$ = 4.5 Hz, C11), 207.8 (t, $J_{P\text{-}C}$ = 19.7 Hz, C1), 191.7 (dt, $J_{P\text{-}C}$ =20.8 Hz, $J_{P\text{-}C}$ = 4.7 Hz, C4), 161.5 (s, C6), 157.7 (s, C5), 143.9 (s, C9), 139.3.0 (s, C8), 120.3 (d, $J_{P\text{-}C}$ = 87.2 Hz, C2), 117.3 (s, C10), 109.7 (d, $J_{P\text{-}C}$ = 25.7 Hz, C3), 14.2 ppm (s, C12). Elemental analysis calcd (%) for $C_{84}H_{65}Br_2OsP_3$: C 66.49, H 4.32; found: C 66.59, H 4.52. HRMS (ESI): m/z calcd for $[C_{84}H_{65}BrOsP_3]^+$, 1437.3081; found: 1437.3069.

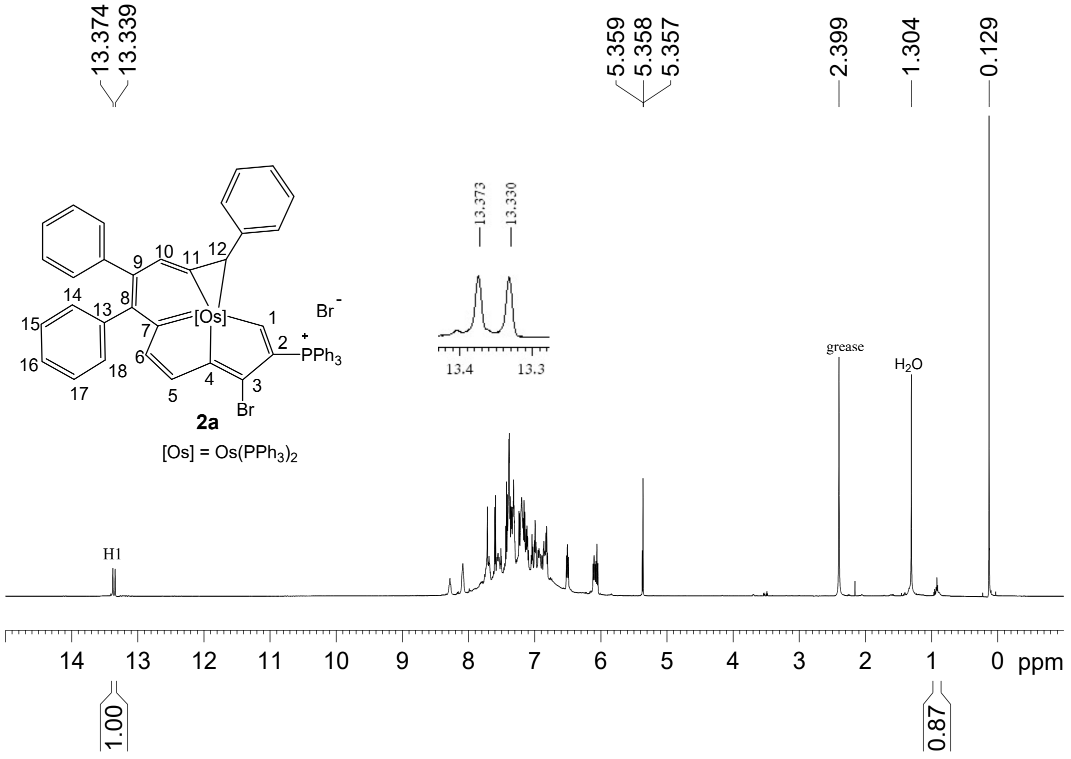


**Figure S1** The $^{1}$H NMR (500.2 MHz, $CD_2Cl_2$) spectrum for complex **2a**.

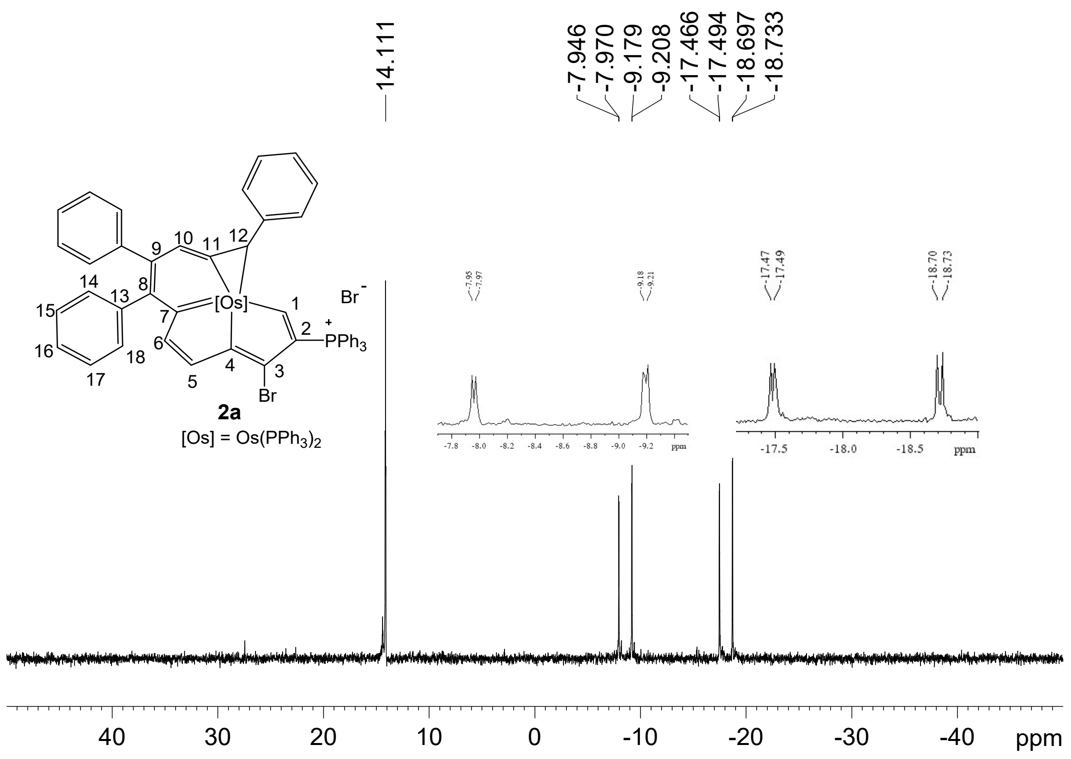


**Figure S2** The $^{31}$P{$^{1}$H} NMR (202.5 MHz, $CD_2Cl_2$) spectrum for complex **2a**.

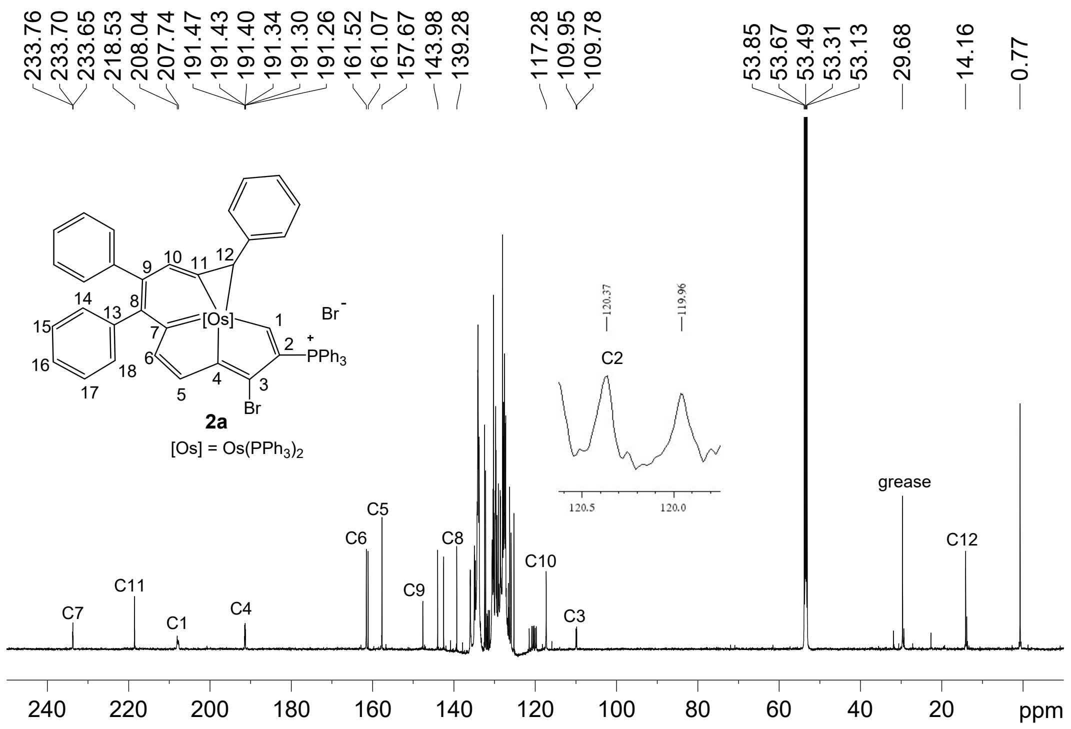


**Figure S3** The $^{13}$C NMR (125.8 MHz, $CD_2Cl_2$) spectrum for complex **2a**.

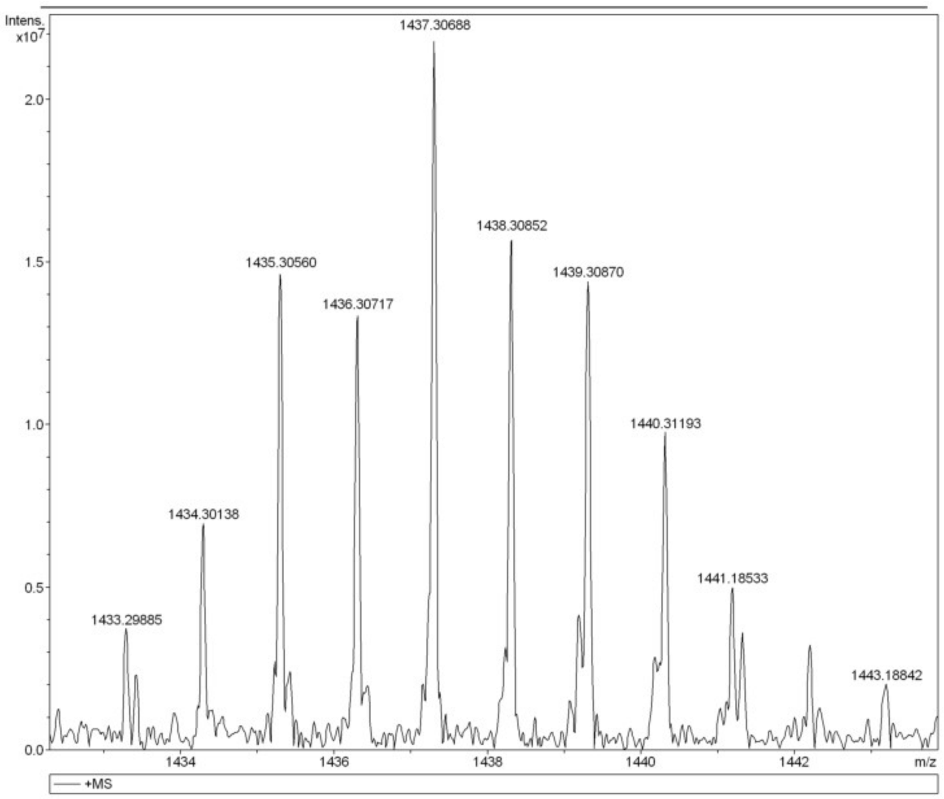


**Figure S4** Positive-ion ESI-MS spectrum of **2a** measured in methanol.

## Preparation of **2b**

**2b**: the synthetic procedure is similar to that for **2a**. **1** (91.6 mg, 0.06 mmol), *p*-tolylboronic acid (28.53 mg 0.21 mmol), $Pd_2(dba)_3$ (5 mol%, 2.75 mg) and $K_2CO_3$ (16.6 mg, 0.12 mmol) and 3 mL of 1,2-dichlorobenzene, afforded as yellow-green solid (**2b**, 74.0 mg, 85%). $^1$H NMR plus $^1$H-$^{13}$C HSQC (600.1 MHz, $CD_2Cl_2$): $\delta$= 13.3 (d, $J_{P\text{-}H}$ = 22.15 Hz 1H, H1), 7.2 (s,1H, H5), 7.6 (s, 1H, H10), 7.3 ppm (s,1H, H6), 2.4 ppm (s,3H, H19). $^{31}$P NMR (242.9 MHz, $CD_2Cl_2$): $\delta$= 14.0 (t, $J_{P\text{-}P}$ = 5.6 Hz C*P*Ph$_3$), –8.5 (dd, $J_{P\text{-}P}$ = 254.5 Hz, $J_{P\text{-}P}$ = 5.2 Hz, Os*P*Ph$_3$), –18.1 ppm (dd, $J_{P\text{-}P}$ = 250.5 Hz, $J_{P\text{-}P}$ = 6.3 Hz, Os*P*Ph$_3$). $^{13}$C NMR plus DEPT-135, $^1$H-$^{13}$C HSQC and $^1$H-$^{13}$C HMBC (150.9 MHz, $CD_2Cl_2$): $\delta$= 234.2 (t, $J_{P\text{-}C}$ = 7.2 Hz, C7), 218.2 (t, $J_{P\text{-}C}$ = 4.0 Hz, C11), 207.8 (t, $J_{P\text{-}C}$ = 21.0 Hz, C1), 191.3 (dt, $J_{P\text{-}C}$ =19.9 Hz, $J_{P\text{-}C}$ = 5.6 Hz, C4), 161.6 (s, C6), 157.5 (s, C5), 144.1 (s, C9), 139.2 (s, C8), 120.3 (d, $J_{P\text{-}C}$ = 89.2 Hz, C2), 117.3 (s, C10), 109.7 (d, $J_{P\text{-}C}$ = 24.5 Hz, C3), 20.9 ppm (s, C19), 14.0 ppm (s, C12). Elemental analysis calcd (%) for $C_{85}H_{67}Br_2OsP_3$: C 66.67, H 4.41; found: C 67.01, H 4.18. HRMS (ESI): m/z calcd for $[C_{85}H_{67}BrOsP_3]^+$, 1451.3237; found: 1451.3251.

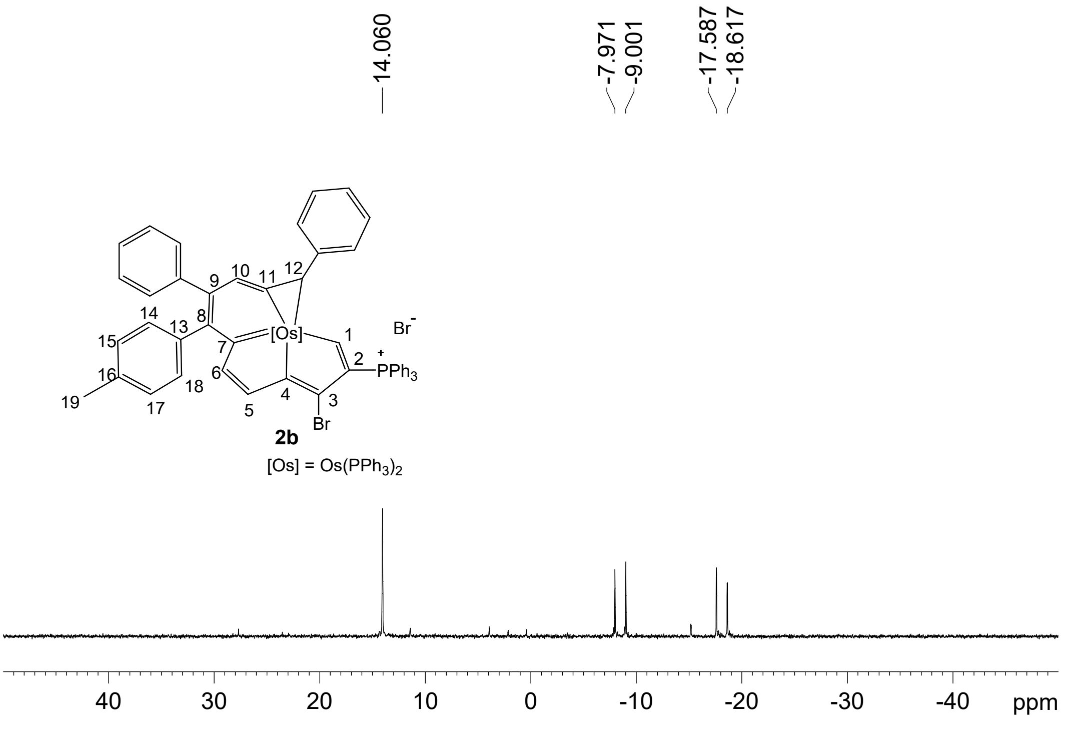


**Figure S5** The $^{31}$P{$^1$H} NMR (242.9 MHz, $CD_2Cl_2$) spectrum for complex **2b**.

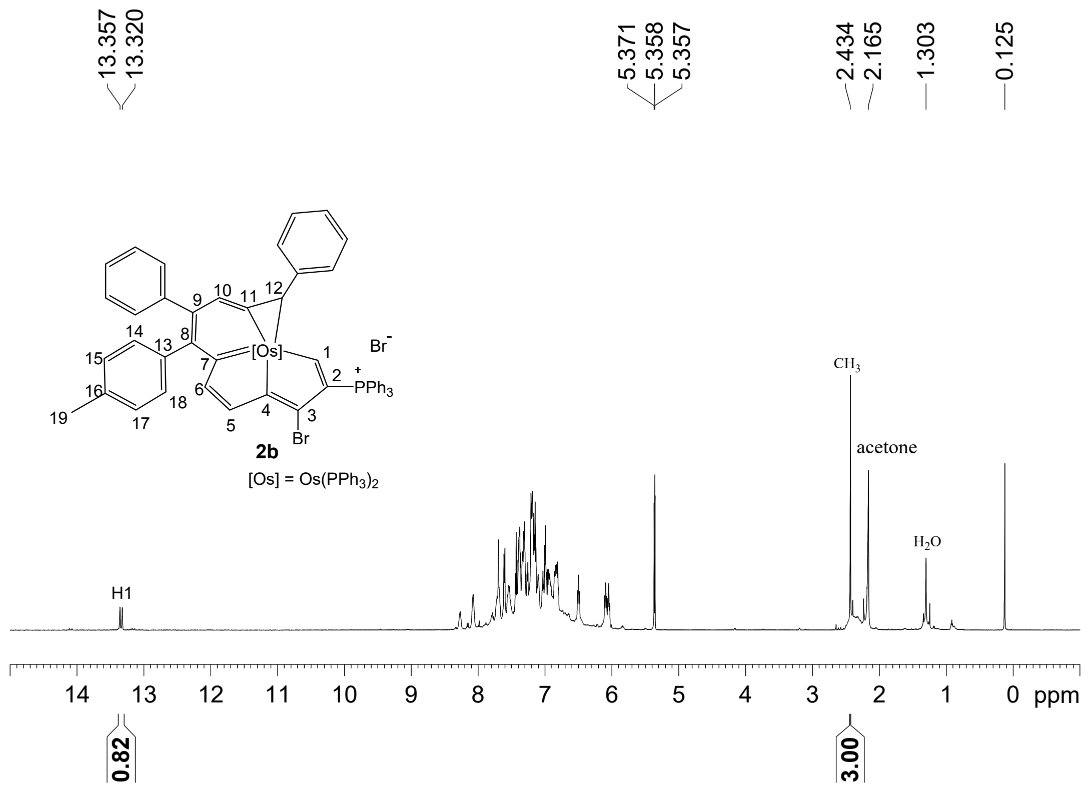


**Figure S6** The $^{1}H$ NMR (600.1 MHz, $CD_2Cl_2$) spectrum for complex **2b**.

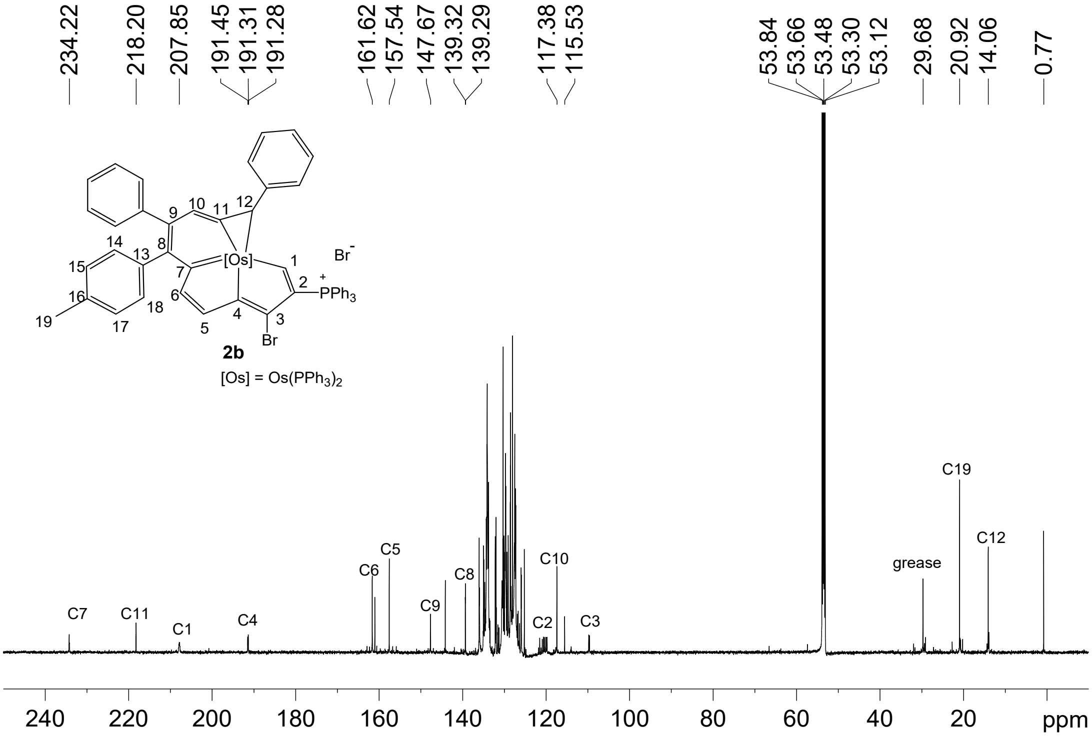


**Figure S7** The $^{13}C$ NMR (150.9 MHz, $CD_2Cl_2$) spectrum for complex **2b**.

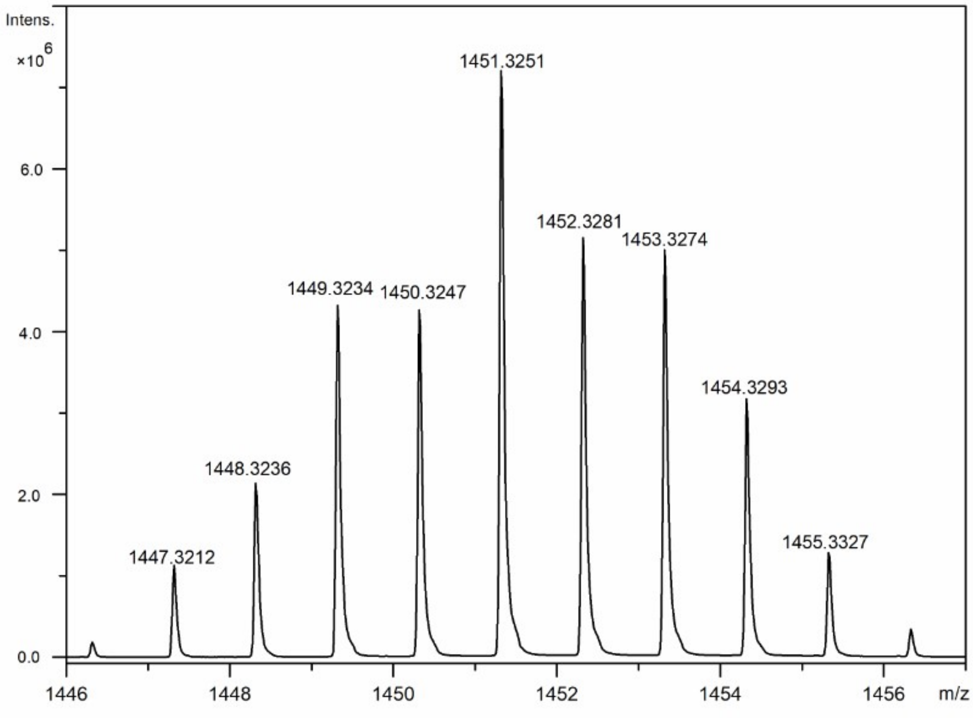


**Figure S8** Positive-ion ESI-MS spectrum of **2b** measured in methanol.

## Preparation of **2c**

**2c**: the synthetic procedure is similar to that for **2a**. **1** (91.60 mg, 0.06 mmol), (4-ethylphenyl) boronic acid (31.49 mg 0.21 mmol), $Pd_2(dba)_3$ (5 mol%, 2.75 mg) and $K_2CO_3$ (16.6 mg, 0.12 mmol) and 3 mL of 1,2-dichlorobenzene, afforded as yellow-green solid (**2c**, 72.1 mg, 82%). $^1$H NMR plus $^1$H-$^{13}$C HSQC (600.1 MHz, $CD_2Cl_2$): $\delta$ = 13.3 (d, $J_{P\text{-}H}$ = 24.0 Hz 1H, H1), 7.4 ppm (s, 1H, H6), 7.1 (s, 1H, H5), 7.6 (s, 1H, H10), 2.7 ppm (q, $J_{H\text{-}H}$ = 22.8 Hz 2H, H19), 1.32 ppm (t, $J_{H\text{-}H}$ = 7.5 Hz 3H, H20). $^{31}$P NMR (242.9 MHz, $CD_2Cl_2$): $\delta$= 14.0 (t, $J_{P\text{-}P}$ = 6.2 Hz C*P*$Ph_3$), –8.5 (dd, $J_{P\text{-}P}$ = 253.0 Hz, $J_{P\text{-}P}$ = 5.1 Hz, Os*P*$Ph_3$), –18.0 ppm (dd, $J_{P\text{-}P}$ = 253.5 Hz, $J_{P\text{-}P}$ = 6.7 Hz, Os*P*$Ph_3$). $^{13}$C NMR plus DEPT-135, $^1$H-$^{13}$C HSQC and $^1$H-$^{13}$C HMBC (150.9 MHz, $CD_2Cl_2$): $\delta$ = 234.1 (t, $J_{P\text{-}C}$ = 7.9 Hz, C7), 218.2 (t, $J_{P\text{-}C}$ = 4.3 Hz, C11), 207.8 (t, $J_{P\text{-}C}$ = 21.0 Hz, C1), 191.4 (dt, $J_{P\text{-}C}$ =20.5 Hz, $J_{P\text{-}C}$ = 5.6 Hz, C4), 161.7 (s, C6), 157.5 (s, C5), 144.1 (s, C9), 139.4 (s, C8), 120.3 (d, $J_{P\text{-}C}$ = 89.2 Hz, C2), 117.3 (s, C10), 109.6 (d, $J_{P\text{-}C}$ = 25.7 Hz, C3), 28.4 ppm (s, C19), 15.4 ppm (s, C20).14.0 ppm (s, C12). Elemental analysis calcd (%) for $C_{86}H_{69}Br_2OsP_3$: C 66.84, H 4.50; found: C 66.44, H 4.79. HRMS (ESI): m/z calcd for $[C_{86}H_{69}BrOsP_3]^+$, 1465.3394; found: 1465.3400.

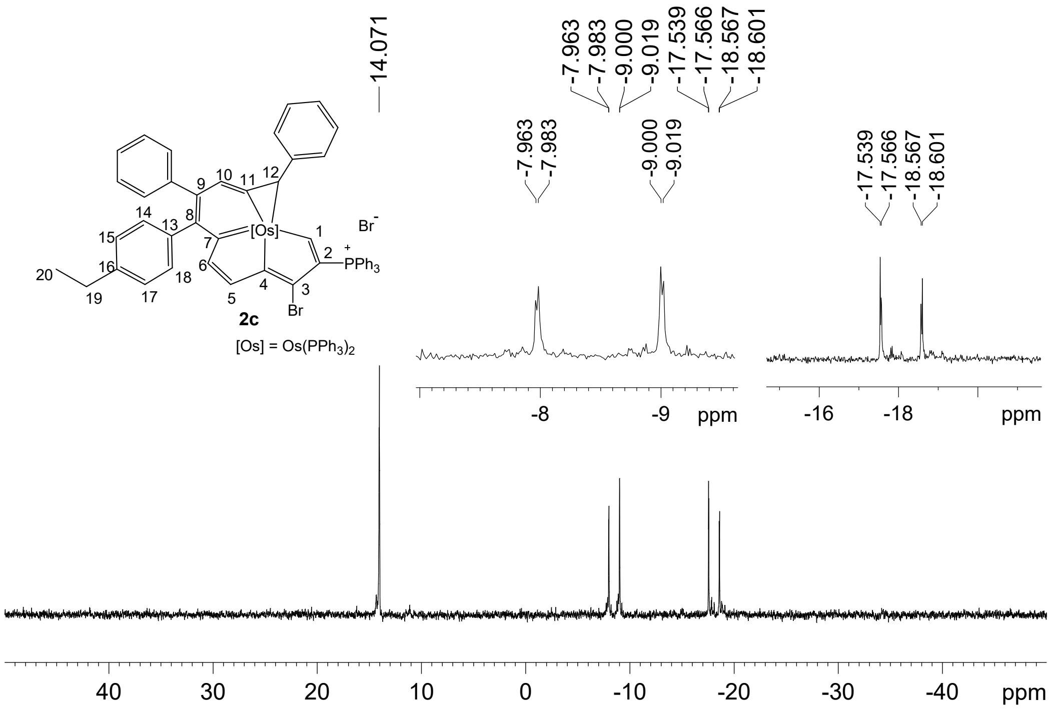


**Figure S9** The $^{31}P\{^{1}H\}$ NMR (242.9 MHz, $CD_2Cl_2$) spectrum for complex **2c**.

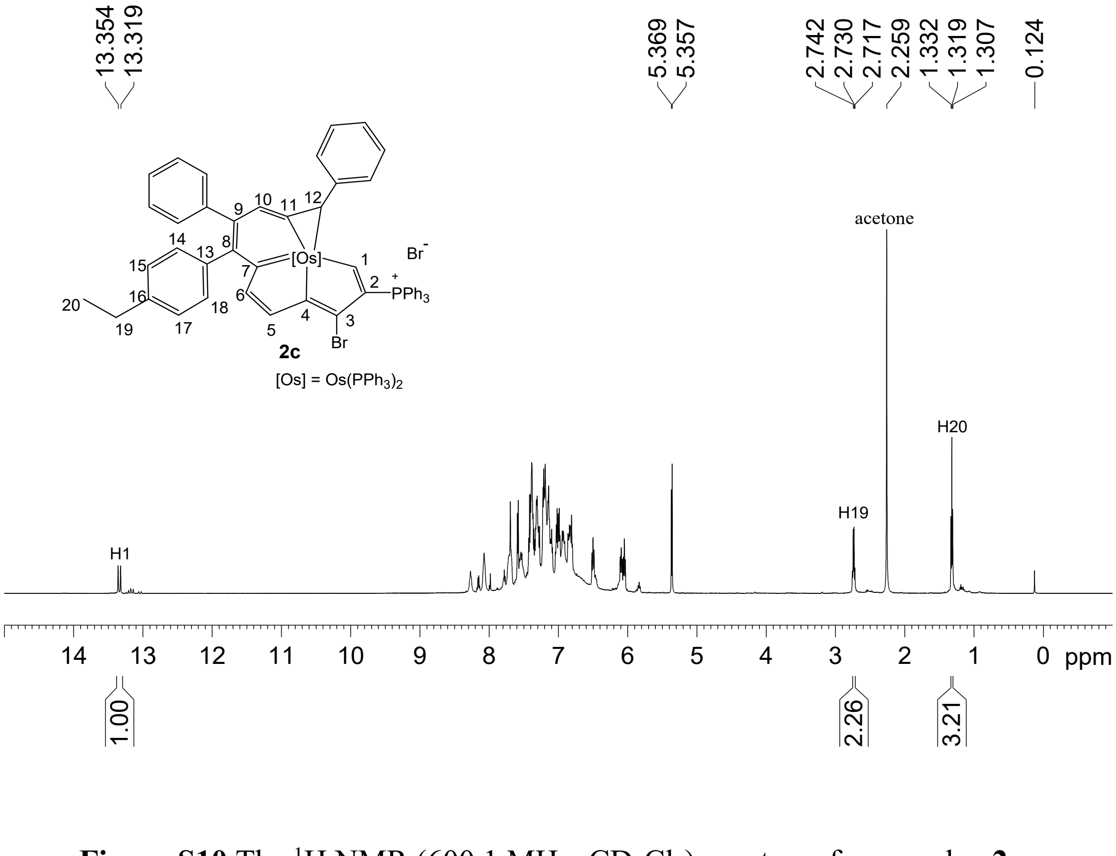


**Figure S10** The $^{1}H$ NMR (600.1 MHz, $CD_2Cl_2$) spectrum for complex **2c**.

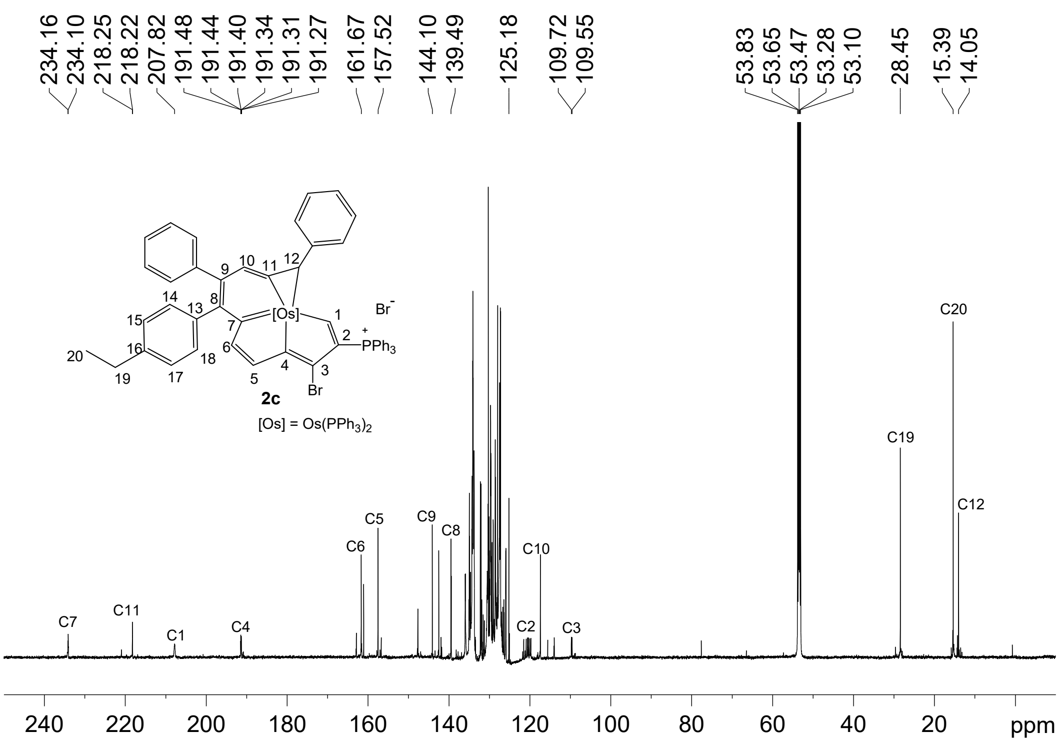


**Figure S11** The $^{13}$C NMR (150.9 MHz, $CD_2Cl_2$) spectrum for complex **2c**.

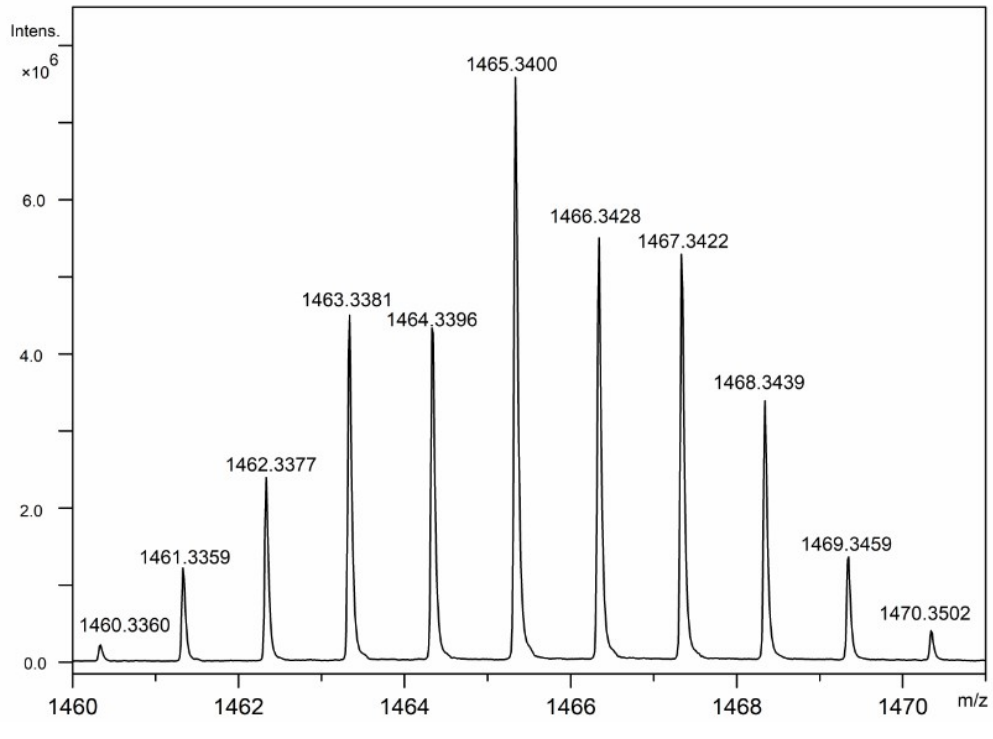


**Figure S12** Positive-ion ESI-MS spectrum of **2c** measured in methanol.

## Preparation of **2d**

**2d**: The synthetic procedure is similar to that for **2a**. **1** (91.60 mg, 0.06 mmol), (4-butylphenyl) boronic acid (37.38 mg 0.21 mmol), $Pd_2(dba)_3$ (5 mol%, 2.75 mg), $K_2CO_3$ (16.6 mg, 0.12 mmol) and 3 mL of 1,2-dichlorobenzene, afforded as yellow-green solid (**2d**, 72.2 mg, 87%). $^1$H NMR plus $^1$H-$^{13}$C HSQC (600.1 MHz, $CD_2Cl_2$): $\delta$ = 13.3 (d, $J_{P\text{-}H}$ = 20.8 Hz 1H, H1), 7.4 ppm (s, 1H, H6), 7.3 (s, 1H, H5), 7.6 (s, 1H, H10), 2.7 ppm (t, $J_{H\text{-}H}$ = 7.7 Hz 2H, H19), 1.7 ppm (m, 2H, H20), 1.4 ppm (m, 2H, H21), 1.0 ppm (t, $J_{H\text{-}H}$ = 7.2 Hz, 3H, H22). $^{31}$P NMR (242.9 MHz, $CD_2Cl_2$): $\delta$ = 14.0 (t, $J_{P\text{-}P}$ = 6.0 Hz C*P*Ph$_3$), –8.5 (dd, $J_{P\text{-}P}$ = 250.1 Hz, $J_{P\text{-}P}$ = 5.5 Hz, Os*P*Ph$_3$), –18.0 ppm (dd, $J_{P\text{-}P}$ = 250.1 Hz, $J_{P\text{-}P}$ = 8.1 Hz, Os*P*Ph$_3$). $^{13}$C NMR plus DEPT-135, $^1$H-$^{13}$C HSQC and $^1$H-$^{13}$C HMBC (150.9 MHz, $CD_2Cl_2$): $\delta$ = 234.0 (t, $J_{P\text{-}C}$ = 7.2 Hz, C7), 218.2 (t, $J_{P\text{-}C}$ = 4.3 Hz, C11), 207.7 (t, $J_{P\text{-}C}$ = 21.0 Hz, C1), 191.3 (dt, $J_{P\text{-}C}$ =20.5 Hz, $J_{P\text{-}C}$ = 5.5 Hz, C4), 161.7 (s, C6), 157.5 (s, C5), 144.0 (s, C9), 139.4 (s, C8), 120.3 (d, $J_{P\text{-}C}$ = 89.1 Hz, C2), 117.3 (s, C10), 109.6 (d, $J_{P\text{-}C}$ = 25.2 Hz, C3), 35.2 ppm (s, C19), 33.6 ppm (s, C20), 22.1 ppm (s, C21), 14.0 ppm (s, C12), 13.8 ppm (s, C22). Elemental analysis calcd (%) for $C_{88}H_{73}Br_2OsP_3$: C 67.17, H 4.68; found: C 67.36, H 4.52. HRMS (ESI): m/z calcd for $[C_{88}H_{73}BrOsP_3]^+$, 1493.3707; found: 1493.3709.

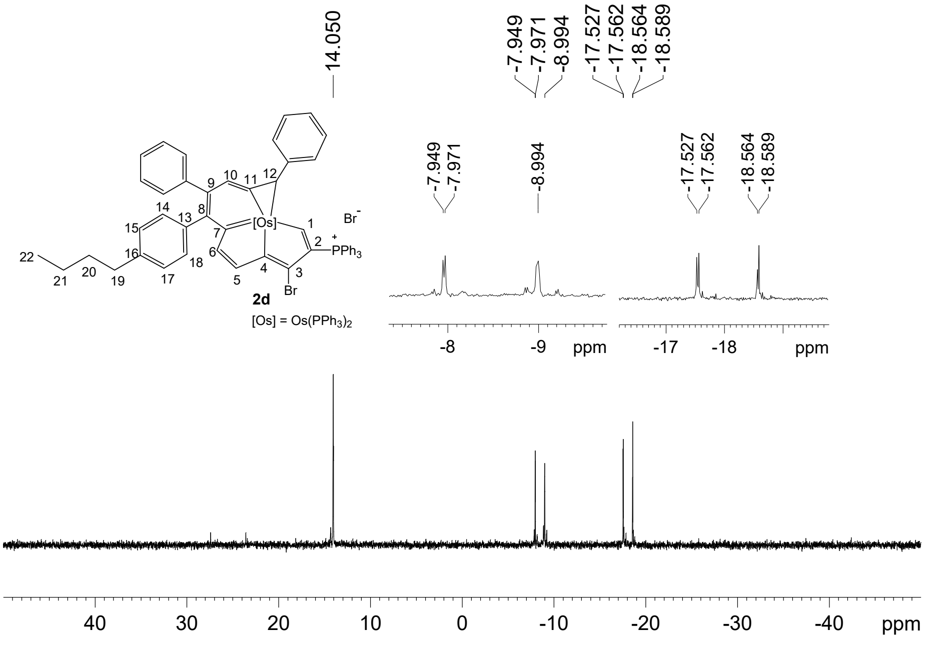


**Figure S13** The $^{31}$P{$^1$H} NMR (242.9 MHz, $CD_2Cl_2$) spectrum for complex **2d**.

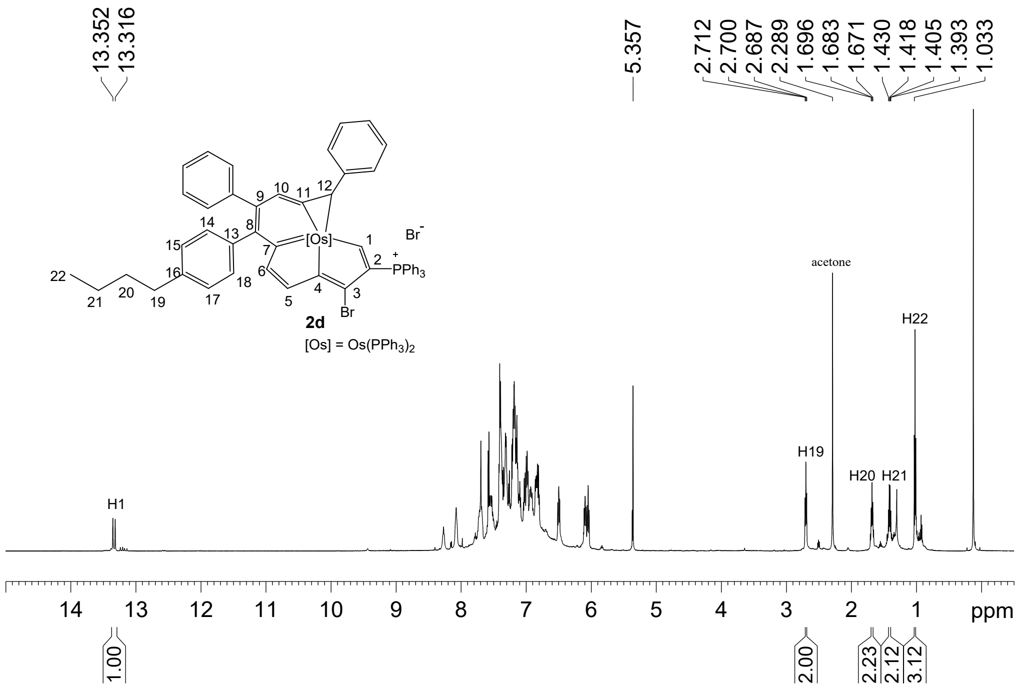


**Figure S14** The $^{1}H$ NMR (600.1 MHz, $CD_2Cl_2$) spectrum for complex **2d**.

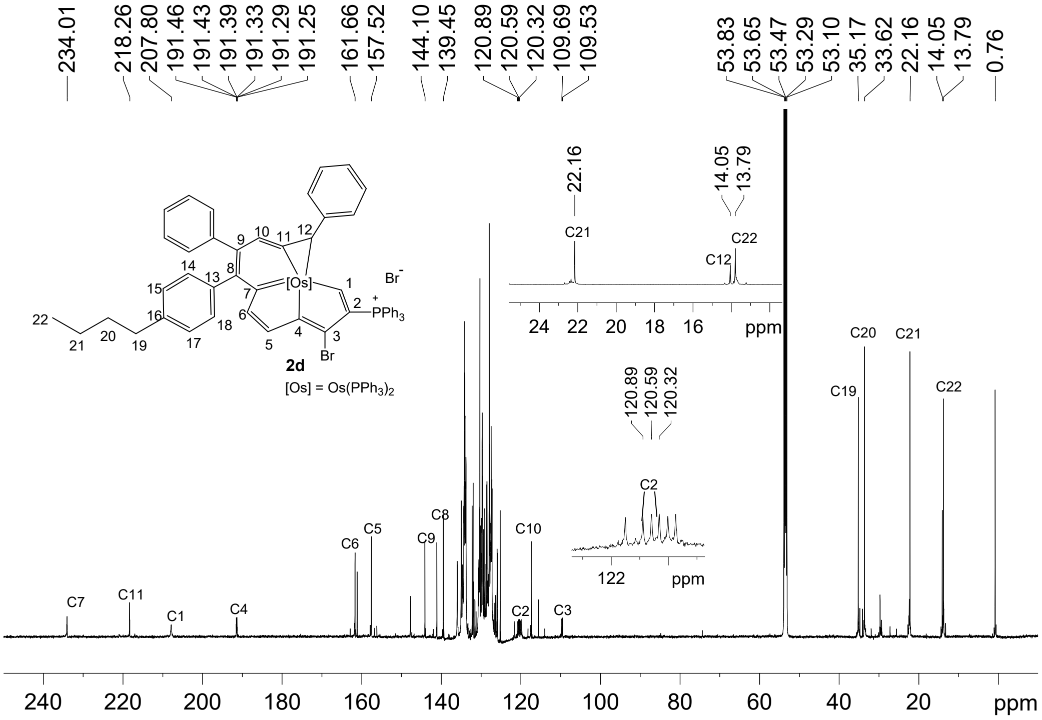


**Figure S15** The $^{13}C$ NMR (150.9 MHz, $CD_2Cl_2$) spectrum for complex **2d**.

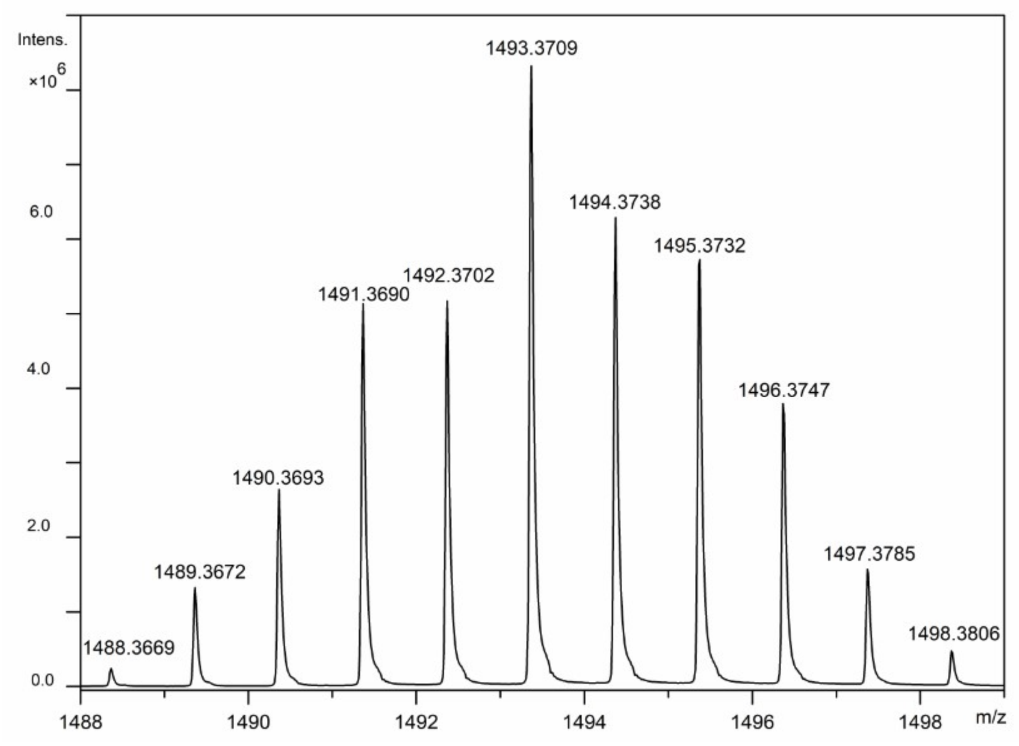


**Figure S16** Positive-ion ESI-MS spectrum of **2d** measured in methanol.

## Preparation of **2e**

**2e**: The synthetic procedure is similar to that for **2a**. **1** (91.6 mg, 0.06 mmol), (4-isopropylphenyl) boronic acid (34.44 mg 0.21 mmol), $Pd_2(dba)_3$ (5 mol%, 2.75 mg) and $K_2CO_3$ (16.6 mg, 0.12 mmol) and 3 mL of 1,2-dichlorobenzene, afforded as yellow-green solid (**2e**, 73.6 mg, 83%). $^1$H NMR plus $^1$H-$^{13}$C HSQC (600.1 MHz, $CD_2Cl_2$): $\delta$ = 13.3 (d, $J_{P\text{-}H}$ = 21.6 Hz 1H, H1), 7.4 ppm (s, 1H, H6), 7.2 (s, 1H, H5), 7.6 (s, 1H, H10), 2.9 ppm (m, 1H, H19), 1.3 ppm (d, $J_{H\text{-}H}$ = 6.9 Hz 6H, H20-21). $^{31}$P NMR (242.9 MHz, $CD_2Cl_2$): $\delta$= 14.0 (t, $J_{P\text{-}P}$ = 6.0 Hz C*P*Ph$_3$), –8.0 (dd, $J_{P\text{-}P}$ = 253.1 Hz, $J_{P\text{-}P}$ = 6.5 Hz, Os*P*Ph$_3$), –18.1 ppm (dd, $J_{P\text{-}P}$ = 252.1 Hz, $J_{P\text{-}P}$ = 5.6 Hz, Os*P*Ph$_3$). $^{13}$C NMR plus DEPT-135, $^1$H-$^{13}$C HSQC and $^1$H-$^{13}$C HMBC (150.9 MHz, $CD_2Cl_2$): $\delta$= 234.1 (t, $J_{P\text{-}C}$ = 8.5 Hz, C7), 218.2 (t, $J_{P\text{-}C}$ = 4.3 Hz, C11), 207.7 (t, $J_{P\text{-}C}$ = 21.0 Hz, C1), 191.3 (dt, $J_{P\text{-}C}$ =20.5 Hz, $J_{P\text{-}C}$ = 5.5 Hz, C4), 161.7 (s, C6), 157.5 (s, C5), 144.0 (s, C9), 139.4 (s, C8), 120.3 (d, $J_{P\text{-}C}$ = 89.1 Hz, C2), 117.3 (s, C10), 109.6 (d, $J_{P\text{-}C}$ = 26.2 Hz, C3), 33.7 ppm (s, C19), 23.8 ppm (s, C20-21).14.0 ppm (s, C12). Elemental analysis calcd (%) for $C_{87}H_{71}Br_2OsP_3$: C 67.01, H 4.59; found: C 67.40, H 4.52. HRMS (ESI): m/z calcd for $[C_{87}H_{71}BrOsP_3]^+$, 1479.3551; found: 1479.3552.

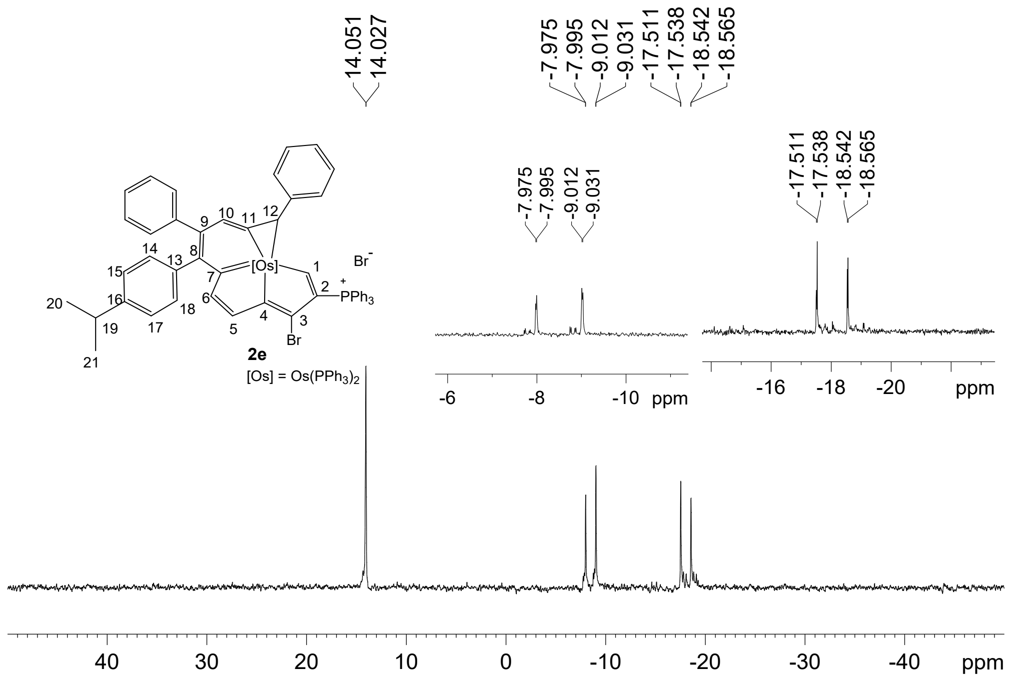


**Figure S17** The $^{31}P\{^{1}H\}$ NMR (242.9 MHz, $CD_2Cl_2$) spectrum for complex **2e.**

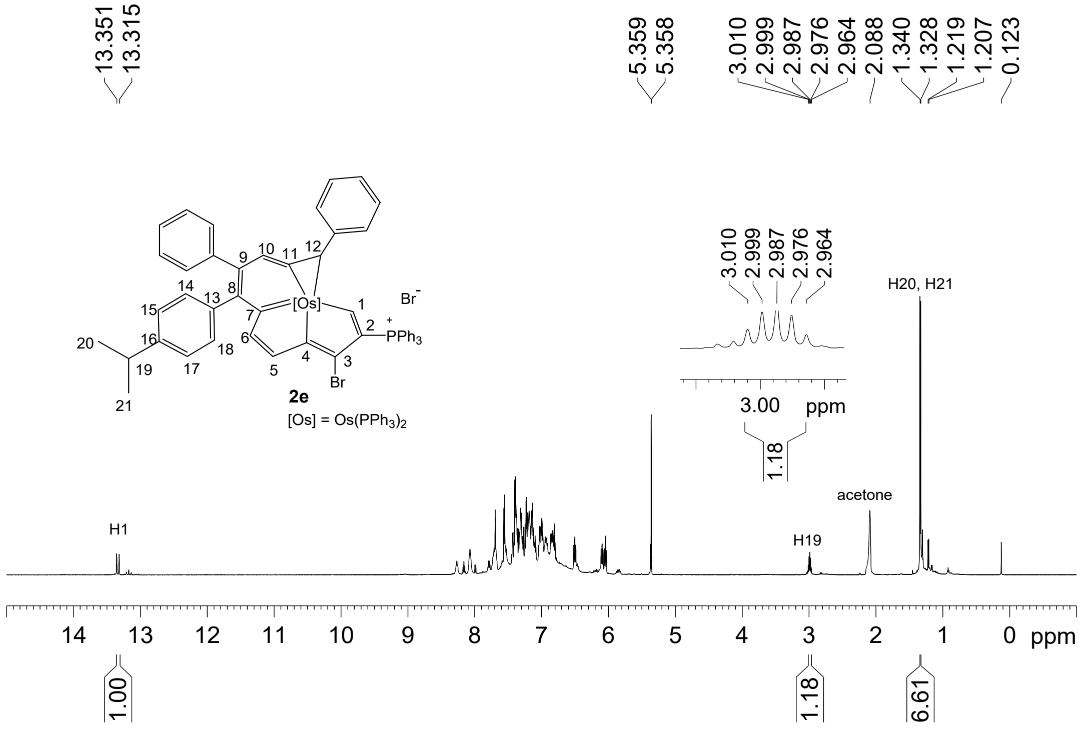


**Figure S18** The $^{1}H$ NMR (600.1 MHz, $CD_2Cl_2$) spectrum for complex **2e**.

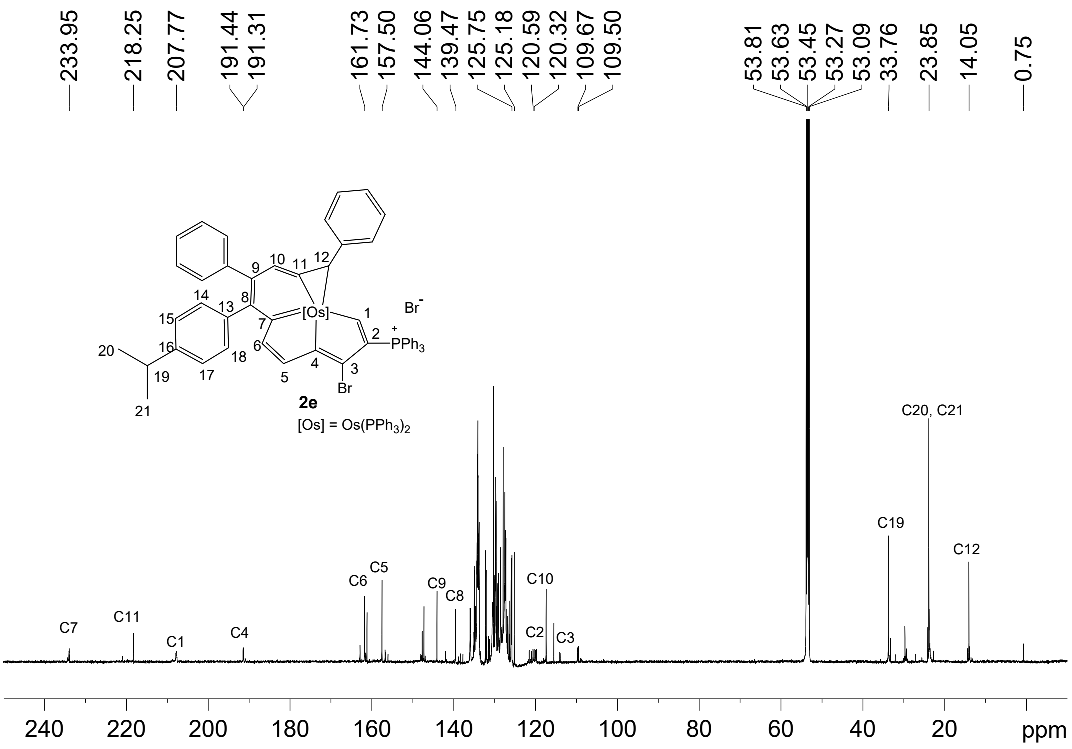


**Figure S19** The $^{13}C$ NMR (150.9 MHz, $CD_2Cl_2$) spectrum for complex **2e**.

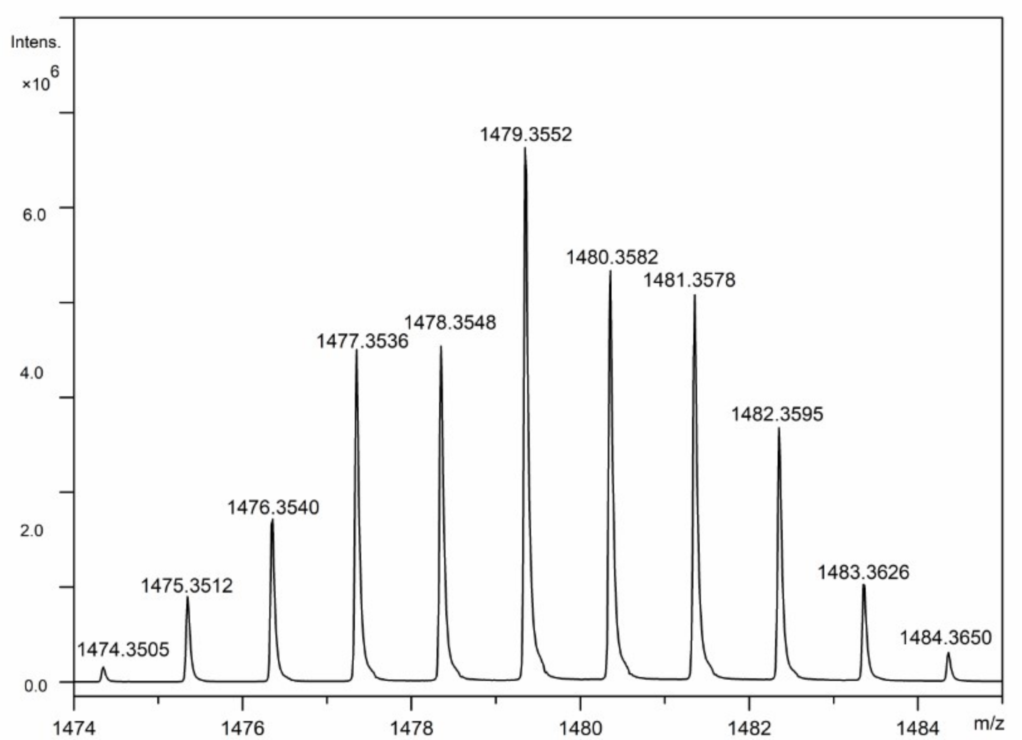


**Figure S20** Positive-ion ESI-MS spectrum of **2e** measured in methanol.

Preparation of **2f**

**2f**: The synthetic procedure is similar to that for **2a**. **1** (91.6 mg, 0.06 mmol), (4-(*tert*-

butyl)phenyl) boronic acid (37.38 mg 0.21 mmol), $Pd_2(dba)_3$ (5 mol%, 2.75 mg), $K_2CO_3$ (16.6 mg, 0.12 mmol) and 3 mL of 1,2-dichlorobenzene, afforded as yellow-green solid (**2f**, 76.2 mg, 85%). $^1$H NMR plus $^1$H-$^{13}$C HSQC (600.1 MHz, $CD_2Cl_2$): $\delta$ = 13.3 (d, $J_{P\text{-}H}$ = 21.4 Hz 1H, H1), 7.4 ppm (s, 1H, H6), 7.2 (s, 1H, H5), 7.6 (s, 1H, H10), 1.4 ppm (s, 9H, H20-22). $^{31}$P NMR (242.9 MHz, $CD_2Cl_2$): $\delta$ = 14.0 (t, $J_{P\text{-}P}$ = 5.1 Hz C*P*Ph$_3$), –8.5 (dd, $J_{P\text{-}P}$ = 250.1 Hz, $J_{P\text{-}P}$ = 5.9 Hz, Os*P*Ph$_3$), –18.0 ppm (dd, $J_{P\text{-}P}$ = 250.1 Hz, $J_{P\text{-}P}$ = 5.8 Hz, Os*P*Ph$_3$). $^{13}$C NMR plus DEPT-135, $^1$H-$^{13}$C HSQC and $^1$H-$^{13}$C HMBC (150.9 MHz, $CD_2Cl_2$): $\delta$ = 233.8 (t, $J_{P\text{-}C}$ = 8.7 Hz, C7), 218.2 (t, $J_{P\text{-}C}$ = 4.7 Hz, C11), 207.7 (t, $J_{P\text{-}C}$ = 21.6 Hz, C1), 191.4 (dt, $J_{P\text{-}C}$ =20.1 Hz, $J_{P\text{-}C}$ = 5.2 Hz, C4), 161.7 (s, C6), 157.5 (s, C5), 144.0 (s, C9), 139.4 (s, C8), 120.3 (d, $J_{P\text{-}C}$ = 86.2 Hz, C2), 117.3 (s, C10), 109.6 (d, $J_{P\text{-}C}$ = 26.2 Hz, C3), 34.3 ppm (s, C19), 31.2 ppm (s, C20-22), 14.0 ppm (s, C12). Elemental analysis calcd (%) for $C_{88}H_{73}Br_2OsP_3$: C 67.17, H 4.68; found: C 67.26, H 4.49. HRMS (ESI): m/z calcd for $[C_{88}H_{73}BrOsP_3]^+$, 1493.3707; found: 1493.3711.

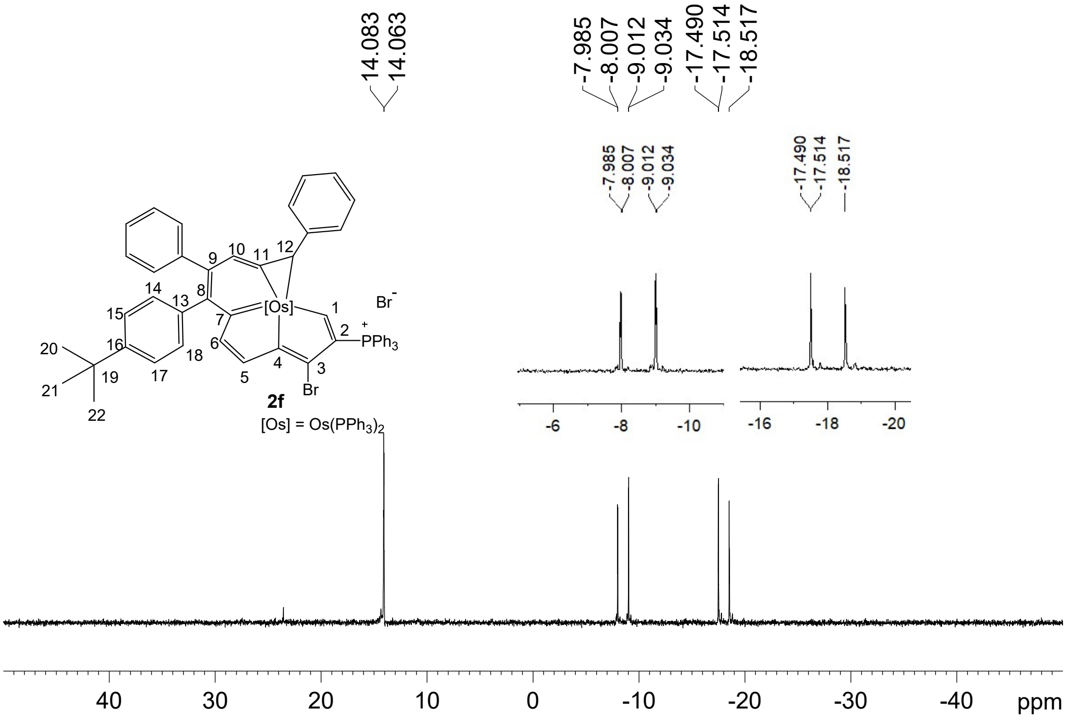


**Figure S21** The $^{31}$P{$^1$H} NMR (242.9 MHz, $CD_2Cl_2$) spectrum for complex **2f**.

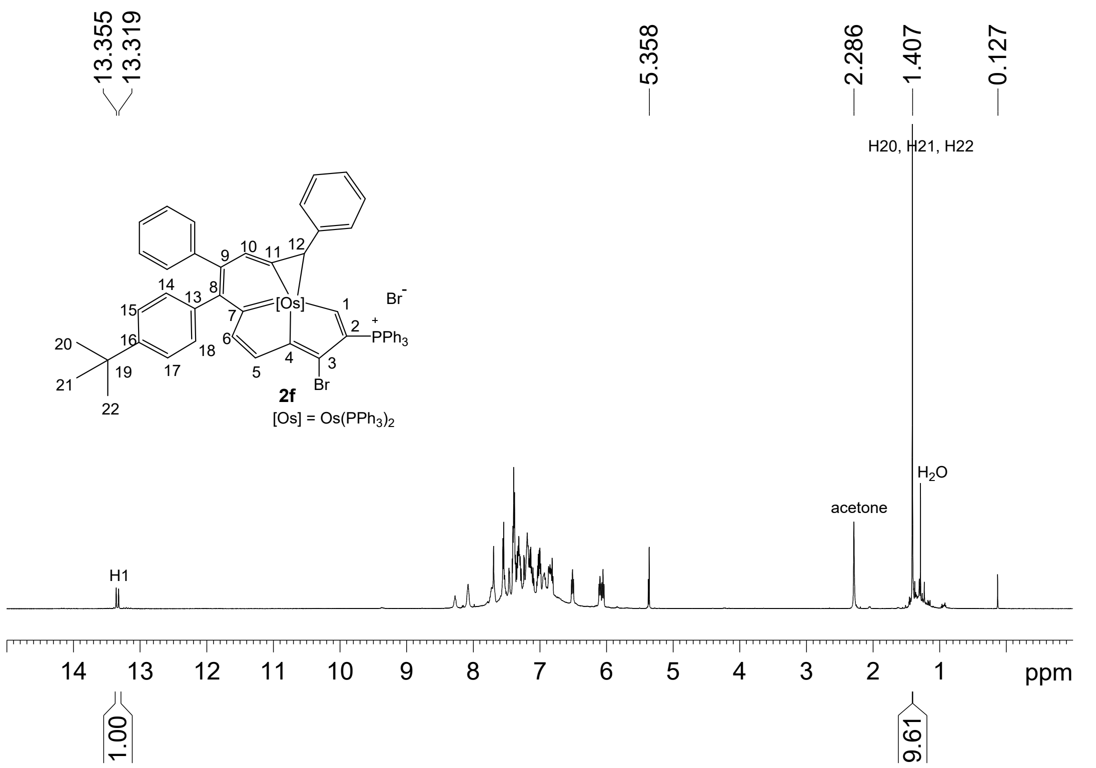


**Figure S22** The $^{1}H$ NMR (600.1 MHz, $CD_2Cl_2$) spectrum for complex **2f**.

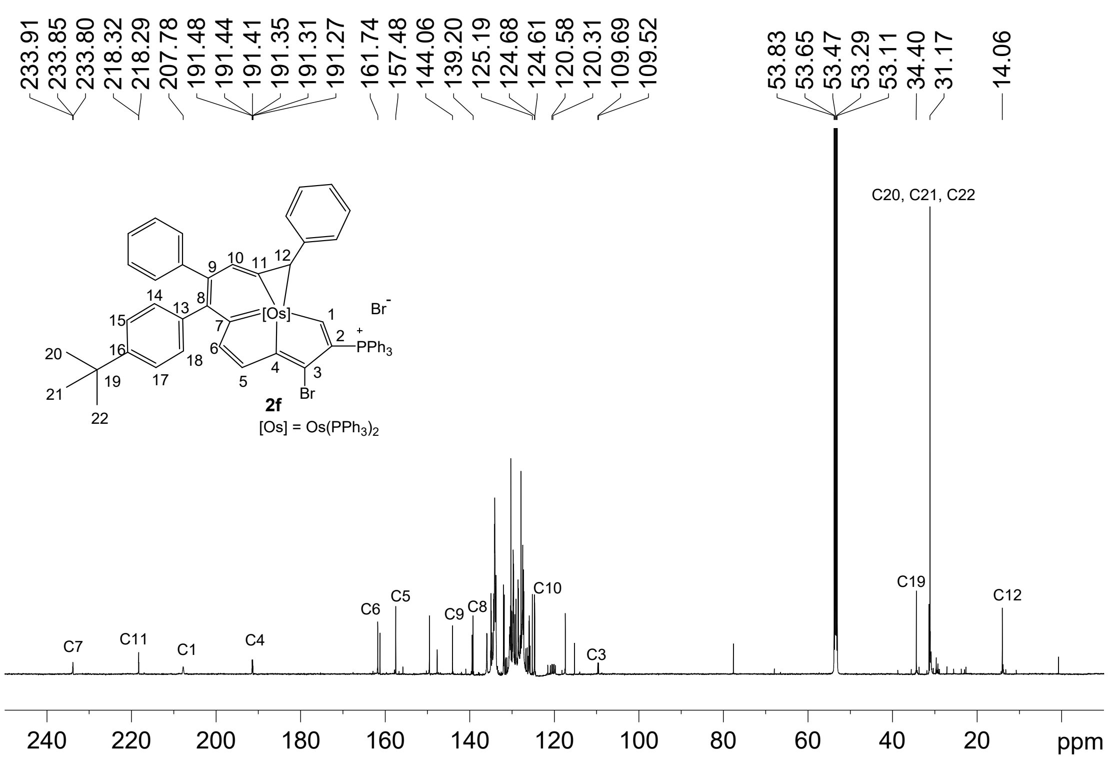


**Figure S23** The $^{13}C$ NMR (150.9 MHz, $CD_2Cl_2$) spectrum for complex **2f**.

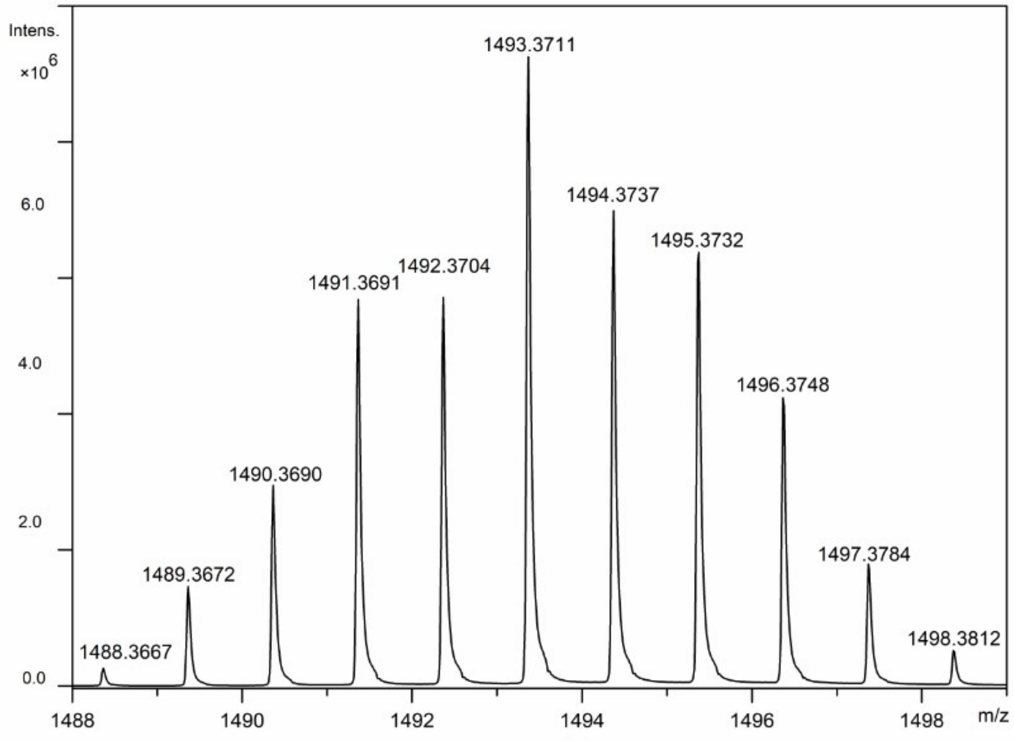


**Figure S24** Positive-ion ESI-MS spectrum of **2f** measured in methanol.

Preparation of **2g**

**2g**: The synthetic procedure is similar to that for **2a**. **1** (91.6 mg, 0.06 mmol), (4-methoxyphenyl)boronic acid (31.90 mg 0.21 mmol), $Pd_2(dba)_3$ (5 mol%, 2.75 mg), $K_2CO_3$ (16.6 mg, 0.12 mmol) and 3 mL of 1,2-dichlorobenzene, afforded as yellow-green solid (**2g**, 80.9 mg, 92%). $^1$H NMR plus $^1$H-$^{13}$C HSQC (500.2 MHz, $CD_2Cl_2$): $\delta$ = 13.3 (d, $J_{P\text{-}H}$ = 21.9 Hz 1H, H1), 7.7 (s, 1H, H10), 7.4 ppm (s,1H, H6) 7.2 (s,1H, H5), 3.9 (s,3H, H17) $^{31}$P NMR (202.5 MHz, $CD_2Cl_2$): $\delta$= 14.1 (t, $J_{P\text{-}P}$ = 5.8 Hz C*P*Ph$_3$), –8.9 (dd, $J_{P\text{-}P}$ = 248.9 Hz, $J_{P\text{-}P}$ =5.3 Hz, Os*P*Ph$_3$), –17.6 ppm (dd, $J_{P\text{-}P}$ = 248.9 Hz, $J_{P\text{-}P}$ = 5.3 Hz, Os*P*Ph$_3$). $^{13}$C NMR plus DEPT-135, $^1$H-$^{13}$C HSQC and $^1$H-$^{13}$C HMBC (125.8 MHz, $CD_2Cl_2$): $\delta$= 234.5 (t, $J_{P\text{-}C}$ = 8.4 Hz, C7), 218.1 (t, $J_{P\text{-}C}$ = 4.3 Hz, C11), 207.8 (t, $J_{P\text{-}C}$ = 12 Hz, C1), 191.3 (dt, $J_{P\text{-}C}$ =18.5 Hz, $J_{P\text{-}C}$ = 4.7 Hz, C4), 161.6 (s, C6), 157.6 (s, C5), 144.2 (s, C9), 138.9 (s, C8), 120.3 (d, $J_{P\text{-}C}$ = 88.2 Hz, C2), 117.5 (s, C10), 109.5 (d, $J_{P\text{-}C}$ = 26.3 Hz, C3), 55.2 ppm (s, C19), 14.1 ppm (s, C12). Elemental analysis calcd (%) for $C_{85}H_{67}Br_2OOsP_3$: C 65.98, H 4.36; found: C 65.78, H 4.11. HRMS (ESI): m/z calcd

for $[C_{85}H_{67}BrOOsP_3]^+$: 1467.3186; found: 1467.3187.

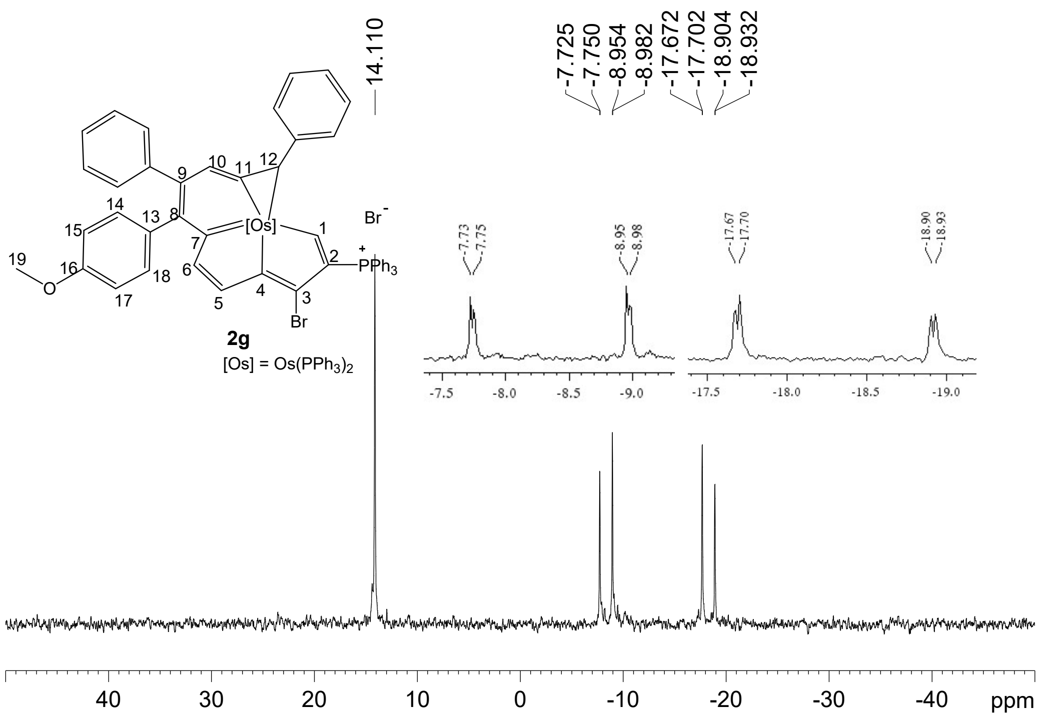

**Figure S25** The $^{31}P\{^1H\}$ NMR (202.5 MHz, $CD_2Cl_2$) spectrum for complex **2g**.

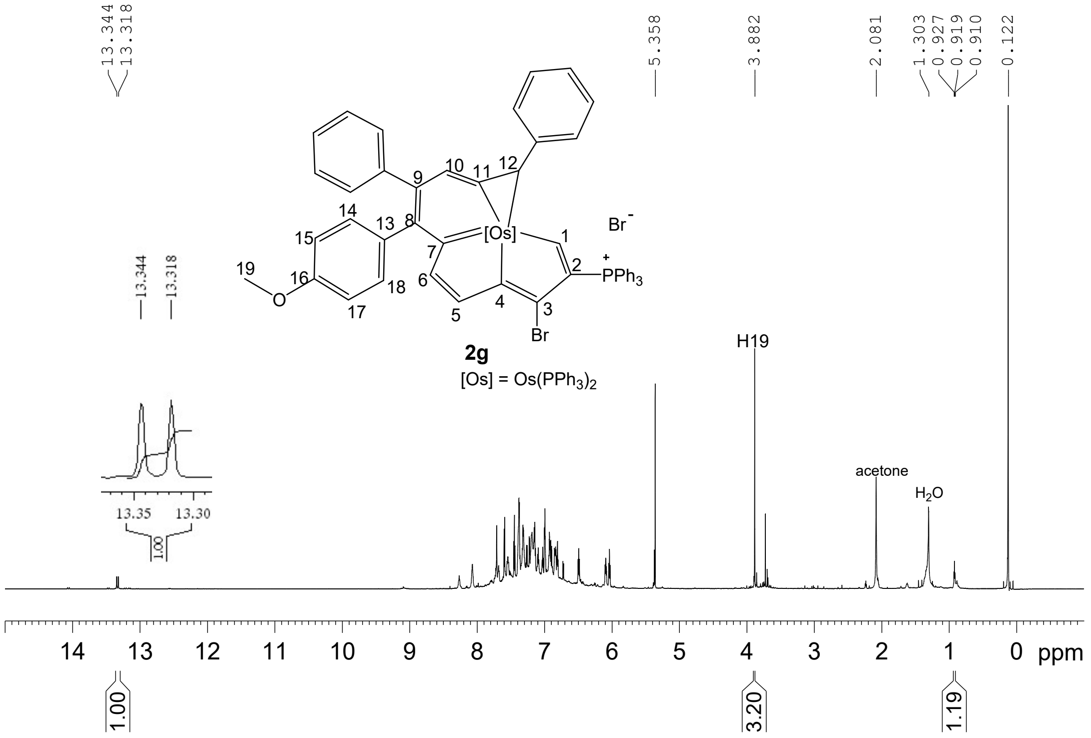

**Figure S26** The $^1H$ NMR (500.2 MHz, $CD_2Cl_2$) spectrum for complex **2g**.

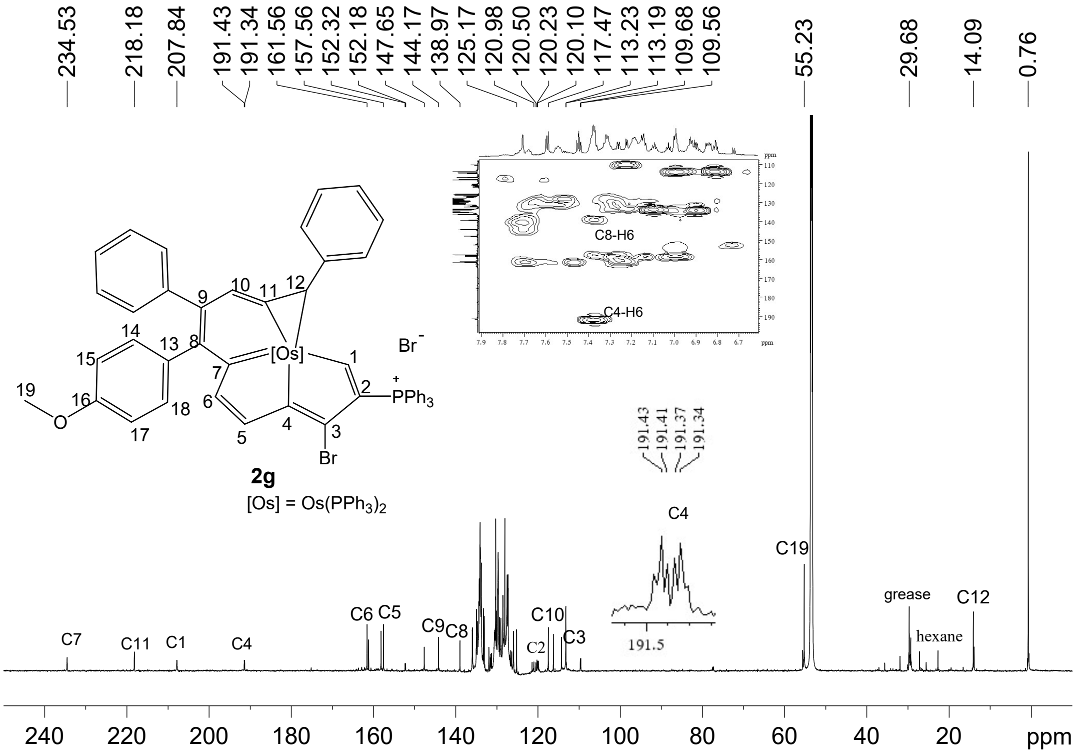


**Figure S27** The $^{13}C$ NMR (125.8 MHz, $CD_2Cl_2$) spectrum for complex **2g**.

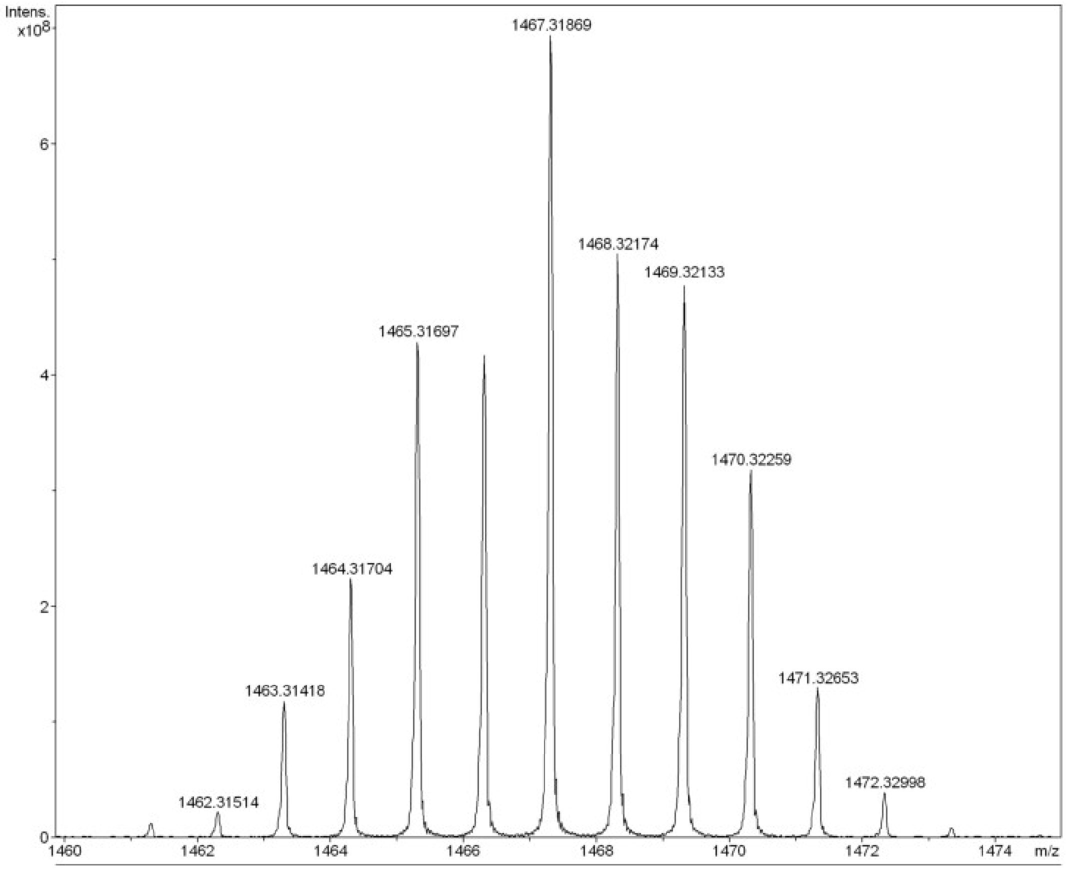


**Figure S28** Positive-ion ESI-MS spectrum of **2g** measured in methanol.

Preparation of **2h**

**2h**: The synthetic procedure is similar to that for **2a**. **1** (91.6 mg, 0.06 mmol), (4-(diphenylamino)phenyl) boronic acid (60.72 mg 0.21 mmol), $Pd_2(dba)_3$ (5 mol%, 2.75 mg), $K_2CO_3$ (16.6 mg, 0.12 mmol) and 3 mL of 1,2-dichlorobenzene, afforded as yellow-green solid (**2h**, 84.6 mg, 88%). $^1$H NMR plus $^1$H-$^{13}$C HSQC (500.2 MHz, $CD_2Cl_2$): $\delta$ = 13.4 (d, $J_{P\text{-}H}$ = 21.6 Hz 1H, H1), 7.7 (s, 1H, H10), 7.6 ppm (s,1H, H6) 7.3 (s,1H, H5). $^{31}$P NMR (202.5 MHz, $CD_2Cl_2$): $\delta$ = 14.1 (t, $J_{P\text{-}P}$ = 6.8 Hz C*P*Ph$_3$), –9.2 (dd, $J_{P\text{-}P}$ = 250.7 Hz, $J_{P\text{-}P}$ =5.7 Hz, Os*P*Ph$_3$), –17.4 ppm (dd, $J_{P\text{-}P}$ = 250.7 Hz, $J_{P\text{-}P}$ = 5.7 Hz, Os*P*Ph$_3$). $^{13}$C NMR plus DEPT-135, $^1$H-$^{13}$C HSQC and $^1$H-$^{13}$C HMBC (125.8 MHz, $CD_2Cl_2$): $\delta$ = 233.6 (t, $J_{P\text{-}C}$ = 7.1 Hz, C7), 218.6 (t, $J_{P\text{-}C}$ = 4.2 Hz, C11), 207.7 (t, $J_{P\text{-}C}$ = 12 Hz, C1), 191.3 (dt, $J_{P\text{-}C}$ =19.9 Hz, $J_{P\text{-}C}$ = 6.2 Hz, C4), 161.5 (s, C6), 157.7 (s, C5), 146.1 (s, C16),144.2 (s, C9), 138.1 (s, C8), 117.4 (s, C10), 109.8 (d, $J_{P\text{-}C}$ = 25.1 Hz, C3), 14.3 ppm (s, C12). Elemental analysis calcd (%) for $C_{96}H_{74}Br_2NOsP_3$: C 68.45, H 4.43, N 0.83; found: C 68.50, H 4.84, N 0.79. HRMS (ESI): m/z calcd for $[C_{96}H_{74}BrNOsP_3]^+$, 1604.3825; found: 1604.3827.

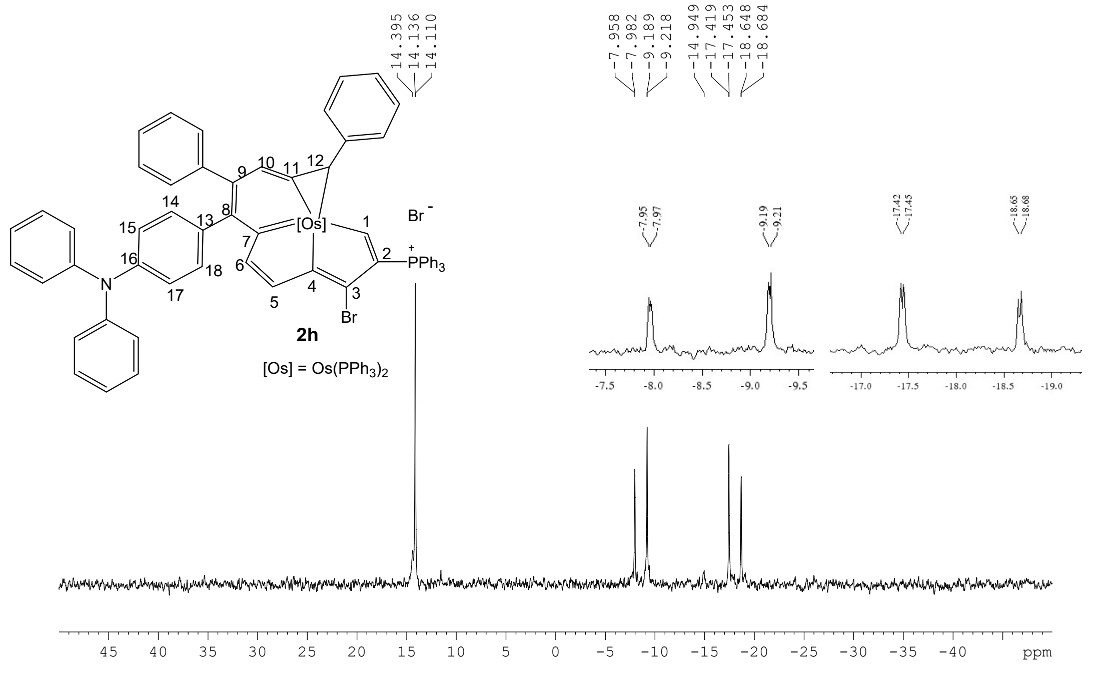


**Figure S29** The $^{31}$P{$^1$H} NMR (202.5 MHz, $CD_2Cl_2$) spectrum for complex **2h**.

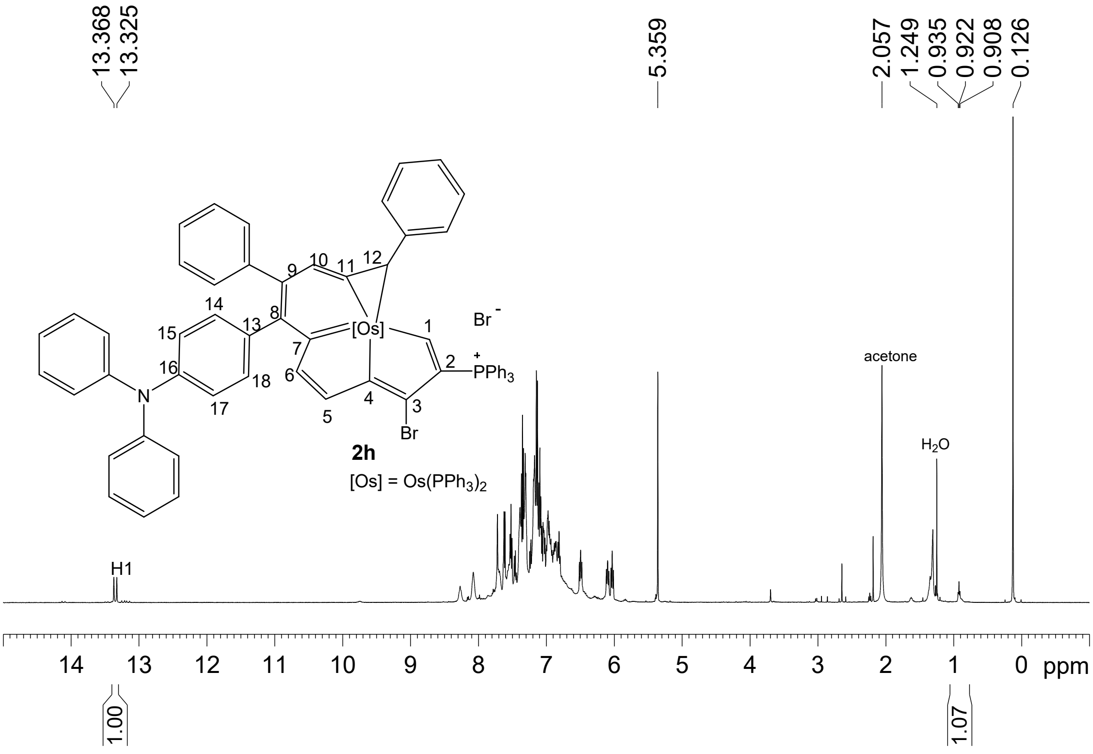


**Figure S30** The $^{1}$H NMR (500.2 MHz, $CD_2Cl_2$) spectrum for complex **2h**.

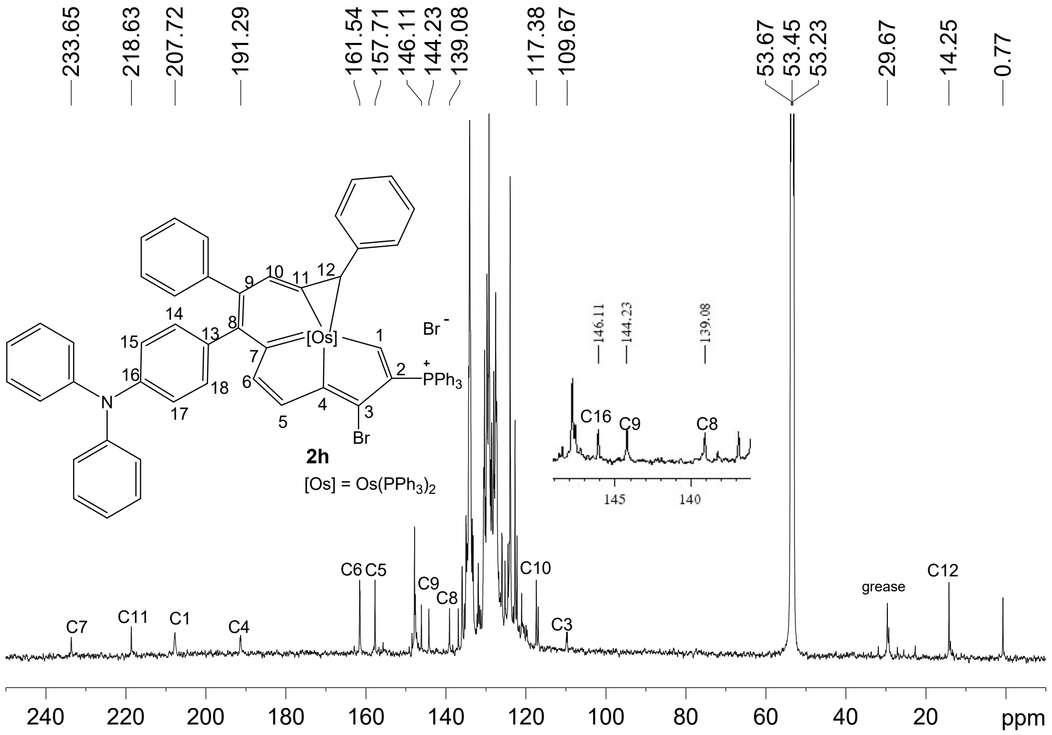


**Figure S31** The $^{13}$C NMR (125.8 MHz, $CD_2Cl_2$) spectrum for complex **2h**.

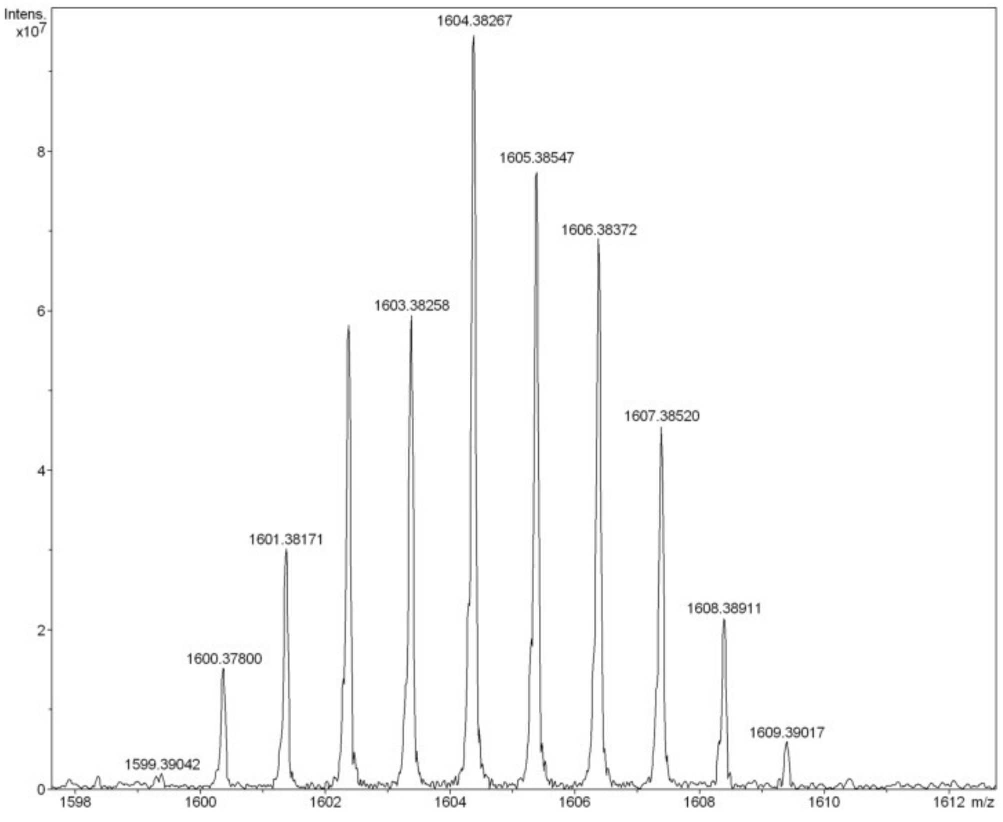


**Figure S32** Positive-ion ESI-MS spectrum of **2h** measured in methanol.

Preparation of **2i**

**2i**: The synthetic procedure is similar to that for **2a**. **1** (91.6 mg, 0.06 mmol), (4-fluorophenyl) boronic acid (29.38 mg 0.21 mmol), $Pd_2(dba)_3$ (5 mol%, 2.75 mg), $K_2CO_3$ (16.6 mg, 0.12 mmol) and 3 mL of 1,2-dichlorobenzene, afforded as yellow-green solid (**2i**, 75.9 mg, 87%). $^1$H NMR plus $^1$H-$^{13}$C HSQC (500.2 MHz, $CD_2Cl_2$): $\delta$ = 13.3 (d, $J_{P\text{-}H}$ = 21.1 Hz 1H, H1), 7.7 (s, 1H, H10), 7.3 ppm (s,1H, H6) 7.2 (s,1H, H5), 1.36 (s,1H, H12) $^{31}$P NMR (202.5 MHz, $CD_2Cl_2$): $\delta$= 14.2 (t, $J_{P\text{-}P}$ = 5.6 Hz C*P*Ph$_3$), –9.3 (dd, $J_{P\text{-}P}$ = 251.7 Hz, $J_{P\text{-}P}$ =5.7 Hz, Os*P*Ph$_3$), –17.5 ppm (dd, $J_{P\text{-}P}$ = 251.7 Hz, $J_{P\text{-}P}$ = 5.7 Hz, Os*P*Ph$_3$). $^{13}$C NMR plus DEPT-135, $^1$H-$^{13}$C HSQC and $^1$H-$^{13}$C HMBC (125.8 MHz, $CD_2Cl_2$): $\delta$= 233.6 (t, $J_{P\text{-}C}$ = 9.5 Hz, C7), 218.8 (t, $J_{P\text{-}C}$ = 4.7 Hz, C11), 207.9 (t, $J_{P\text{-}C}$ = 12.3 Hz, C1), 191.3 (dt, $J_{P\text{-}C}$ =21.1 Hz, $J_{P\text{-}C}$ = 6.1 Hz, C4), 161.2 (s, C6), 157.9 (s, C5), 143.8 (s, C9), 138.4 (s, C8), 117.3 (s, C10), 110.1 (d, $J_{P\text{-}C}$ = 25.1 Hz, C3), 14.4 ppm (s, C12). Elemental analysis calcd (%) for $C_{84}H_{64}Br_2FOsP_3$: C 65.71, H 4.20; found: C 65.83, H 4.09. HRMS (ESI): m/z calcd for $[C_{84}H_{64}BrFOsP_3]^+$: 1455.2986;

found: 1455.2968.

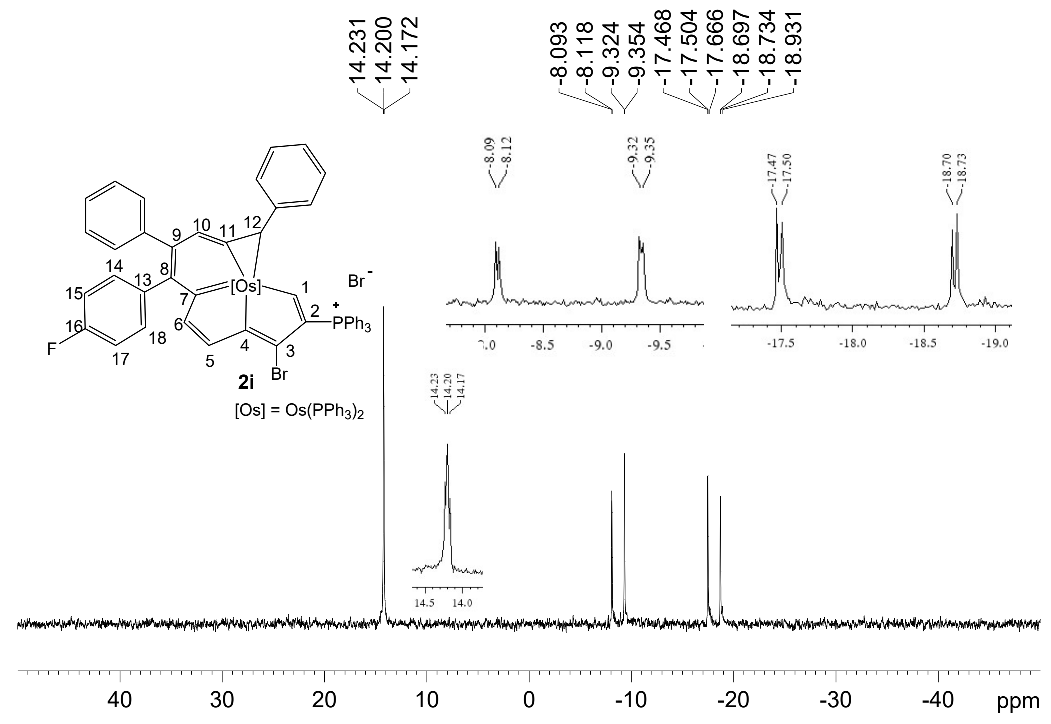


**Figure S33** The $^{31}P\{^{1}H\}$ NMR (202.5 MHz, $CD_2Cl_2$) spectrum for complex **2i**.

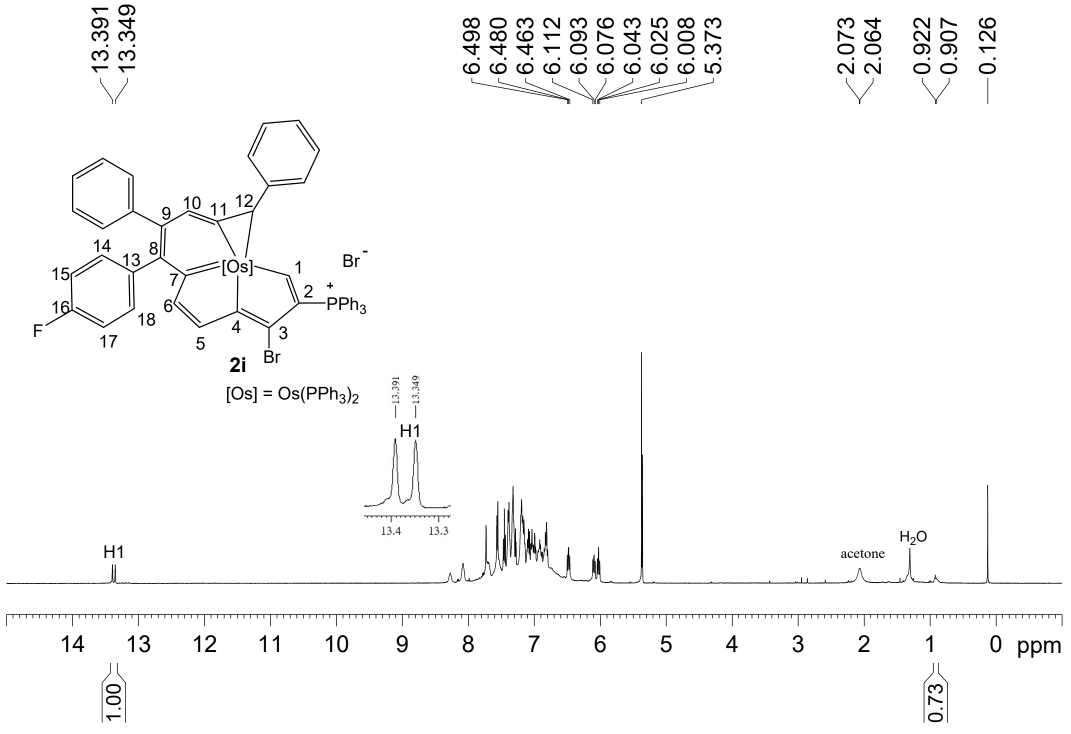


**Figure S34** The $^{1}H$ NMR (500.2 MHz, $CD_2Cl_2$) spectrum for complex **2i**.

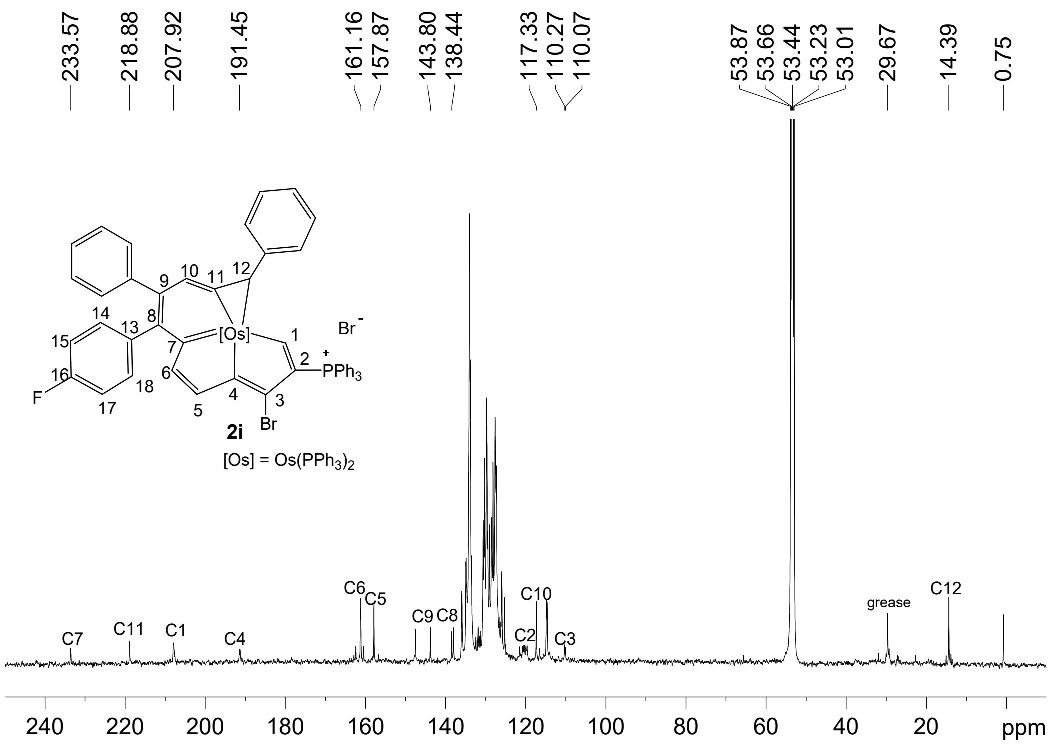


**Figure S35** The $^{13}$C NMR (125.8 MHz, CD2Cl2) spectrum for complex **2i**.

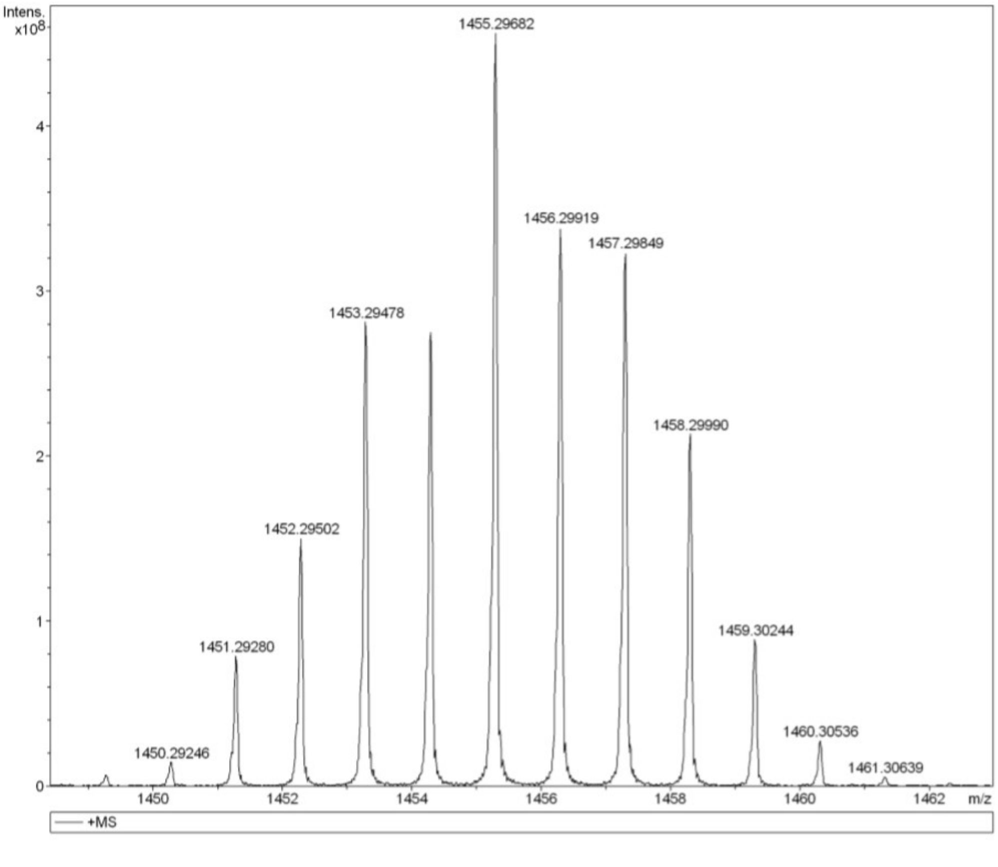


**Figure S36** Positive-ion ESI-MS spectrum of **2i** measured in methanol.

Preparation of **2j**

**2j**: The synthetic procedure is similar to that for **2a**. **1** (91.6 mg, 0.06 mmol), naphthalen-2-ylboronic acid (36.12 mg 0.21 mmol), $Pd_2(dba)_3$ (5 mol%, 2.75 mg), $K_2CO_3$ (16.6 mg, 0.12 mmol) and 3 mL of 1,2-dichlorobenzene, afforded as yellow-green solid (**2j**, 72.3 mg, 81%). $^1$H NMR plus $^1$H-$^{13}$C HSQC (600.1 MHz, $CD_2Cl_2$): $\delta$ = 13.4 (d, $J_{P\text{-}H}$ = 20.8 Hz 1H, H1), 7.3 ppm (s, 1H, H6), 7.2 (s, 1H, H5), 7.6 (s, 1H, H10). $^{31}$P NMR (242.9 MHz, $CD_2Cl_2$): $\delta$= 14.2 (d, $J_{P\text{-}P}$ = 10.2 Hz C*P*Ph$_3$), –8.6 (ddd, $J_{P\text{-}P}$ = 248.1 Hz, $J_{P\text{-}P}$ = 5.9 Hz, Os*P*Ph$_3$), –18.5 ppm (ddd, $J_{P\text{-}P}$ = 249.8 Hz, $J_{P\text{-}P}$ = 6.1 Hz, Os*P*Ph$_3$). $^{13}$C NMR plus DEPT-135, $^1$H-$^{13}$C HSQC and $^1$H-$^{13}$C HMBC (150.9 MHz, $CD_2Cl_2$): $\delta$= 233.8 (dt, $J_{P\text{-}C}$ = 32.0 Hz, C7), 218.2 (t, $J_{P\text{-}C}$ = 9.2 Hz, C11), 207.7 (t, $J_{P\text{-}C}$ = 6.8 Hz, C1), 191.4 (dt, $J_{P\text{-}C}$ =19.5 Hz, $J_{P\text{-}C}$ = 5.2 Hz, C4), 161.6 (s, C6), 157.8 (s, C5), 143.8 (s, C9), 138.9 (s, C8), 120.3 (d, $J_{P\text{-}C}$ = 91.2 Hz, C2), 117.4 (s, C10), 109.9 (d, $J_{P\text{-}C}$ = 25.5 Hz, C3) 14.4 (s, C12). Elemental analysis calcd (%) for $C_{88}H_{67}Br_2OsP_3$: C 67.43, H 4.31; found: C 67.25, H 4.58. HRMS (ESI): m/z calcd for $[C_{88}H_{67}BrOsP_3]^+$: 1487.3238; found: 1487.3250.

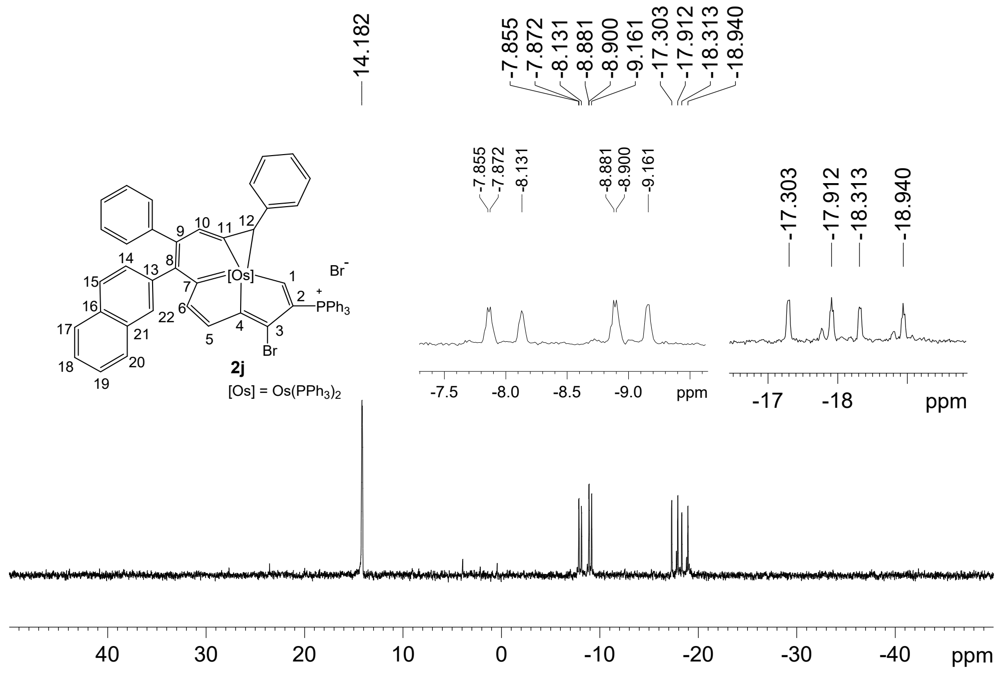


**Figure S37** The $^{31}$P{$^1$H} NMR (242.9 MHz, $CD_2Cl_2$) spectrum for complex **2j**.

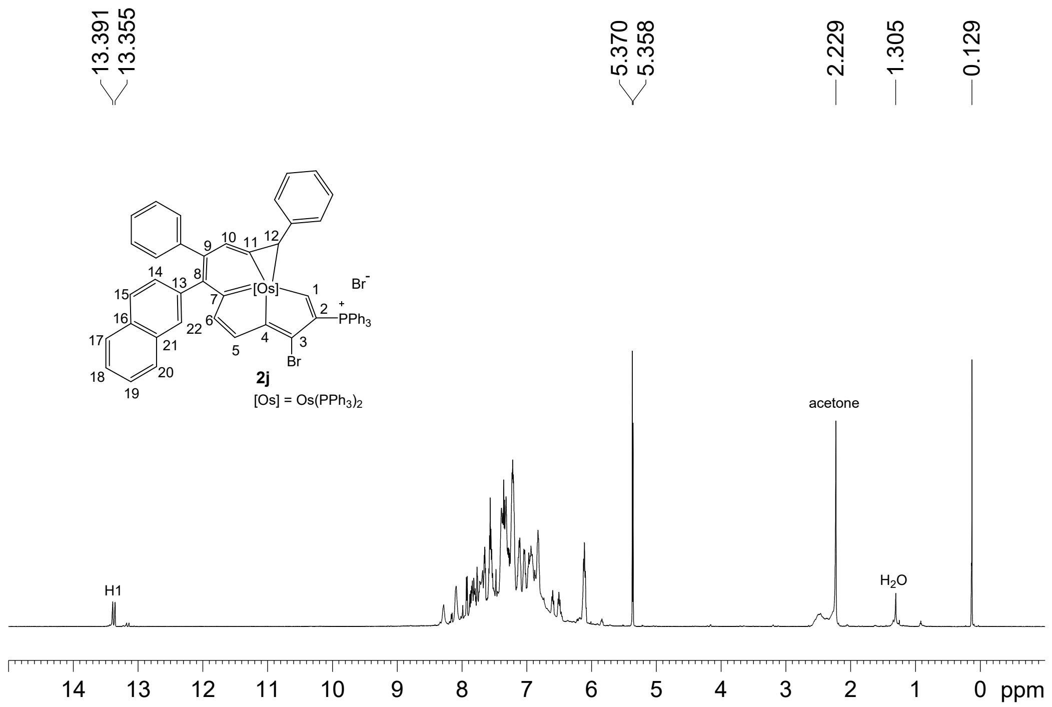


**Figure S38** The $^{1}H$ NMR (600.1 MHz, $CD_2Cl_2$) spectrum for complex **2j**.

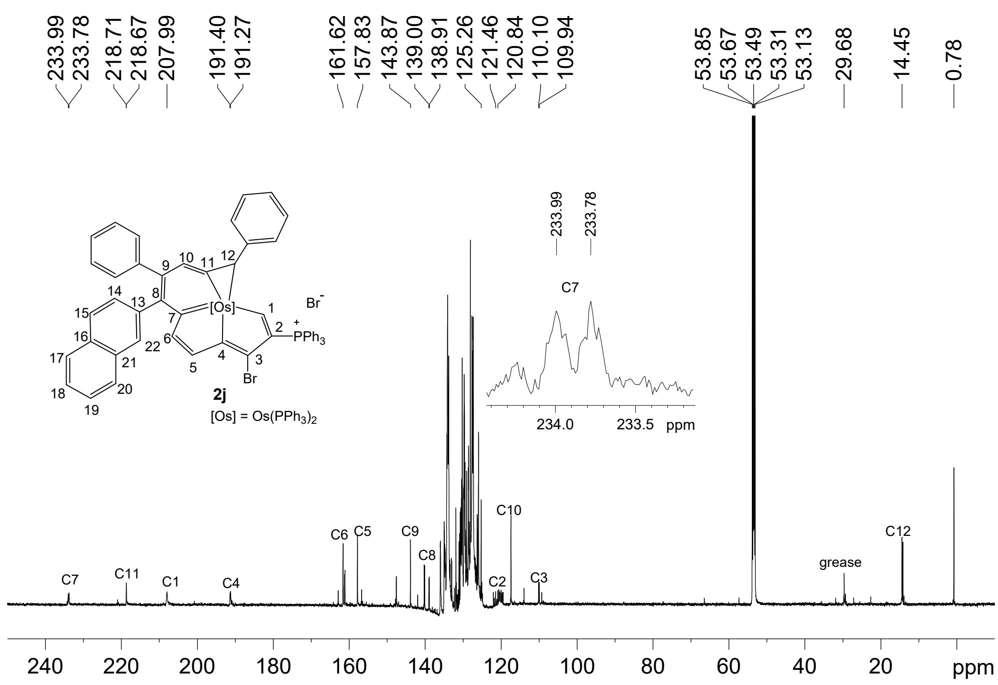


**Figure S39** The $^{13}C$ NMR (150.9 MHz, $CD_2Cl_2$) spectrum for complex **2j**.

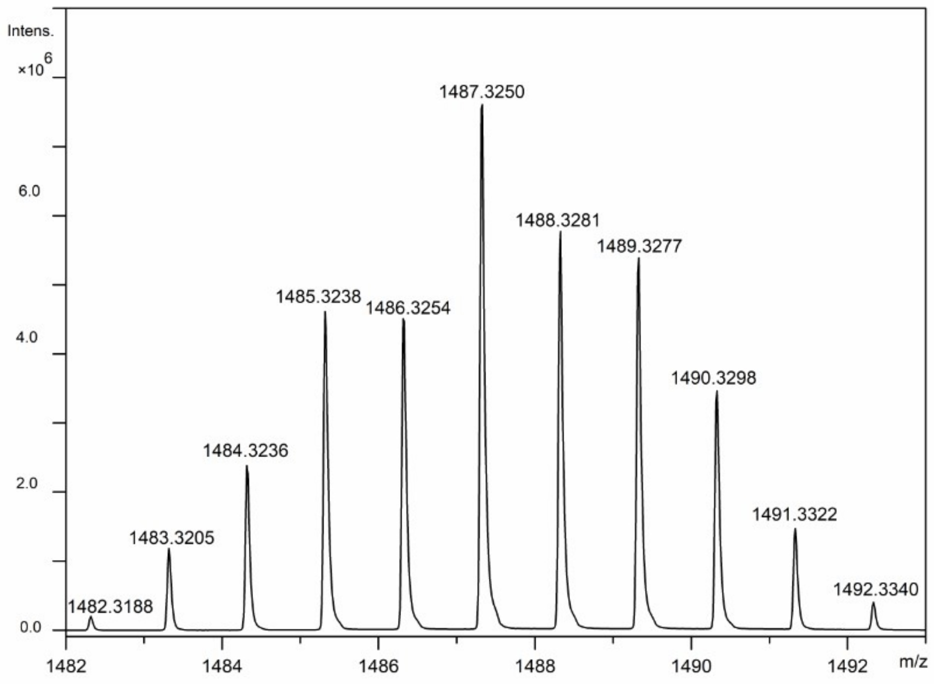


**Figure S40** Positive-ion ESI-MS spectrum of **2j** measured in methanol.

## 2. Ligand Screen

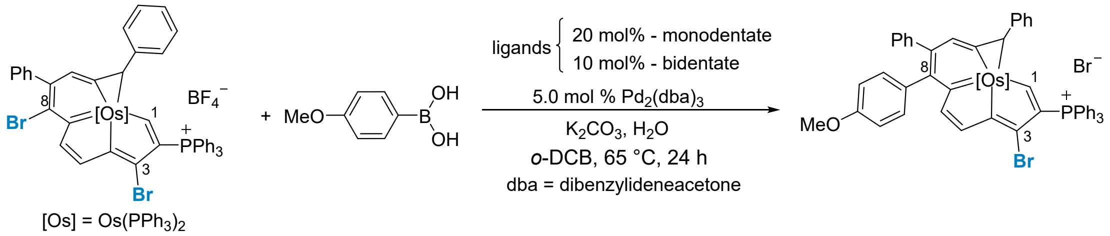


| entry | [a]ligands | Yield(%) |
|---|---|---|
| 1 | P($^t$Bu)$_3$ | 0 |
| 2 | P(*o*-tol)$_3$ | 0 |
| 3 | $PPh_3$ | 0 |
| 4 | DPPF | 0 |
| 5 | Sphos | 0 |
| 6 | BIPHEP | 0 |

Reaction conditions: **1** (0.1 mmol), 4-methoxyphenyl boronic acid (0.35 mmol), $K_2CO_3$ (0.2 mmol), $Pd_2(dba)_3$ (5.0 mol %), *o*-DCB (5 mL).
[a]Monodentate ligands were loaded at 20 mol %, bidentate at 10 mol %.

Ligand study: Reaction was performed according to the standard procedure for metalla-aromatics suzuki-miyaura coupling. The effect of additional phosphine ligands on the reaction was probed systematically, almost obtaining triphenylphosphine oxide and **2'**, no cross-coupling product **2g**.

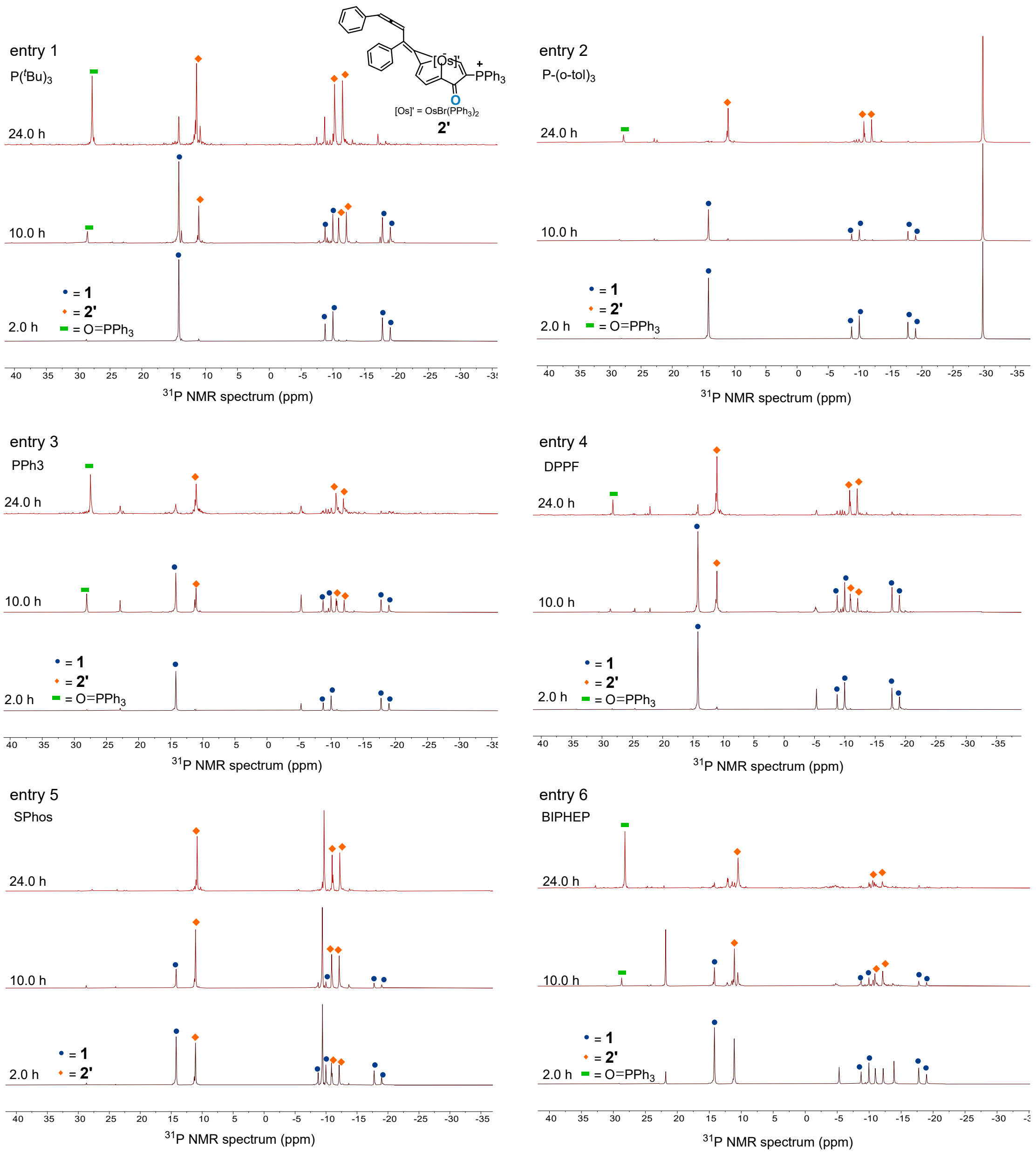


**Figure S41** Overlapping $^{31}P$ {$^1H$} NMR Spectrums.

### 3. Aryl Boronic Acid with Methoxy Group Located in Different Positions

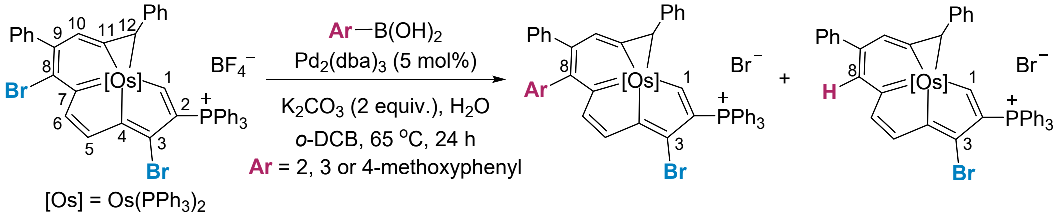


**1** **6** **7**

| entry | aryl boronic acid | conv. (%) | ratio (**6**/**7**) |
|---|---|---|---|
| 1 | (2-methoxyphenyl)boronic acid | 89 | 7/93 |
| 2 | (3-methoxyphenyl)boronic acid | 86 | 38/62 |
| 3 | (4-methoxyphenyl)boronic acid | 92 | 96/4 |

**Entry 1**: Reaction was performed according to the standard procedure for metalla-aromatics suzuki-miyaura coupling. (2-methoxyphenyl) boronic acid was used for the reaction, almost obtaining byproduct **7**. The ratio of product **6a** to byproduct **7** is 7/93 by integral of the characteristic hydrogen spectrum (H1).

**Entry 2**: Reaction was performed according to the standard procedure for metalla-aromatics suzuki-miyaura coupling. (3-methoxyphenyl) boronic acid was used for the reaction, byproduct **7** sharply decreasing. The ratio of product **6b** to byproduct **7** is 38/62 by integral of the characteristic hydrogen spectrum (H1).

**Entry 3**: Reaction was performed according to the standard procedure for metalla-aromatics suzuki-miyaura coupling. (4-methoxyphenyl) boronic acid was used for the reaction, almost obtaining product **6c**. The ratio of product **6c** to byproduct **7** is 96/4 by integral of the characteristic hydrogen spectrum (H1).

### 4. Isotopic-labeling Experiments

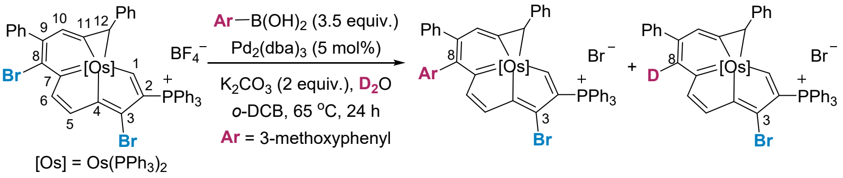


**1** **8** **9**

Reaction was performed according to the standard procedure for deuterium-labeling

experiments. In this case, $D_2O$ neat was used instead of regular $H_2O$. 100% deuterium incorporation has taken place at C8, affording byproduct **9**. **9** was characterized by high-resolution mass spectrometry (Figure S42).

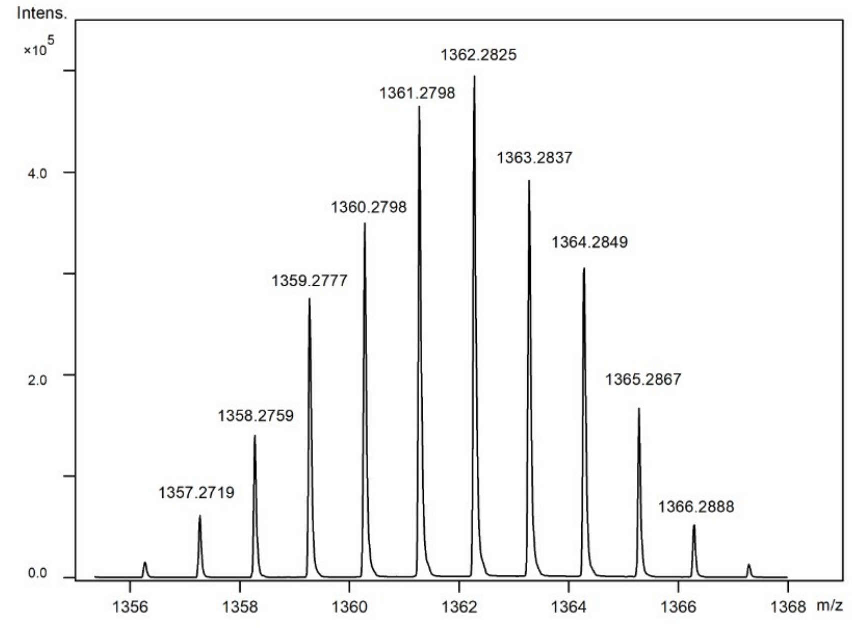


**Figure S42** Positive-ion ESI-MS spectrum of **9** measured in methanol

## 5. Reaction of 1 with (4-(trifluoromethyl)phenyl) boronic acid

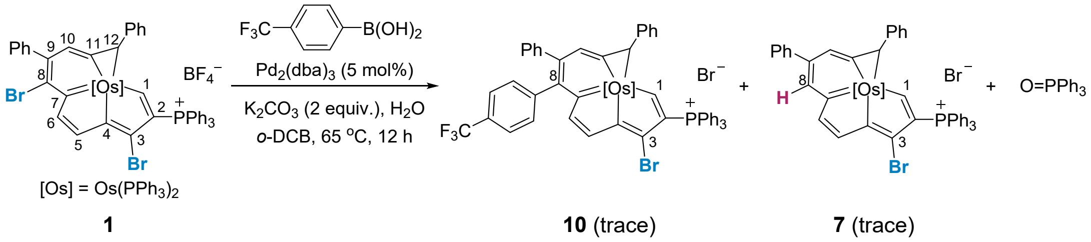


Reaction was performed according to the standard procedure for metalla-aromatics suzuki-miyaura coupling. (4-(trifluoromethyl)phenyl) boronic acid was used for the reaction, as monitored by in situ NMR (Figure S43), almost obtaining triphenylphosphine oxide, only trace cross-coupling product **10** and byproduct **7**. Cross-coupling product **10** was characterized by high-resolution mass spectrometry (Figure S44).

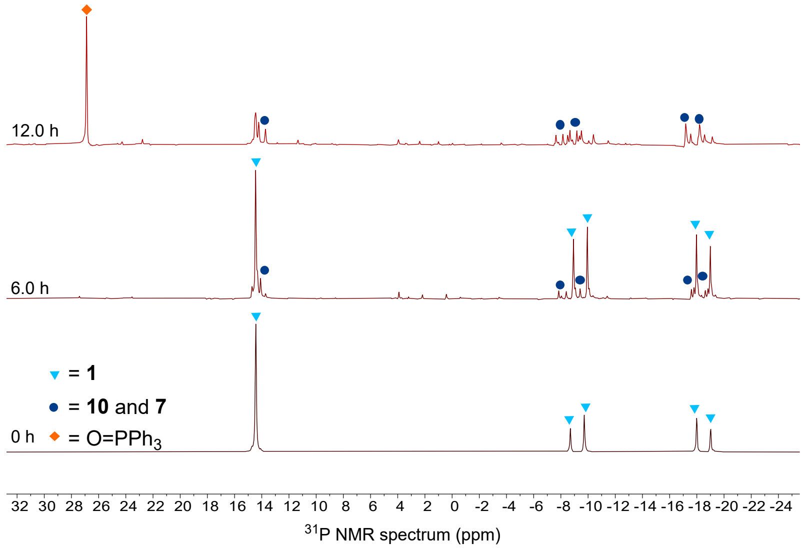


**Figure S43** Overlapping $^{31}P$ {$^{1}H$} NMR Spectrums.

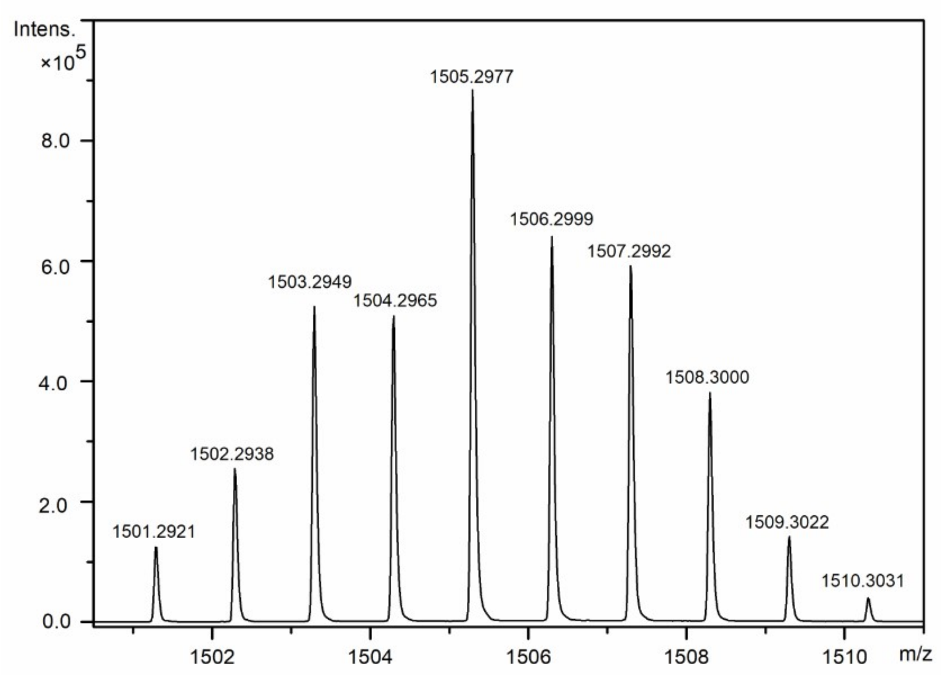


**Figure S44** Positive-ion ESI-MS spectrum of **10** measured in methanol

## 6. X-ray Crystallographic Analysis

All single crystals suitable for X-ray diffraction were grown from 1,2-dichloroethane solution layered with hexane unless otherwise stated. Single-Crystal X-ray diffraction data were collected on a Rigaku XtaLAB Synergy, Dualflex, HyPix diffractometer with mirror-monochromated Cu Kα radiation (λ = 1.54184 Å) for **2a** and **2h**. With Olex2 [1], the structures of **2a** and **2h** were solved with ShelXT[2] structure solution program using

Direct methods and refined with the ShelXL [3] refinement package using Least Squares minimization. All non-hydrogen atoms were refined anisotropically unless otherwise stated. Hydrogen atoms were placed at idealized positions and assumed the riding model. Some of the solvent molecules and phenyl groups were disordered and refined with suitable restrains. CCDC-2194944 (**2a**) and CCDC-2194946 (**2h**) contain supplementary crystallographic data for this paper. These data can be obtained free of charge from The Cambridge Crystallographic Data Centre.

**Table S1.** Crystallographic details for complex **2a** and **2h**

| | **2a** | **2h** |
|---|---|---|
| Empirical formula | $C_{86}H_{69}Br_2Cl_2OsP_3$ | $C_{98}H_{77}Br_2Cl_2NOsP_3$ |
| Formula weight | 1571.78 | 1782.43 |
| Temperature/K | 100.00(10) | 99.99(10) |
| Radiation | CuKα (1.54184) | CuKα (1.54184) |
| Crystal system | triclinic | monoclinic |
| Space group | P-1 | C2/c |
| a/Å | 13.8929(3) | 37.0917(4) |
| b/Å | 15.2075(3) | 10.98180(10) |
| c/Å | 18.0219(4) | 43.2112(5) |
| α/° | 93.513(2) | 90 |
| β/° | 96.836(2) | 106.0800(10) |
| γ/° | 101.444(2) | 90 |
| V/Å$^3$ | 3691.24(14) | 16912.7(3) |
| Z | 2 | 8 |
| $d_{calc}$/g cm$^{-3}$ | 1.414 | 1.400 |
| μ/mm$^{-1}$ | 5.848 | 5.428 |
| F(000) | 1584.0 | 7176.0 |
| Reflections collected | 100729 | 81743 |
| Independent reflections | 12300 | 14941 |
| Data/restraints/parameters | 12300/600/856 | 14941/132/1168 |
| Goodness-of-fit on $F_2$ | 1.064 | 1.063 |
| Final R indexes [I>=2σ(I)] | R1 = 0.0547, wR2 = 0.1540 | R1 = 0.0495, wR2 = 0.1288 |
| peak/hole/e Å$^{-3}$ | 1.84/-1.72 | 1.68/-1.67 |

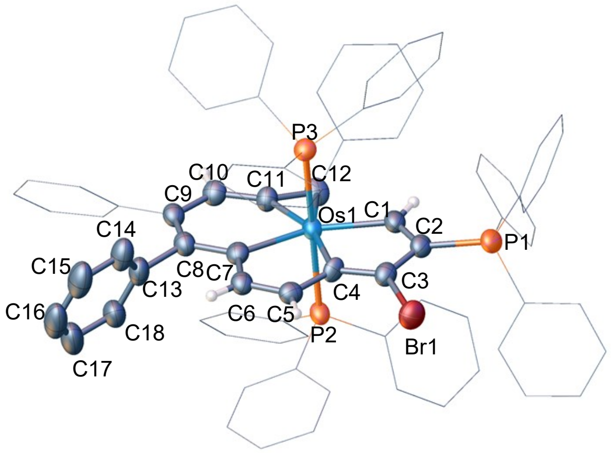


**Figure S45** X-ray molecular structure for the cation of complex **2a** drawn with 50% probability level. The hydrogen atoms on the phenyl group are omitted for clarity. Selected bond lengths [Å] and angles [°]: Os1–C1 2.094(6), Os1–C4 2.109(5), Os1–C7 2.110(6), Os1–C11 2.025(6), Os1–C12 2.238(5), C1–C2 1.356(9), C2–C3 1.437(8), C3–C4 1.351(8), C4–C5 1.410(8), C5–C6 1.346(8), C6–C7 1.419(8), C7–C8 1.457(8), C8–C9 1.378(8), C9–C10 1.439(8), C10–C11 1.338(9), C11–C12 1.391(8), C8–C13 1.513(8), C13–C14 1.380(10), C14–C15 1.380(10), C15–C16 1.351(13), C16–C17 1.356(13), C17–C18 1.404(10), C3–Br1 1.910(6).

Os1-C1-C2 119.0(4), C1-C2-C3 112.8(5), C2-C3-C4 115.8(5), C3-C4-Os1 117.3(4), C4-Os1-C1 74.9(2), Os1-C4-C5 114.9(4), C4-C5-C6 115.8 (5), C5-C6-C7 118.7(5), C6-C7-Os1 113.0(4), C7-Os1-C4 77.6(2), Os1-C7-C8 129.8(4), C7-C8-C9 124.2(5), C8-C9-C10 122.8(5), C9-C10-C11 123.3(6), C10-C11-Os1 136.9(4), C11-Os1-C7 82.6(2), Os1-C11-C12 79.5(3), C11-C12-Os1 62.8(3), C12-Os1-C11 37.7(2), P1-Os1-P2 171.49(5).

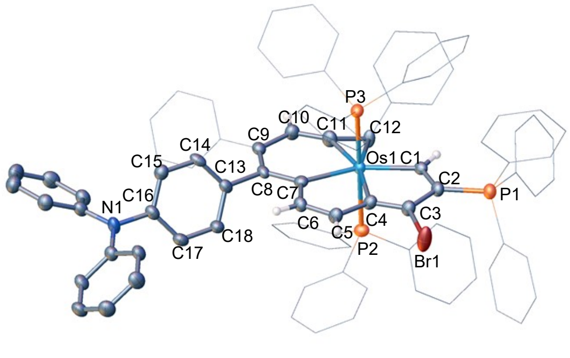


**Figure S46** X-ray molecular structure for the cation of complex **2h** drawn with 50%

probability level. The hydrogen atoms on the phenyl group is omitted are omitted for clarity. Selected bond lengths [Å] and angles [°]: Os1–C1 2.086(5), Os1–C4 2.113(5), Os1–C7 2.125(5), Os1–C11 2.022(5), Os1–C12 2.256(13), C1–C2 1.373(7), C2–C3 1.449(7), C3–C4 1.356(7), C4–C5 1.421(7), C5–C6 1.366(7), C6–C7 1.443(7), C7–C8 1.442(7), C8–C9 1.403(7), C9–C10 1.437(7), C10–C11 1.337(7), C11–C12 1.434(14), C8–C13 1.507(6), C13–C14 1.401(7), C14–C15 1.389(7), C15–C16 1.387(7), C16–C17 1.388(7), C17–C18 1.394(7), C16–N1 1.432(6), C3–Br1 1.924(5).

Os1-C1-C2 118.9(3), C1-C2-C3 112.4(4), C2-C3-C4 116.6(5), C3-C4-Os1 116.2(4), C4-Os1-C1 75.85(19), Os1-C4-C5 115.2(4), C4-C5-C6 115.7(4), C5-C6-C7 118.4(4), C6-C7-Os1 112.5(3), C7-Os1-C4 78.10(18), Os1-C7-C8 130.6(3), C7-C8-C9 123.7(4), C8-C9-C10 122.6(4), C9-C10-C11 123.0(5), C10-C11-Os1 138.2(4), C11-Os1-C7 81.89(19), Os1-C11-C12 79.5(6), C11-C12-Os1 61.8(5), C12-Os1-C11 38.7(3), P1-Os1-P2 171.35(4).

## 7. **Reaction of 3 with 4-methoxyphenyl boronic acid**

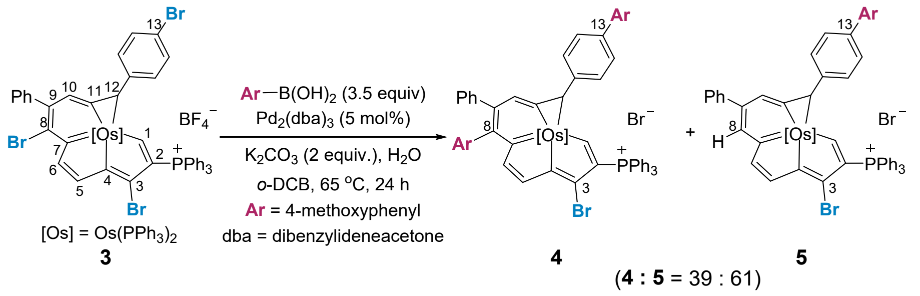


Reaction was performed according to common Suzuki-coupling reaction conditions. (4-methoxyphenyl) boronic acid was used for the reaction, as monitored by in situ NMR (Figure S47), obtaining product **4** and **5**. The ratio of product **4** to **5** is 39/61 by integral of the characteristic hydrogen spectrum (H1).

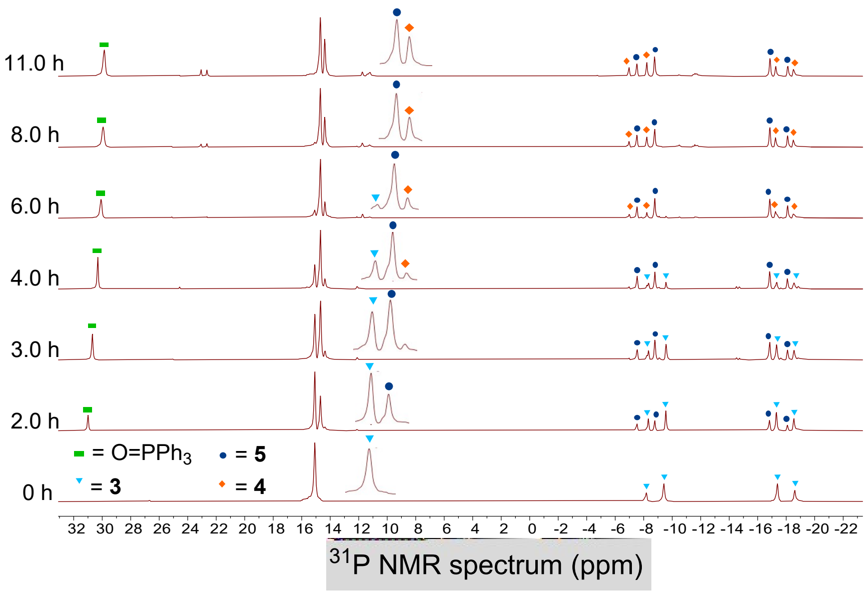


**Figure S47** Overlapping $^{31}$P {$^{1}$H} NMR Spectrums.

## 8. Reaction of 3 with 1 equiv. 4-methoxyphenyl boronic acid

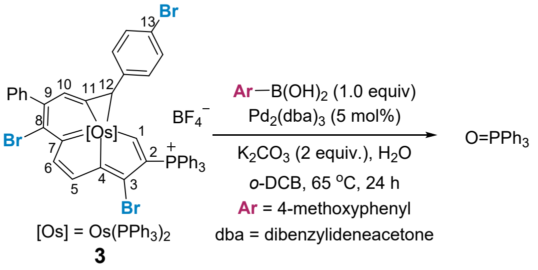


Reaction was performed according to common Suzuki-coupling reaction conditions. (4-methoxyphenyl) boronic acid (1 equiv.) was used for the reaction, as monitored by in situ NMR (Figure S48), almost obtaining triphenylphosphine oxide.

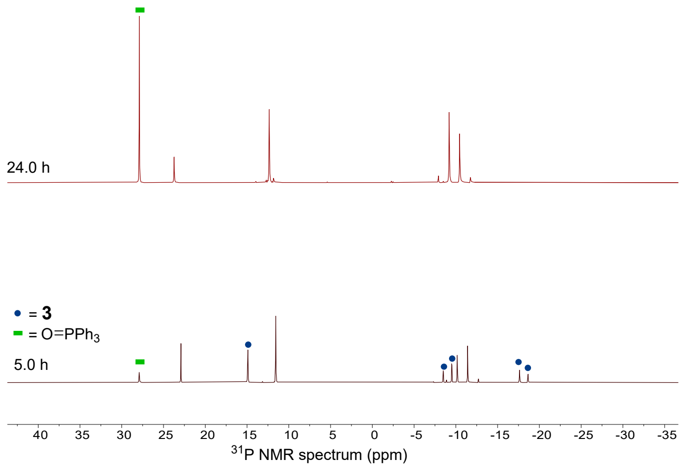


**Figure S48** Overlapping $^{31}$P {$^{1}$H} NMR Spectrums.

## 9. Reaction of 3 with 2.5 equiv. 4-methoxyphenyl boronic acid

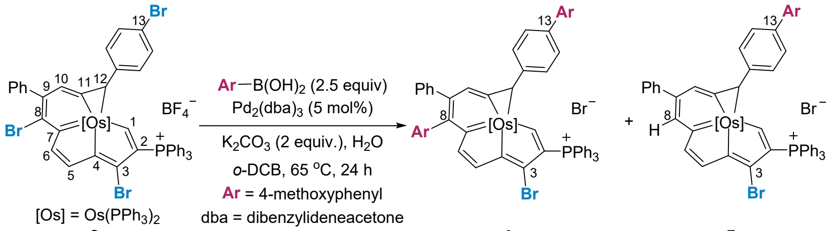


**3** **4** **5**

Reaction was performed according to common Suzuki-coupling reaction conditions. (4-methoxyphenyl) boronic acid (2.5 equiv.) was used for the reaction, as monitored by in situ NMR (Figure S49), obtaining product **4** and **5**.

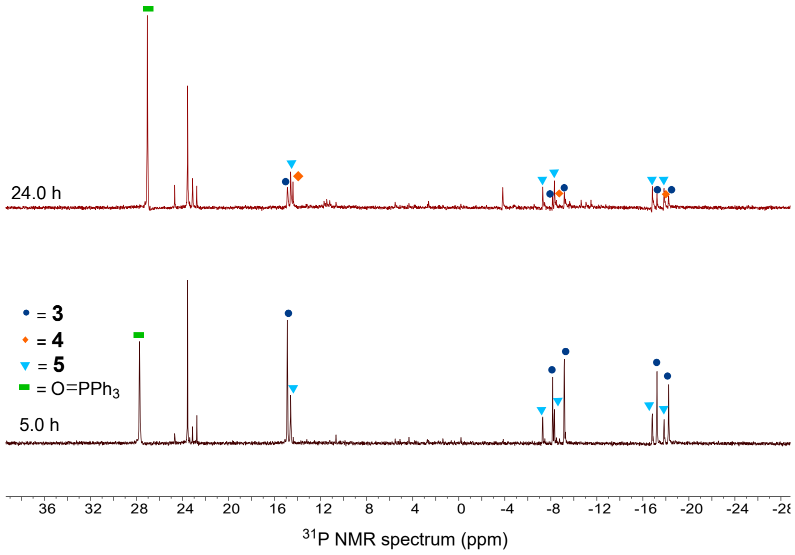


**Figure S49** Overlapping $^{31}P\ \{^{1}H\}$ NMR Spectrums.

## 10. Computational Calculations

All the calculations were performed with the Gaussian 09 software package[4] except the NICS calculations were performed with the Gaussian 16 software package.[5] All the structures were first optimized at B3LYP/6-31G(d) level of destiny functional theory.[6-8] The effective core potentials (ECPs) of Hay and Wadt with a double-ζ valence basis set (SDD) were used to describe Os, P and Br atoms. Polarization functions were added for Os (ζ(f) = 0.886), P (ζ(d) = 0.340), and Br (ζ(d) = 0.389).[8-10] Frequency calculations were performed at the same level of theory as for geometry optimization to characterize the stationary points as either minima (no imaginary

frequencies) or first-order saddle points (no imaginary frequencies) on the potential energy surface. The NICS and BDE were calculated at B3LYP/def2-TZVP[10-11] level of destiny functional theory. The fukui value were calculated at M06L/def2-TZVP level of destiny functional theory and analysed by Multiwfn. [12-13] The AICD were calculated at B3LYP/6-311++G(dp) level of destiny functional theory with the same basis set of the structural optimization.[13-14] For the reaction mechanism study, molecular geometries were optimized using the B3LYP functional. The SDD basis set was used for Os, Pd, Br, P and the 6-31G(d) basis set was used for all other atoms. The effective core potentials (ECPs) of Hay and Wadt with a double-ζ valence basis set for Os, Pd, Br, P are the same as above. The Frequency calculations were performed at the same level of theory as for geometry optimization to characterize the stationary points as either minima (no imaginary frequencies) or first-order saddle points (one imaginary frequency) on the potential energy surface. Intrinsic reaction coordinate calculations were performed to ensure that the first-order saddle points found were true transition states (TS) connecting the reactants and the products. Single-point energies were calculated with the B3LYP/def2-tzvp functional. Solvation effects were incorporated during geometry optimizations and Single-point energies using the PCM solvation model with *o*-DiChloroBenzene. Em = GD3 were used in the Single-point energy calculations.

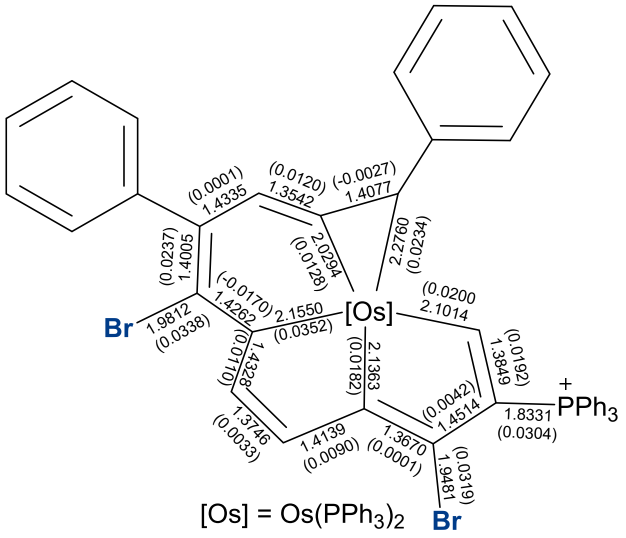


**1**

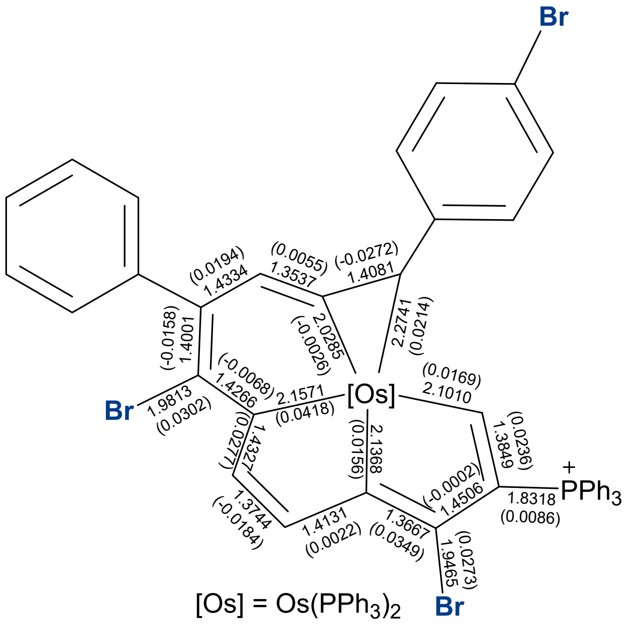


**3**

**Figure S50** The bond length chang about structural optimization of **1** and **3**. Outside the brackets is the bond length of optimized structure, and in parentheses is the bond length difference of optimized structure and crystal structure.

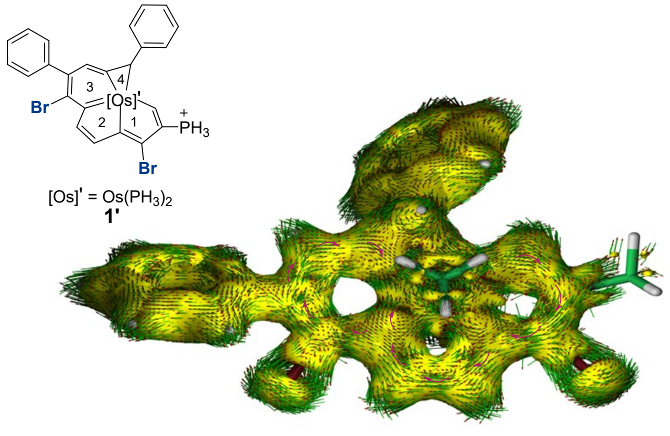

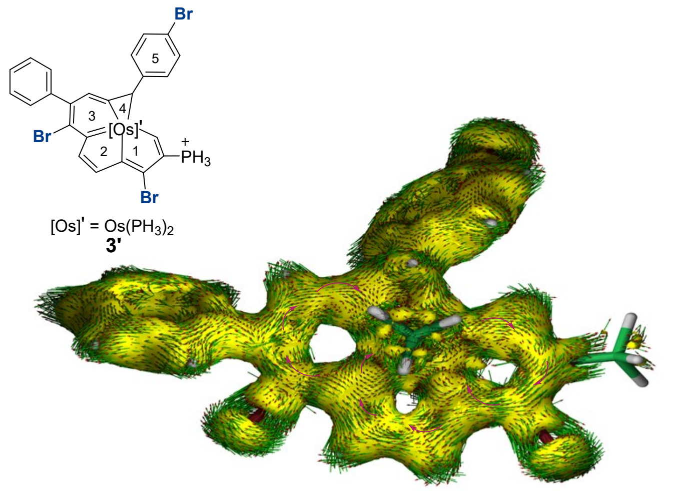


**Figure S51** AICD isosurfaces of **1'** and **3'**

## 11. Oxidative addition of 3-8C' and $PdL_2$ at C13

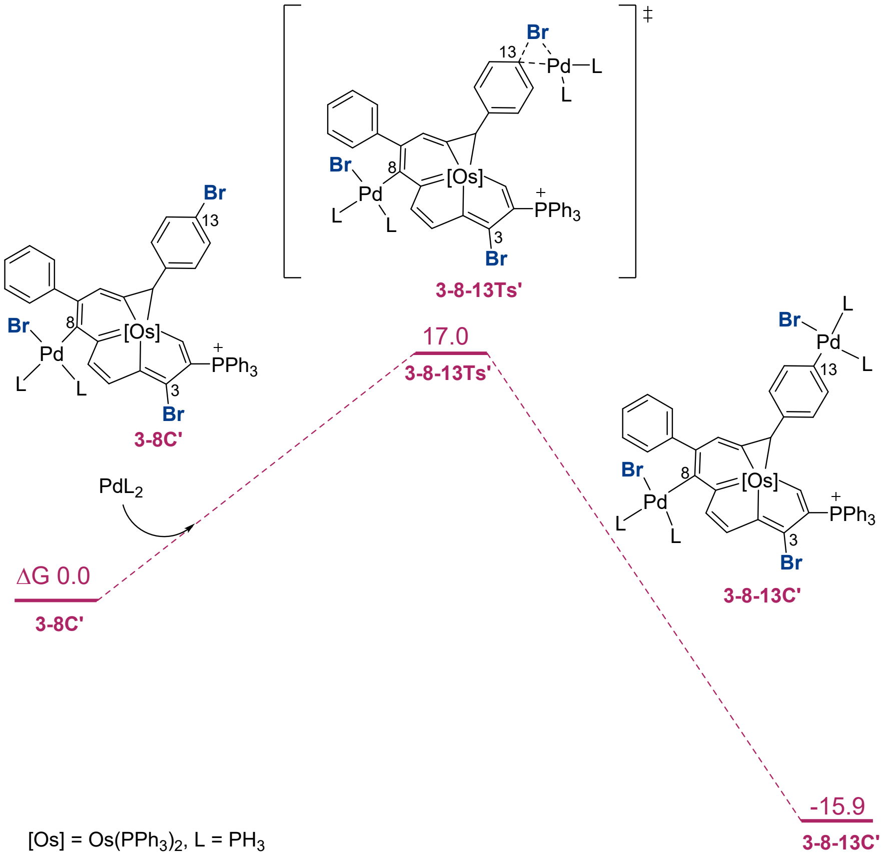


**Figure S52** DFT-computed Gibbs free energy profile for the oxidative addition steps. Reaction between **3-8C'** and $PdL_2$ at C13, L = $PH_3$. All energies were computed at the level B3LYP-D3/def2-TZVP/PCM(*o*-DCB)//B3LYP/6-31G(d)(SDD for Os, Br, P), and are given in kcal/mol.

## 12. Mix 1.0 equiv. compound 1 and 1.0 equiv. bromobenzene with 1.0 equiv. *p*-methoxyphenylboronic acid

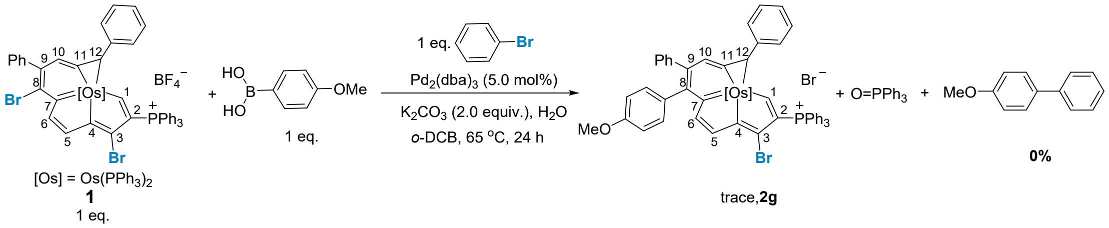


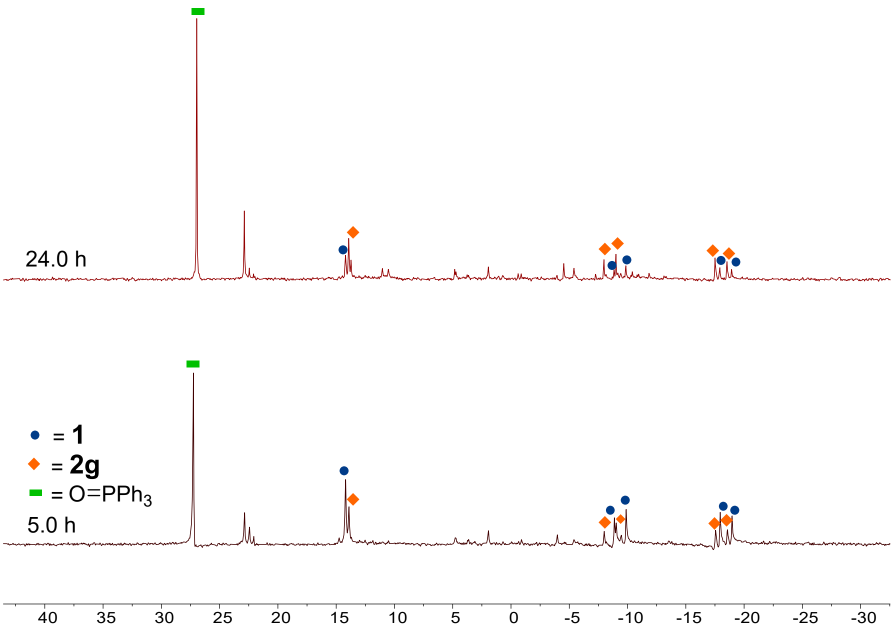


**Figure S53** Overlapping $^{31}P$ $\{^{1}H\}$ NMR Spectrums.

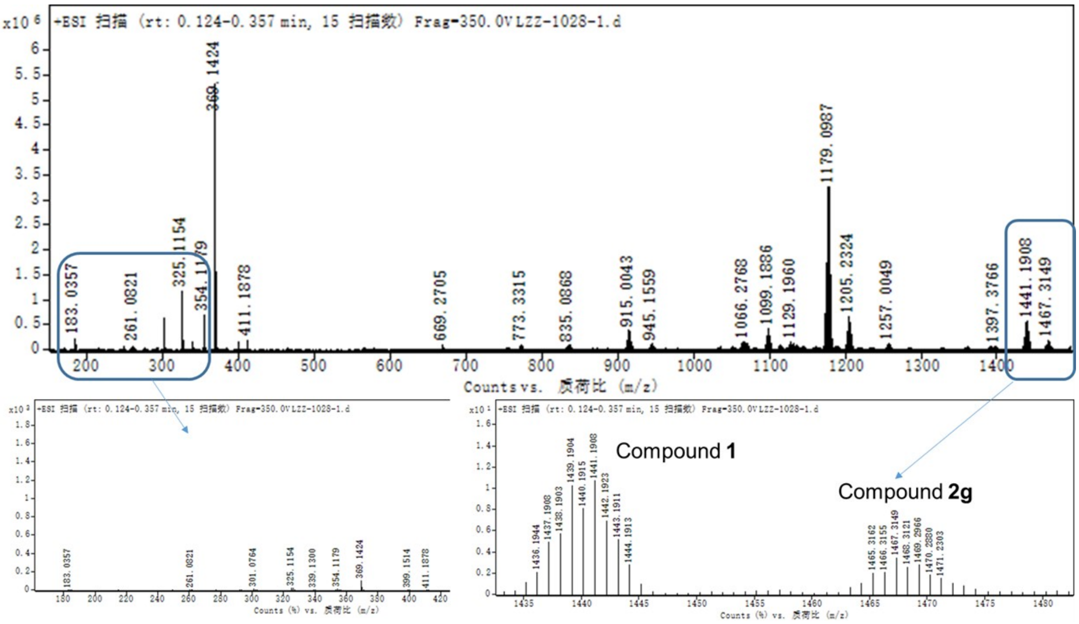


**Figure S54** In situ Positive-ion ESI-MS spectrum

## 13. Oxidative addition of 3 and PdL (L = $PH_3$)

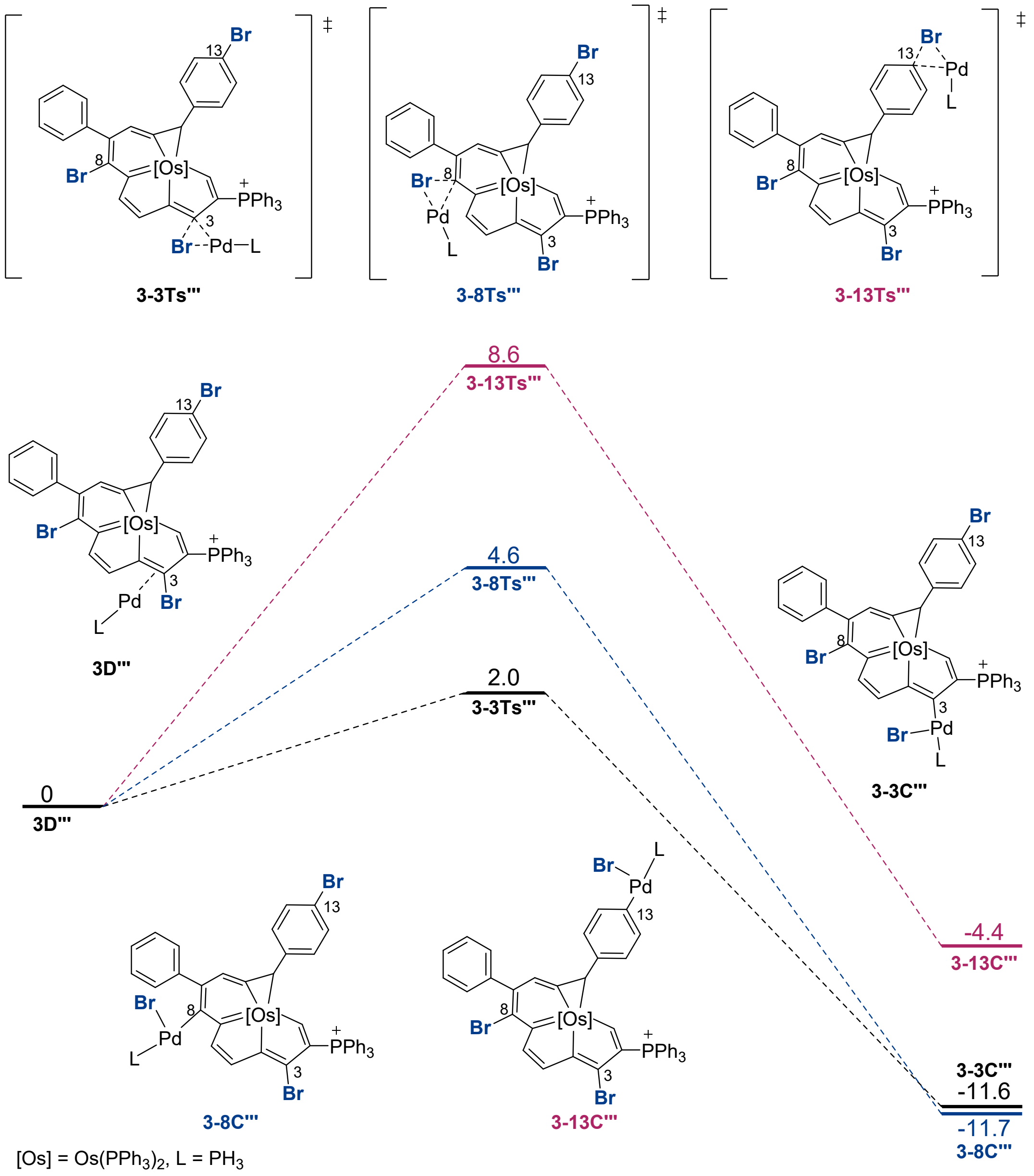


**Figure S55** DFT-computed Gibbs free energy profile for the oxidative addition steps. Reaction between **3** and PdL at C3 or C8 or C13, L = $PH_3$. All energies were computed at the level B3LYP-D3/def2-TZVP/PCM(*o*-DCB)//B3LYP/6-31G(d)(SDD for Os, Br, P), and are given in kcal/mol.

## 14. Oxidative addition of 3 and PdL (L = $PPh_3$)

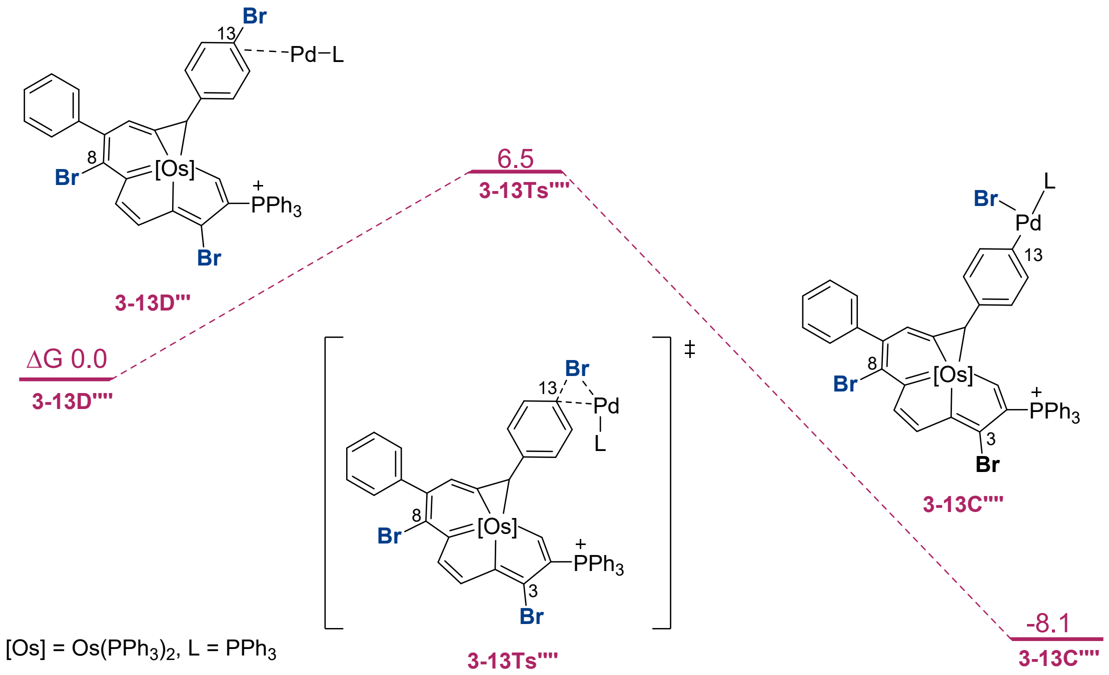


**Figure S56** DFT-computed Gibbs free energy profile for the oxidative addition steps. Reaction between **3** and PdL at C13, L = $PPh_3$. All energies were computed at the level B3LYP-D3/def2-TZVP/PCM(*o*-DCB)//B3LYP/6-31G(d)(SDD for Os, Br, P), and are given in kcal/mol.

## 15. The oxidative addition processes with the model chloro-substituted compounds (3'')

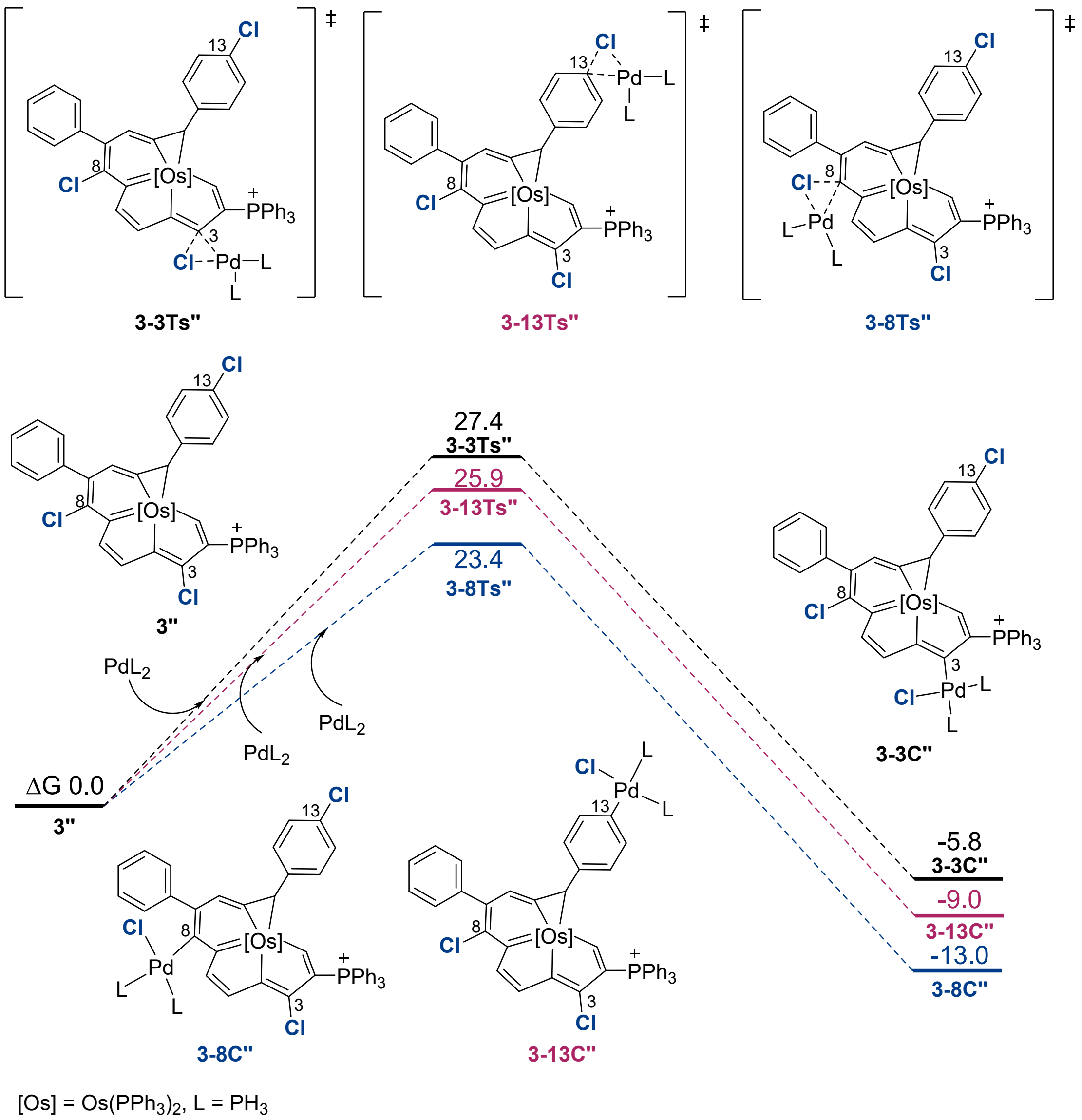


**Figure S57** DFT-computed Gibbs free energy profile for the oxidative addition steps. Reaction between **3''** and $PdL_2$ at C3 or C8 or C13, L = $PH_3$. All energies were computed at the level B3LYP-D3/def2-TZVP/PCM(*o*-DCB)//B3LYP/6-31G(d)(SDD for Os, Cl, P), and are given in kcal/mol.

## 16. Proposed Mechanism

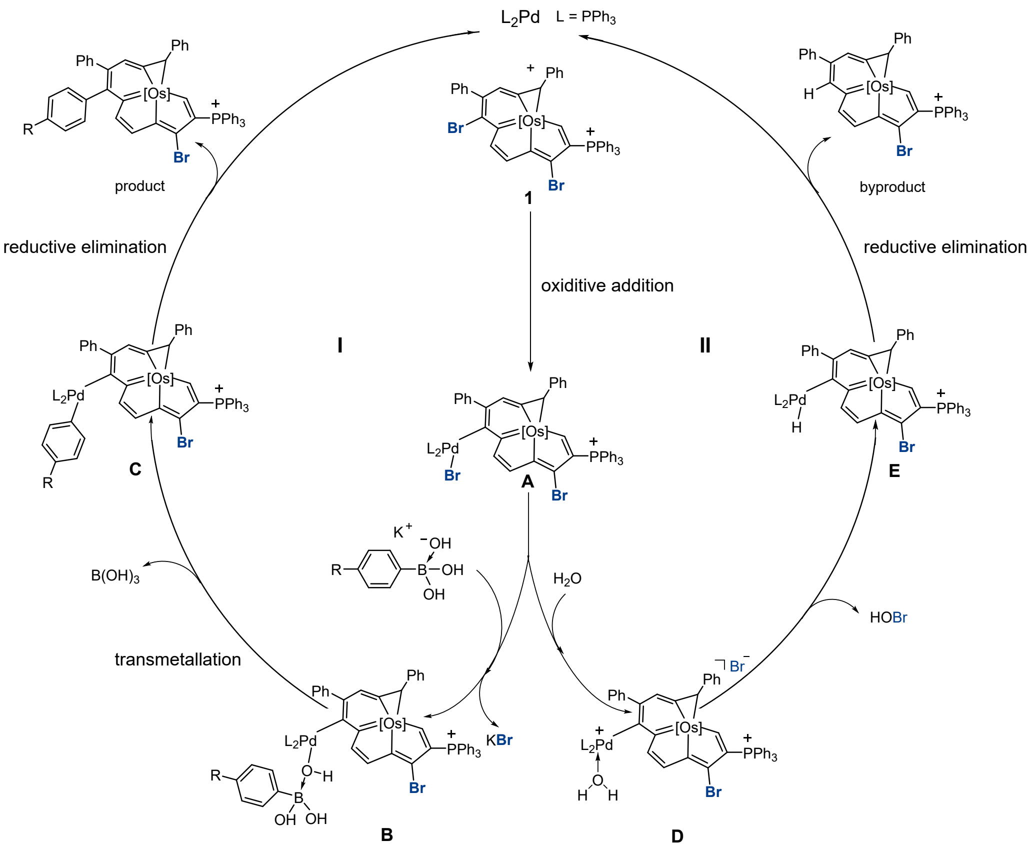


**Figure S58** Proposed mechanism for the formation of product and byproduct.

## 17. References


[1] G. M. Sheldrick, SHELXT–Integrated space-group and crystal-structure determination. *Acta. Cryst. Sect. A*. **2015**, *71*, 3-8.

[2] G. M. Sheldrick, Crystal structure refinement with SHELXL. Acta. Cryst. Sect. C. **2015**, *71*, 3-8.

[3] O. V. Dolomanov, L. J. Bourhis, R. J. Gildea, J. A. K. Howard, H. Puschmann, *Olex2*: a complete structure solution, refinement and analysis program. *J. Appl. Cryst.* **2009**, *42*, 339-341.

[4] Gaussian 09, Revision E.01, M. J. Frisch, G. W. Trucks, H. B. Schlegel, G. E. Scuseria, M. A. Robb, J. R. Cheeseman, G. Scalmani, V. Barone, B. Mennucci, G. A. Petersson, H. Nakatsuji, M. Caricato, X. Li, H. P. Hratchian, A. F. Izmaylov, J. Bloino, G. Zheng, J. L. Sonnenberg, M. Hada, M. Ehara, K. Toyota, R. Fukuda, J. Hasegawa, M. Ishida, T. Nakajima, Y. Honda, O. Kitao, H. Nakai, T. Vreven, J. A. Montgomery, Jr., J. E. Peralta, F. Ogliaro, M. Bearpark, J. J. Heyd, E. Brothers, K. N. Kudin, V. N. Staroverov, T. Keith, R. Kobayashi, J. Normand, K. Raghavachari, A. Rendell, J. C. Burant, S. S. Iyengar, J. Tomasi, M. Cossi, N. Rega, J. M. Millam, M. Klene, J. E. Knox, J. B. Cross, V. Bakken, C. Adamo, J. Jaramillo, R. Gomperts, R. E. Stratmann, O. Yazyev, A. J. Austin, R. Cammi, C. Pomelli, J. W. Ochterski, R. L. Martin, K. Morokuma, V. G. Zakrzewski, G. A. Voth, P. Salvador, J. J. Dannenberg, S. Dapprich, A. D. Daniels, O. Farkas, J. B. Foresman, J. V. Ortiz, J. Cioslowski, and D. J. Fox, Gaussian, Inc., Wallingford CT, **2013**.

[5] Gaussian 16, Revision A.03, M. J. Frisch, G. W. Trucks, H. B. Schlegel, G. E. Scuseria, M. A. Robb, J. R. Cheeseman, G. Scalmani, V. Barone, G. A. Petersson, H. Nakatsuji, X. Li, M. Caricato, A. V. Marenich, J. Bloino, B. G. Janesko, R. Gomperts, B. Mennucci, H. P. Hratchian, J. V. Ortiz, A. F. Izmaylov, J. L. Sonnenberg, D. Williams-Young, F. Ding, F. Lipparini, F. Egidi, J. Goings, B. Peng, A. Petrone, T. Henderson, D. Ranasinghe, V. G. Zakrzewski, J. Gao, N. Rega, G. Zheng, W. Liang, M. Hada, M. Ehara, K. Toyota, R. Fukuda, J.

Hasegawa, M. Ishida, T. Nakajima, Y. Honda, O. Kitao, H. Nakai, T. Vreven, K. Throssell, J. A. Montgomery, Jr., J. E. Peralta, F. Ogliaro, M. J. Bearpark, J. J. Heyd, E. N. Brothers, K. N. Kudin, V. N. Staroverov, T. A. Keith, R. Kobayashi, J. Normand, K. Raghavachari, A. P. Rendell, J. C. Burant, S. S. Iyengar, J. Tomasi, M. Cossi, J. M. Millam, M. Klene, C. Adamo, R. Cammi, J. W. Ochterski, R. L. Martin, K. Morokuma, O. Farkas, J. B. Foresman, and D. J. Fox, Gaussian, Inc., Wallingford CT, **2016**.

[6] A. D. Becke. *J. Chem. Phys.*, **1993**, *98*, 5648-5652.

[7] B. Miehlich, A. Savin, H. Stoll, H. Preuss, *Chem. Phys. Lett.*, **1989**, *157*, 200-206 .

[8] C. Lee, W. Yang, R. G. Parr, *Phys. Rev. B.*, **1988**, *37*, 785-789.

[9] P. J. Hay and W. R. Wadt, *J. Chem. Phys.*, **1985**, *82*, 270-283.

[10] W. R. Wadt and P. J. Hay, *J. Chem. Phys.*, **1985**, *82*, 284-298.

[11] P. J. Hay, W. R. Wadt, *J. Chem. Phys.*, **1985**, *82*, 299-310.

[12] Y. Zhao, D. G, Truhlar. Theor. Chem. Acc., 2008 120, 215-241.

[13] T. Lu, F. Chen, J. Comput. Chem., **2012**, *33*, 580-592.

[14] F. Weigend, R. Ahlrichs, *Phys. Chem. Chem. Phys.*, **2005**, *7*, 3297-3305.

[15] F. Weigend, *Phys. Chem. Chem. Phys.*, **2006**, *8*, 1057-1065.
